# The influence of forest types including native and non‐native tree species on soil macrofauna depends on site conditions

**DOI:** 10.1002/ece3.70311

**Published:** 2024-09-18

**Authors:** Ronja Wenglein, Jing‐Zhong Lu, Stefan Scheu

**Affiliations:** ^1^ J.‐F. Blumenbach Institute of Zoology and Anthropology University of Göttingen Göttingen Germany; ^2^ Centre of Biodiversity and Sustainable Land Use University of Göttingen Göttingen Germany

**Keywords:** Douglas fir, forest mixtures, macrofauna, non‐native species, soil fauna, stable isotopes

## Abstract

The ongoing climate change calls for managing forest ecosystems in temperate regions toward more drought‐resistant and climate‐resilient stands. Yet ecological consequences of management options such as planting non‐native tree species and mixing coniferous and deciduous tree species have been little studied, especially on soil animal communities, key in litter decomposition and pest control. Here, we investigated the taxonomic and trophic structure of soil macrofauna communities in five forest types including native European beech (*Fagus sylvatica*), range‐expanding Norway spruce (*Picea abies*) and non‐native Douglas fir (*Pseudotsuga menziesii*) as well as conifer‐beech mixtures across loamy and sandy sites in northern Germany. Abundance of primary decomposers (feeding predominantly on litter) was high in Douglas fir and beech forests, benefiting from less acidic soil and more favorable litter resources compared to spruce forests, while secondary decomposers (feeding predominantly on microorganisms and microbial residues) reached highest densities in spruce forests. Differences in abundance and species richness among forest types generally varied between regions and were most pronounced in Douglas fir of the sandy region. However, trophic guilds differed more between regions than between forest types, indicating that environmental factors outweigh the importance of forest type on soil macrofauna communities. The analysis of stable isotopes (δ^15^N and δ^13^C values) supported the general robustness in trophic position of macrofauna trophic guilds against variations in forest types and regions, but indicated reduced detrital shifts and food‐chain lengths in coniferous compared to European beech forests with mixtures mitigating these effects. Overall, for evaluating consequences of future forest management practices on the structure and functioning of soil animal communities, regional factors need to be considered, but in particular at loamy sites the taxonomic and trophic structure of soil macrofauna communities are resistant against changes in forest types.

## 1 INTRODUCTION

Temperate forests are facing increasing decline due to climate extremes and associated bark beetle outbreaks calling for adaptation of silvicultural practices. European forests consist in large of monocultures. To enhance their resilience to increasing temperature and extreme weather events, forests are increasingly managed toward mixed forests including the plantation of non‐native tree species (Brang et al., 2014). These management practices likely are associated with changes in the diversity and functioning of above‐ and belowground animal communities. However, their consequences for the structure of soil animal communities are still little understood (Ganault et al., 2021). Especially consequences of the conversion of monocultures into mixed forests including non‐native tree species on the taxonomic and functional composition of soil animals needs further investigation. Soil animals are important drivers of litter decomposition, nutrient mineralization and soil formation (Abd El‐Wakeil, 2015; Barnes et al., 2018; Gilbert et al., 2014; Nielsen, 2019), and also serve as major agents linking belowground and aboveground components of ecosystems (Bardgett & van der Putten, 2014; Sackett et al., 2010; Scheu, 2001).

The dominant tree species of forests in central Europe, Norway spruce (*Picea abies*), is heavily suffering from drought and associated bark beetle outbreaks (Krejza et al., 2021). Confronted with the dieback of Norway spruce, the non‐native, more drought resistant Douglas fir (*Pseudotsuga menziesii*) is increasingly considered as alternative (Vitali et al., 2017). Furthermore, as Douglas fir is less acidifying the soil compared to Norway spruce and its leaf litter is more palatable for soil organism than that of Norway spruce, the humus form in Douglas fir forests is of higher quality than in Norway spruce forests which is also reflected by enhanced nitrification (Kupka et al., 2013; Podrázský et al., 2020). Nevertheless, the impact of Douglas fir on soils and soil organisms is still little known and depends on site conditions (Cremer & Prietzel, 2017; Lu & Scheu, 2021; Mrak et al., 2024). For example, microbial biomass has been shown to be significantly reduced in Doulgas fir at sandy but not all loamy sites compared to native European beech forests (Lu & Scheu, 2021). As is typical for introduced tree species, Doulas fir is colonized by only few, mostly generalist herbivore species (Schmid et al., 2014). Concerning soil animals, the abundance and activity of soil mesofauna has been found to be higher in Norway spruce than in Douglas fir forests (Kohlert & Roth, 2000; Rożen et al., 2010), whereas the abundance of macrofauna decomposers has been found to increase in Douglas fir compared to Norway spruce forests (Engel, 2001). However, little is known on soil macrofauna communities and their trophic organization in mixed stands including Douglas fir.

Since monocultures of conifers are increasingly being replaced by mixed stands of conifers and broadleaf species, in particular European beech (*Fagus silvatica*), changes due to mixing tree species are becoming increasingly relevant. It has been shown that the decomposition of litter in mixed spruce‐beech forests is faster than in pure spruce stands, independent of leaf/needle litter species (Berger & Berger, 2014). Mixed stands of conifers and beech are intermediate between the respective monocultures in storing soil organic carbon (Cremer et al., 2016). Furthermore, mixing conifers with beech reduces acidification (Foltran et al., 2023) and therefore may improve stand conditions. However, shifts in soil chemistry in mixed stands of European beech and Douglas fir are similar to those in mixed stands of European beech and Norway spruce (Foltran et al., 2020) but site conditions are playing an important role (Thurm & Pretzsch, 2016). Generally, increasing tree species richness has been found to increase the diversity of soil organisms (Ganault et al., 2021). However, abundance and functional diversity of ground beetles in mixed stands of beech and conifers were intermediate between the respective pure stands (Kriegel et al., 2021). Similarly, the biomass of different macrofauna groups in mixed conifer‐beech stands was intermediate between the respective pure stands (Scheu et al., 2003). More research on a wider spectrum of taxa and considering different site conditions is needed to explore if increased tree diversity and mixing of tree species increases soil fauna diversity.

Soil macrofauna species are an important component of forest ecosystems significantly contributing to leaf litter decomposition (Gongalsky, 2021; Slade & Riutta, 2012). Since macrofauna species are sensitive to variations in habitat conditions, it is important to understand their response to planting non‐native tree species such as Douglas fir and to the plantation of mixed forests (Eggleton et al., 2005; Wu & Wang, 2019). Although soil animal diversity is resilient against forest management practices, macrofauna detritivores have been shown to sensitively respond to changes in environmental conditions such as pH (Pollierer et al., 2021). Also, the abundance and diversity of litter dwelling macrofauna predators such as spiders have been shown to sensitively respond to the plantation of Douglas fir forests (Kriegel et al., 2021; Matevski & Schuldt, 2021; Matevski & Schuldt, 2023).

Changes in forest types lead to changes in habitat conditions and supply of resources likely affecting the trophic structure of soil animal communities. The analysis of natural variations in stable isotope ratios of carbon (^13^C/^12^C) and nitrogen (^15^N/^14^N) is increasingly used for characterizing trophic niches of species. The method allows insight into the trophic position of species (^15^N/^14^N ratios) as well as the use of basal resources (^13^C/^12^C ratios) (Potapov et al., 2019). Tree species affect soil organic matter turnover and are associated with different litter and root stable isotope signatures (Lorenz et al. 2020). Furthermore, litter quality may influence stable isotope signatures of soil fauna guilds as indicated by different ^13^C/^12^C ratios in coniferous compared to beech forests (Klarner et al., 2014). Also, the trophic position of generalist predators has been shown to be lower in Douglas fir than in beech forests (Wildermuth et al., 2023), but the influence of Douglas fir on the trophic structure of soil macrofauna communities has not been examined.

Here we analyze the effect of Douglas fir compared to Norway spruce in monoculture and in mixture with European beech on macrofauna communities and their tropic niches. Three guilds spanning a wide range of trophic levels including primary decomposers, secondary decomposers and predators are investigated. We aimed at investigation the response of abundance, species richness and guild structure of macrofauna to the plantation of non‐native Douglas fir monocultures and mixtures with European beech, in comparison to monocultures of Norway spruce and European beech as well as mixtures of European beech and Norway spruce. To investigate the generality of the response, we studied forest stands differing in site conditions, i.e. loamy and sandy sites. Shifts in trophic niches of macrofauna communities were studied using natural variations in stable isotope ratios of carbon and nitrogen to investigate differences between forest types and sites.

We hypothesize that (1) the abundance and species richness of macrofauna guilds decreases from beech to spruce to Douglas fir forests, with the differences being more pronounced in sandy compared to loamy sites, (2) in mixed forests of spruce and beech as well as Douglas fir and beech the abundance of macrofauna is intermediate between the respective monocultures but species richness is higher, (3) macrofauna communities of Douglas fir and Douglas fir/beech mixtures predominantly comprise generalist species and are similar to those in beech/spruce mixtures, and (4) the trophic position of macrofauna species (^15^N/^14^N ratios) is little affected by forest type, while the use of basal resources (^13^C/^12^C ratios) differs among stand types as well as between sandy and loamy sites.

## 2 MATERIAL AND METHODS

### 2.1 Study sites

We investigated a total of eight study sites each composed of five forest types (quintets) located in northern Germany grouped into four sandy and four loamy sites. The four loamy sites are characterized by more fertile partly podsolic Cambisols and Luvisols. The four sandy sites are characterized by Podzols on out‐washed sand. The mean annual precipitation at the four loamy sites is 821–1029 mm while at the four sandy sites it is 627–746 mm. More detailed information on site characteristics is given in Ammer et al. (2020) and Foltran et al. (2020). The five different forest types at each site included monocultures of European beech (*F. silvatica*), Douglas fir (*P. menziesii*) and Norway spruce (*P. abies*), as well as Douglas fir/European beech and Norway spruce/European beech mixtures. The average age of trees was 80 years (Glatthorn et al., 2023). The distance between forest type stands within sites ranged from 76 m to 4600 m, the sites were between 5 and 190 km apart. Plots of 50 m × 50 m were established in each stand. In pure stands, more than 90% of total basal area comprised the target tree species; in mixed stands, the basal area of beech ranged 33%–53% and of conifers 53–60%.

### 2.2 Animal extraction and identification

Samples were taken between November 2017 and January 2018. Two soil cores of 20 cm diameter were taken at a distance of approximately 10 m per plot using a metal cylinder and separated into litter layer and two soil layers, 0–5 and 5–10 cm soil depth. Temporal variations in soil fauna biomass are only moderate in temperate forests (Schaefer, 1990), likely due to litter and soil buffering climate extremes. Macrofauna was extracted from these layers by heat using a high‐gradient extractor for seven to 10 days (Kempson et al., 1963). Animals were collected in 50% diethylene glycol, filtered through 200 μm mesh and transferred into 70% ethanol. Macrofauna were sorted into broad taxonomic groups (Araneida, Chilopoda, Coleoptera, Coleoptera larvae, Diplopoda, Diptera larvae, Isopoda, Lumbricidae, Pseudoscorpionida and rest). Chilopoda, Coleoptera, Diplopoda and Isopoda were identified to species level if possible and Coleoptera larvae to family level using keys of Eason (1964), Klausnitzer (1978), Hopkin (1991), Lohse and Lucht (1999), Lompe (2002), Bährmann (2008) and Decker et al. (2024). Araneidae and Lumbricidae were not determined to species due to partial degrading during storage. The two samples per plot were pooled at each layer and species richness and abundance were calculated from data pooled across layers. Juveniles were only included into community analysis.

The animal taxa were assigned to trophic guilds based on litter corrected δ^15^N values (see Table S1) with corrections from literature for the taxa of Carabidae larvae, Geophilidae and Lithobidae (Bonato et al., 2021). Species without available δ^15^N values were ascribed to trophic guilds according to literature (Lawrence and Newton 1980; Arnett, 2002; Irmler et al., 2018; Schaefer, 2018).

### 2.3 Stable isotope analysis

Natural variations in bulk stable isotope values of ^15^N and ^13^C were used to quantify trophic niches of macrofauna taxa. If possible one individual per plot was measured for Aleocharinae larvae, Cantheridae larvae, Chilopoda, Diplopoda, Elateridae larvae, Isopoda and Staphylinidae larvae, with one individual per species kept as voucher. From the rest of Coleoptera larvae and Coleoptera adults, a maximum of three individuals per species was analyzed. Individuals were freeze dried overnight and then stored in a desiccator with silica gel. Samples >1 mg were ground and homogenized using mortar and pestle, and a subsample was taken for stable isotope analysis. The dry weight of all individuals used for stable isotope analysis was measured and used for biomass calculations. Samples were weighed into tin capsules, and stable isotope values were measured using a coupled system of an elemental analyzer (Flash 2000, Thermo Fisher Scientific, Massachusetts, USA) and a mass spectrometer (Delta V Advantage, Thermo Electron, Bremen, Germany). If individual samples were below 100 μg dry weight, a modified setup for small sample size was used (Langel & Dyckmans, 2014). Atmospheric nitrogen and Vienna PeeDee belemnite were used as primary standard, acetanilide (C8H9NO, Merck, Darmstadt) was used as internal standard.

Natural variations in stable isotope ratios of carbon and nitrogen (δX) were expressed as δX (‰) = (Rsample‐Rstandard)/Rstandard х 1000, with R being the ratio between the heavy and light isotopes (^13^C/^12^C or ^15^N/^14^N). Animal stable isotope values were calibrated to that of leaf litter at plot level (Klarner et al., 2014); stable isotope values of litter were taken from Lu et al. (2022). Litter corrected δ^13^C and δ^15^N values, denoted as Δ^13^C and Δ^15^N values, were used for statistical analysis.

### 2.4 Statistical analysis

All analyses were performed in R 4.4.0 (R Core Team, 2024). We used linear mixed models (LMMs) for macrofauna community and generalized linear mixed models (GLMMs) to analyze the effect of forest type (Douglas fir, Douglas fir/European beech, European beech, Norway spruce/European beech, Norway spruce), region (sandy, loamy) and their interaction on macrofauna guilds. Generalized linear mixed models with a ‘poisson’ distribution were selected as the best fit for the data. The eight sites were included as random effect. The response variables included abundance and richness of the respective guilds. Specimens from the three layers per soil core were summed as one community to focus on the factors of interest. To check for normality and homoscedasticity, model residuals were plotted.

To analyze the species‐based community structure of the different guilds, nonmetric multidimensional scaling (NMDS) and permutational multivariate analyses of variance (PERMANOVA) were used with Bray‐Curtis dissimilarities. Only taxa occurring in more than one plot were included into the analysis. Environmental variables (soil pH, soil carbon content, C/N ratio, water content, PLFA proportion of Gram^+^ and Gram^−^ bacteria and fungi, litter mass; Lu & Scheu, 2021) that may influence macrofauna community structure were selected through stepwise forward selection and fitted into NMDS using the *envfit* function.

Isotopic metrics (Layman et al. 2007, Cucherousset & Villéger 2015) were used to analyze trophic structure of the macrofauna and guilds. Values were scaled between 0 and 1 to place species in a two‐dimensional space of Δ^15^N and Δ^13^C values. For guilds, to reach the minimum number of replicates (>2) for the analysis, loamy and sandy sites were combined, resulting in a total of 10 plots for secondary decomposers and predators and 9 plots for primary decomposers. For primary decomposers, beech at loamy sites was excluded from the analysis as not enough samples were available to calculate multidimensional metrics. Single dimensional metrics included minimum, maximum and range calculated as biomass weighted means of Δ^15^N and Δ^13^C values. Five biomass weighted multidimensional isotopic metrics (Isotopic Divergence (IDiv), Isotopic Dispersion (IDis), Isotpic Evenness (IEve), Isotopic Uniqueness (IUni) and Isotopic Richness (IRic)) were calculated. IDiv measures the distance between species and the center of the convex hull area. Values close to 0 indicate that extreme values are rare in the community, values close to 1 represent a community dominated by extreme values. IDis combines IDiv and the convex hull area. IDis equals 0 if all species have the same isotopic values and tends to 1 when contrasting stable isotope values are abundant and far from the center of gravity. IEve quantifies the species distribution in the stable isotope space. IEve is close to 1 when community values are evenly distributed in space, while IEve tends to 0 when species are packed in clusters. IUni evaluates the closeness of isotopic values across the community. IUni values tend to 0 when many species share the same isotopic position and IUni tends to 1 when species are unique in their isotopic values. IRic represents the level of trophic diversity of all species in the community based on isotopic niches scaled from 0 to 1. More detailed information on the isotopic metrics is given in Cucherousset and Villéger (2015). Linear models were fitted for metrics and guilds with forest type and region as factors. The post hoc *HSD.test* function was used for inspecting differences among forest types and regions. For LMM, the *lmer* and *glmer* function of the ‘lme4’ package was used (Bates et al., 2015), and for NMDS and PERMANOVA the *metaNMDS* and *adonis2* function of the ‘vegan’ package was used (Oksanen et al. 2022). For pairwise comparisons, the *glht* function of the ‘multcomp’ package was used (Hothorn et al., 2008). Graphics were generated with ‘ggplot2’ (Wickham, 2016).

## 3 RESULTS

A total of 3003 individuals including 80 juveniles were analyzed. Primary decomposers comprised 183 individuals and 21 taxa, while secondary decomposers comprised 1909 individuals and 48 taxa and predators comprised 911 individuals and 56 taxa. Elateridae larvae was the most abundant taxon accounting for 25% of total individuals followed by Cantharidae larvae accounting for 19% of total individuals. Fourty‐three taxa were only found once and 21 taxa were only found twice.

**FIGURE 1:**
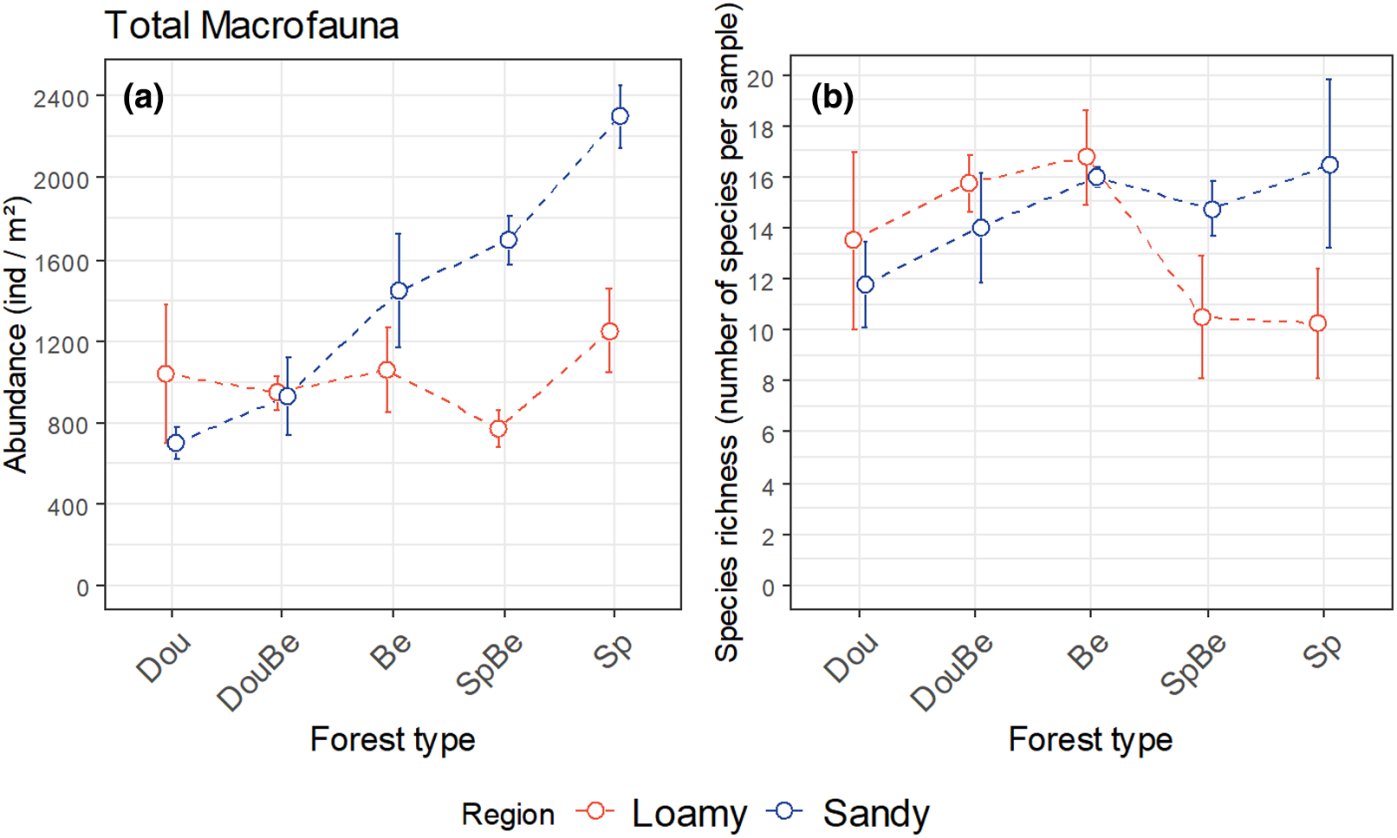
Abundance (ind/m^2^) (a) and species richness (number of species per sample) (b) of total macrofauna in five forest types [Douglas fir (Dou), Douglas fir‐beech mixture (DouBe), beech (Be), spruce‐beech mixture (SpBe), spruce (Sp)] and two regions (sandy, loamy), means ± SE.

Abundance of total macrofauna differed significantly between forest types but this varied between regions; at sandy sites the abundance was highest in Norway spruce forests and declined to European beech to Doulgas fir forests, with the respective mixed forests being intermediate, whereas at loamy sites the abundance was similar in each of the forest types (Figure 1a, Table 1). Overall, macrofauna species richness did not differ significantly between regions but among forest types; it was highest in beech and lower in Douglas fir and spruce forests, with mixtures being in between the pure stands (Figure 1b, Table 1).

**TABLE 1:** Generalized linear mixed effects models with Poission distribution of the effect of forest type, region and their interaction on the abundance and species richness of total macrofauna and three macrofauna guilds (primary decomposers, secondary decomposers and predators).

Abundance		Total macrofauna		Primary decomposers		Secondary decomposers		Predator	
	*df*	*X* ^2^	*p*‐value	*X* ^2^	*p*‐value	*X* ^2^	*p*‐value	*X* ^2^	*p*‐value
Intercept	1	**1533.09**	**<.001**	**7.30**	**.007**	**410.74**	**<.001**	**68.99**	**<.001**
Forest type (F)	4	**29.79**	**<.001**	**20.78**	**<.001**	**273.23**	**<.001**	**73.96**	**<.001**
Region (R)	1	**5.80**	**.016**	0.09	.768	**5.52**	**.019**	0.22	.635
F x R	4	**112.44**	**<.001**	**15.40**	**.004**	**77.22**	**<.001**	**47.04**	**<.001**

Species richness		Total macrofauna		Primary decomposer		Secondary decomposers		Predator	
	*df*	*X* ^2^	*p*‐value	*X* ^2^	*p*‐value	*X* ^2^	*p*‐value	*X* ^2^	*p*‐value
Intercept	1	**361.69**	**<.001**	0.25	.618			**12.76**	**<.001**
Forest type (F)	4	**10.29**	**.036**	6.05	.195	1.15	0.887	9.40	.052
Region (R)	1	0.48	.488	0.09	.763	0.16	0.691	2.06	.152
F x R	4	8.44	.077	3.45	.486	1.86	0.7614	**16.30**	**.003**

*Note*: *X* ^2^‐ and *p*‐values of type llI Anovas; significant effects are given in bold.

Abbreviation: *df*, degrees of freedom.

### 3.1 Abundance and species richness of trophic guilds

**FIGURE 2:**
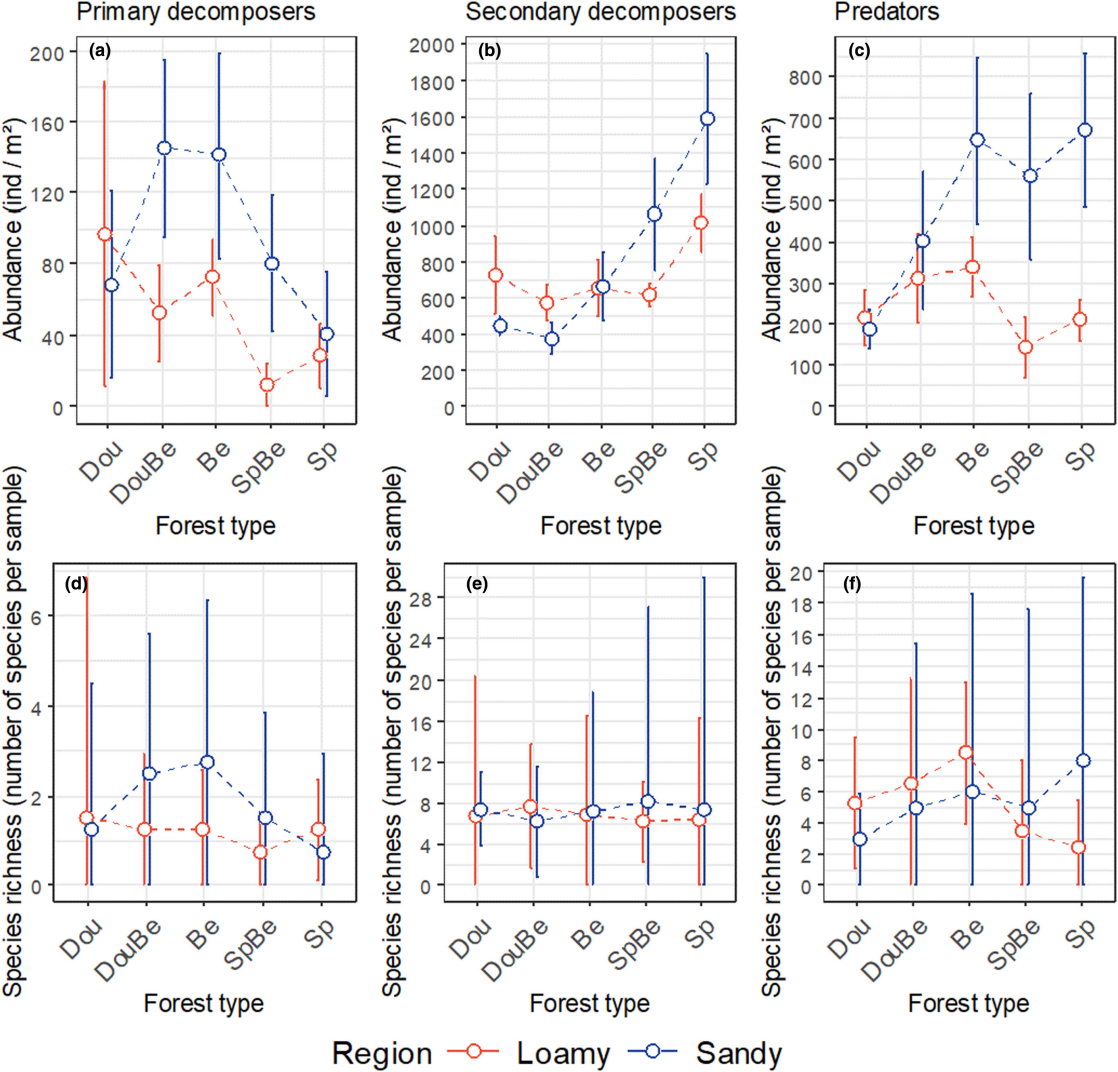
Abundance (ind/m^2^) and species richness (number of species per sample) for three trophic guilds (primary decomposers, secondary decomposers, predators) in five forest types [Douglas fir (Dou), Douglas fir‐beech mixture (DouBe), beech (Be), spruce‐beech mixture (SpBe), spruce (Sp)] in two regions (sandy, loamy), means ± SE.

Across forest types and regions, the abundance of the three trophic guilds differed significantly (ANOVA: F_2,14_ = 38.6, *p* < .001); it increased from primary decomposers (74 ± 14 ind/m^2^; mean ± SE) to predators (367 ± 47 ind/m^2^) to secondary decomposers (770 ± 77 ind/m^2^). However, in each of the trophic guilds the abundance varied with forest type, and this depended on region (significant forest type × region interaction; Table 1). In primary decomposers at the sandy sites, the density was highest in beech and Douglas fir‐beech mixtures and lowest in Douglas fir and spruce monocultures, whereas at the loamy sites the density was lowest in pure spruce and beech‐spruce mixtures and similar in the other forest types (Figure 2a). Contrasting the pattern in primary decomposers, the abundance of secondary decomposers was generally highest in pure spruce and spruce‐beech mixtures than in the other forest types, with this being more pronounced at the sandy than at the loamy sites (Figure 2b). Similar to primary and secondary decomposers, the abundance of predators was generally higher at the sandy than at the loamy sites (Figure 2c), and similar to secondary decomposers, the abundance was low in Douglas fir and beech‐Douglas fir mixtures. However, at the sandy sites the abundance of predators was similarly high in beech, spruce and beech spruce mixtures, whereas at the loamy sites it was at similar low level in spruce and beech spruce mixtures and higher in beech and beech‐Douglas fir mixtures.

Species richness of the three trophic guilds generally differed much less between forest types and regions than abundance (Table 1, Figure 2d–f). The numbers were generally low with on average 1.5 ± 0.5 primary decomposer, 7.1 ± 2.0 secondary decomposer and 5.3 ± 1.3 predator species per sample. Only the richness of predators varied among forest types but this depended on region (significant forest type × region interaction; Table 1, Figure 2f). In beech, Douglas fir and Douglas fir‐beech mixture it was higher at the loamy than at the sandy sites, whereas in spruce and spruce‐beech mixtures it was lower at the loamy than at the sandy sites.

### 3.2 Community composition including trophic guilds

**FIGURE 3:**
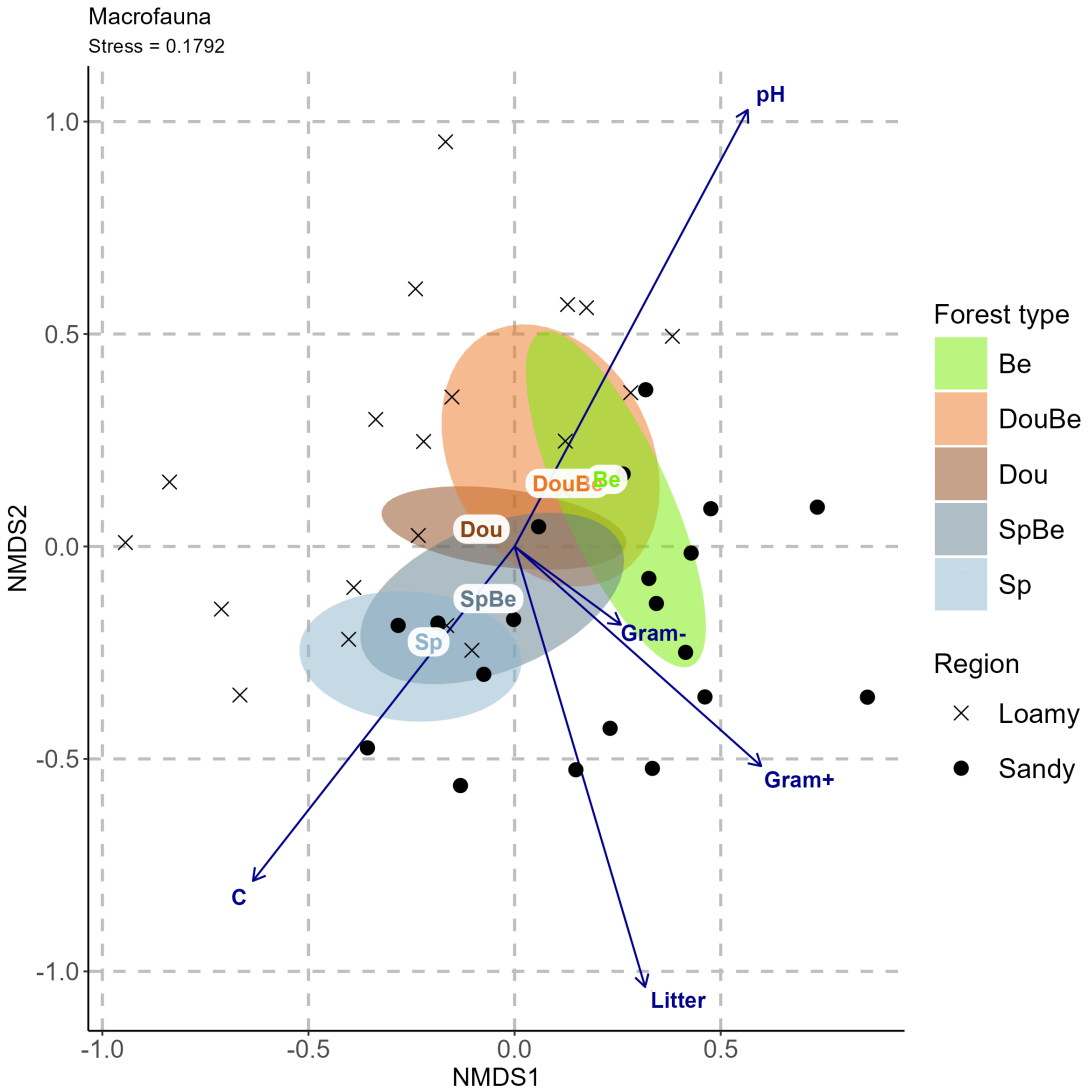
Nonmetric multidimensional scaling (NMDS) reflecting the relationship between macrofauna species in five forest types [Douglas fir (Dou), Douglas fir‐beech mixture (DouBe), beech (Be), spruce‐beech mixture (SpBe), spruce (Sp)] at two regions (sandy and loamy) and environmental factors including soil pH (pH), soil carbon (C), litter mass (Litter), Gram^+^ bacteria (Gram+) and Gram^−^ bacteria (Gram‐). Ellipes represent 95% confidence ranges for Be (green), DouBe (orange), Dou (brown), SpBe (dark blue) and Sp (light blue).

Macrofauna community composition differed significantly among forest types (PERMANOVA; *R* ^2^ = .173, *p* = .001) and between regions (PERMANOVA; *R* ^2^ = .105, p = .001), but the interaction was not significant. The first axis mainly sperated the two regions and the second axis mainly the forest types (Figure 3). Separation of the regions was closely associated with Gram^+^ bacteria being more abundant at sandy sites. Among forerst types spruce and beech differed most with Douglas fir being intermediate. Seperation of forest types was closely associated with soil pH and (in opposite direction) with soil carbon.

**FIGURE 4:**
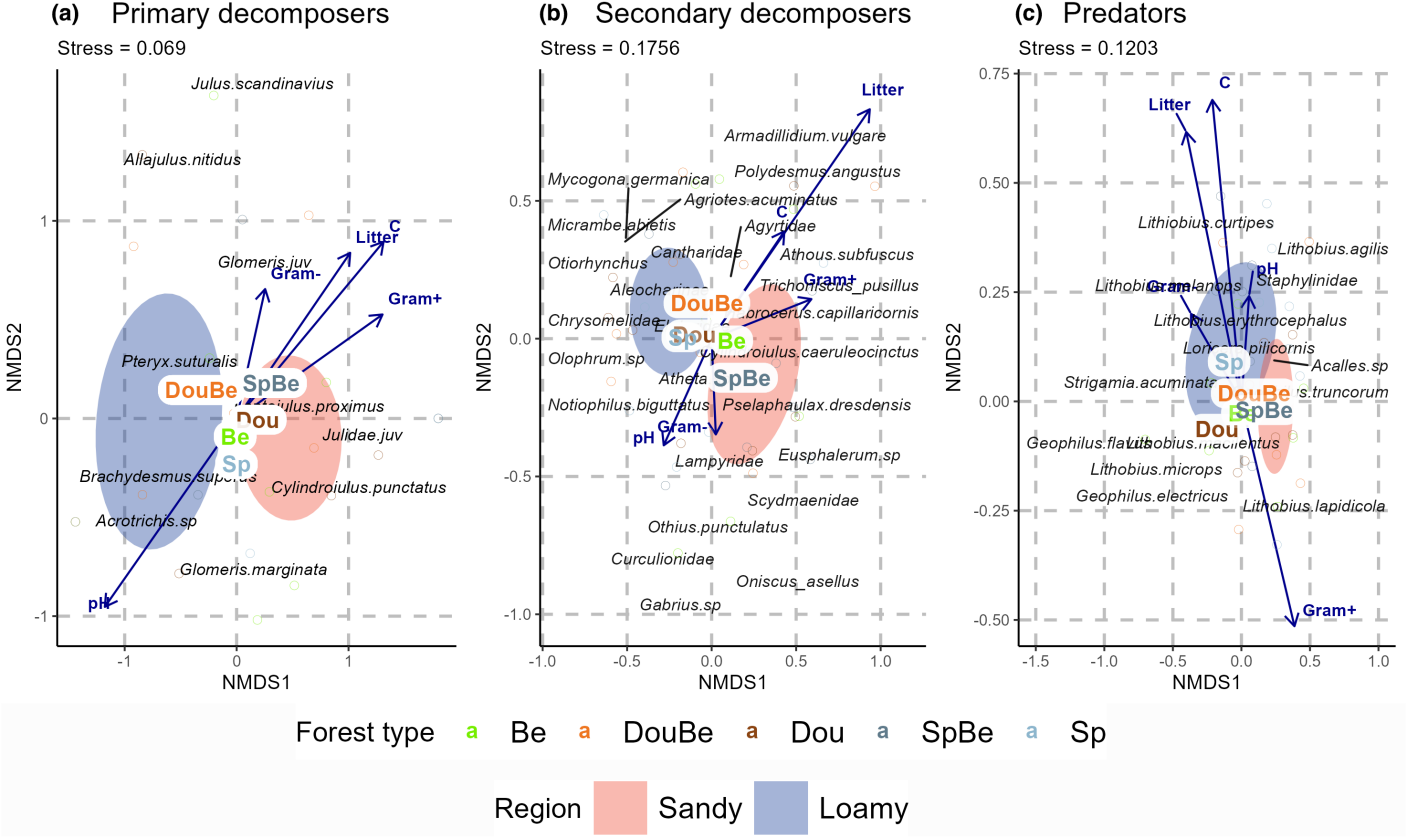
Nonmetric multidimensional scaling (NMDS) reflecting the relationship between (a) primary decomposers, (b) secondary decomposers and (c) predators in five forest types (Dou = Douglas fir, DouBe = Douglas fir‐ beech mixture, Be = beech, SpBe = spruce‐beech mixture and Sp = spruce). Environmental factors include soil pH (pH), soil carbon (C), litter mass (Litter), Gram^+^ bacteria (Gram+) and Gram^−^ bacteria (Gram‐). Ellipes represent 95% confidence ranges for Be (green), DouBe (orange), Dou (brown), SpBe (dark blue) and Sp (light blue).

Primary decomposer community composition differed significantly between regions (PERMANOVA; *R* ^2^ = .128, *p* = .001) but not between forest types (Figure 4a). The first axis separated the two regions and the second axis the forest types (although not significant). The communities at the sandy sites were associated with high proportion of Gram^+^ bacteria, whereas those at the loamy sites with high pH.

Secondary decomposer community composition significantly differed between both forest types and regions (PERMANOVA; *R* ^2^ = .213, *p* = .002 and *R* ^2^ = .08, *p* = .001, respectively). The communities at the sandy sites again were associated with high proportion of Gram^+^ bacteria and those at the loamy sites with high pH (Figure 4b). The second axis mainly separated Douglas fir‐beech from spruce‐beech forests with the former being associated with higher amounts of litter and the latter with higher pH and higher proportion of Gram^−^ bacteria.

Similar to primary decomposers, predator community composition significantly differed between regions (PERMANOVA; *R* ^2^ = .088, *p* = .001) but not among forest types (Figure 4c). Communities at the sandy sites again correlated with high proportion of Gram^+^ bacteria, whereas those at the loamy sites with a number of environmental factors including the amount of litter and soil carbon concentration.

### 3.3 Stable isotope metrics

**FIGURE 5:**
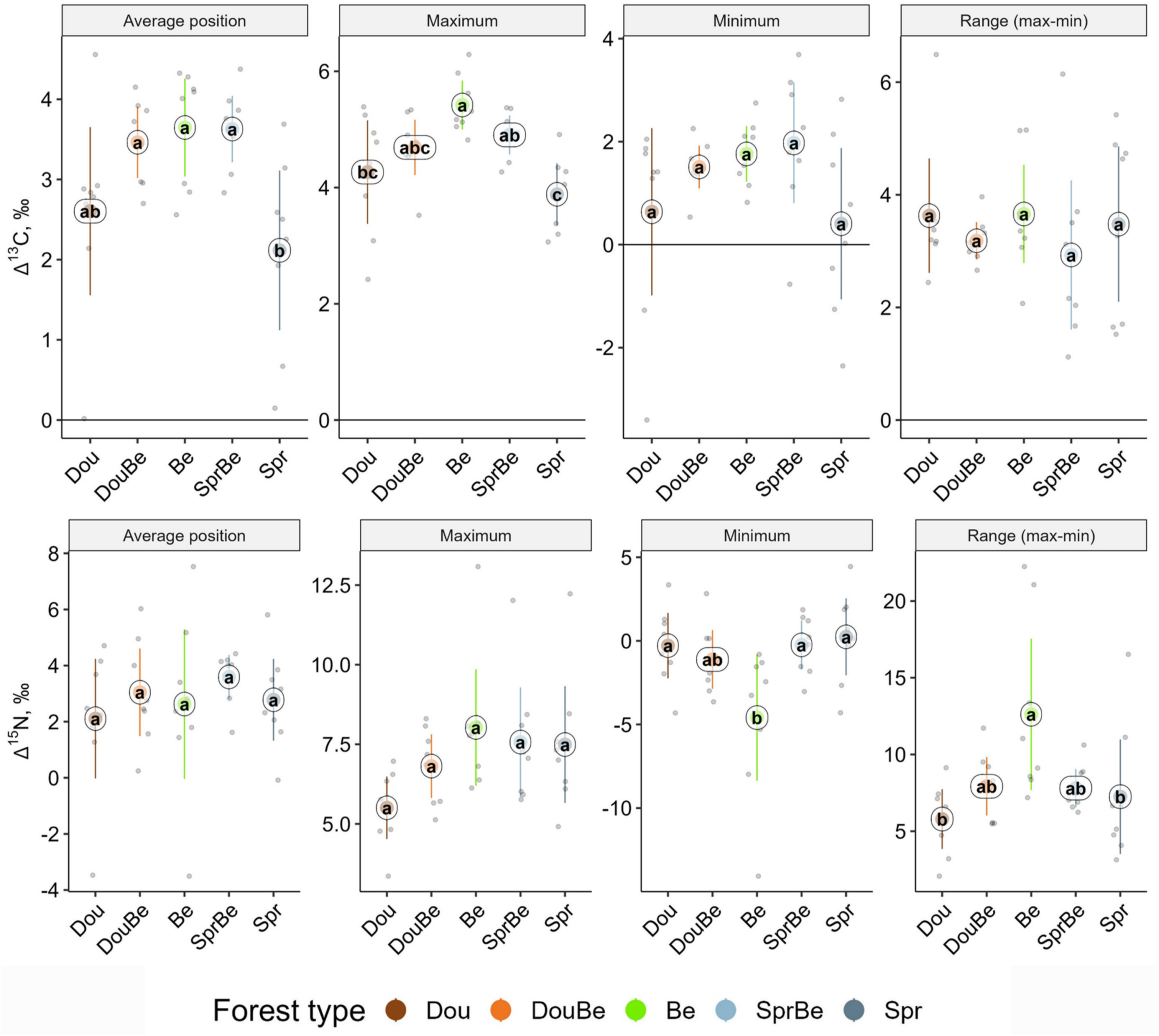
Average position, maximum, minimum, and range between minimum and maximum Δ^13^C (upper panel) and Δ^15^N (lower panel) values of total macrofauna in Douglas fir (Dou), Douglas fir‐beech (DouBe), beech (Be), spruce‐beech (SprBe) and spruce forests (Spr); means (circles), confidence intervals and individual measurements (small dots); means marked with different letters differ significantly (*p* < .05, Tukey's HSD test).

The Δ^13^C values of the total macrofauna communities were higher in beech forests than in conifer‐only forests, and this was due to similar shifts in both minimum and maximum values (*F*_4,34_ = 5.80, *p* = .001; Figure 5). In sandy sites, the Δ^13^C values were higher for average position (*F*_1,34_ = 5.77, *p* = .021). Minimum Δ^15^ *N* values of total macrofauna communities also differed between forest types with lowest values in beech and similarly high values in spruce and Douglas fir forests (*F*_4,34_ = 3.88, *p* = .011; Figure 5). By contrast, the range between minimum and maximum Δ^15^N values was largest in beech and lowest in spruce and Douglas fir forests (*F*_4,34_ = 4.0, *p* = .009). Multidimensional metrics did not differ significantly among forest types, but Isotopic Dispersion differed between regions (Table S2).

**FIGURE 6:**
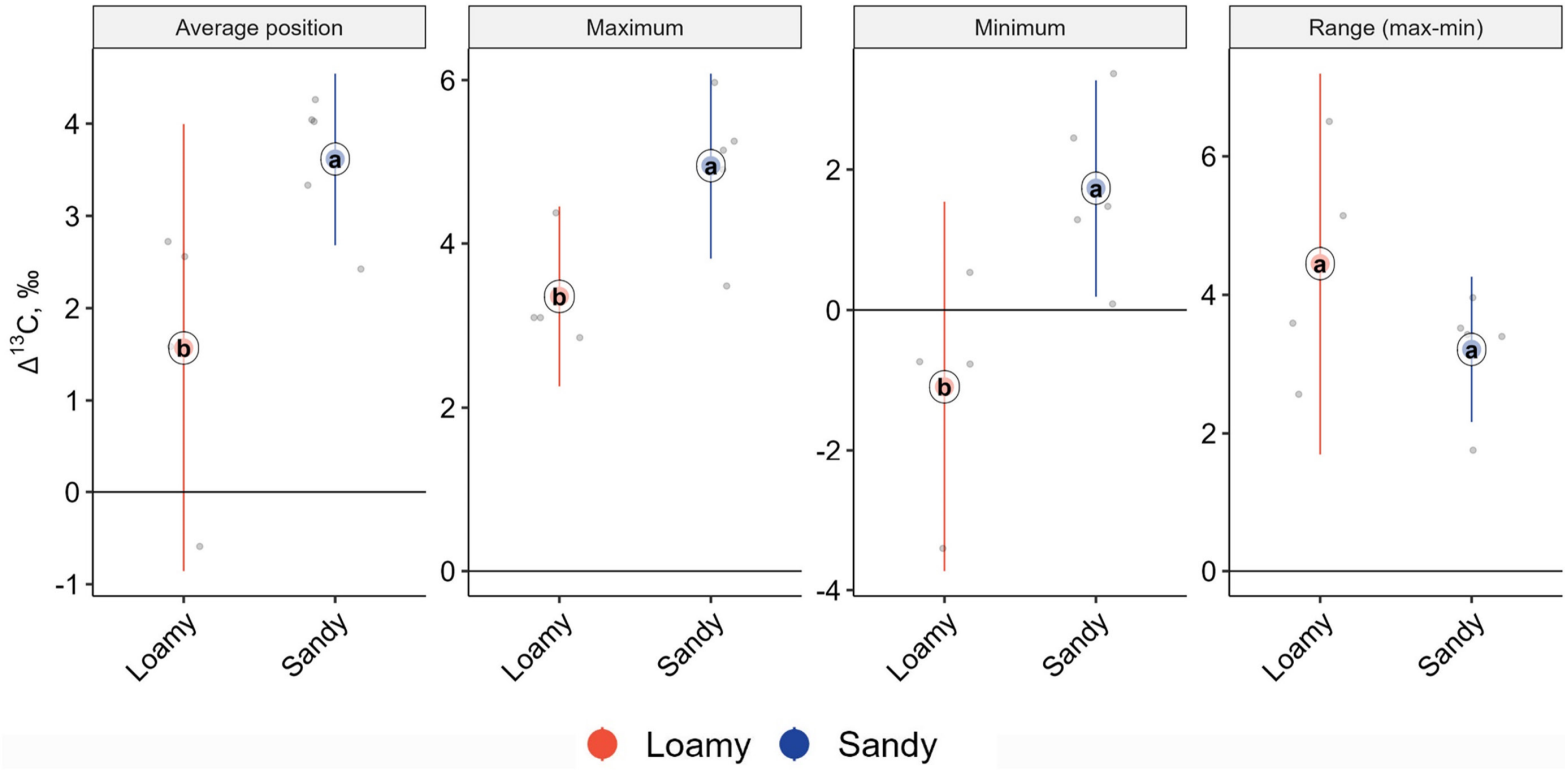
Average position, maximum, minimum and range between minimum and maximum Δ^13^C values of primary decomposers in loamy and sandy sites; means (circles), confidence intervals and individual measurements (small dots); means marked with different letters differ significantly (*p* < .05, Tukey's HSD test).

**FIGURE 7:**
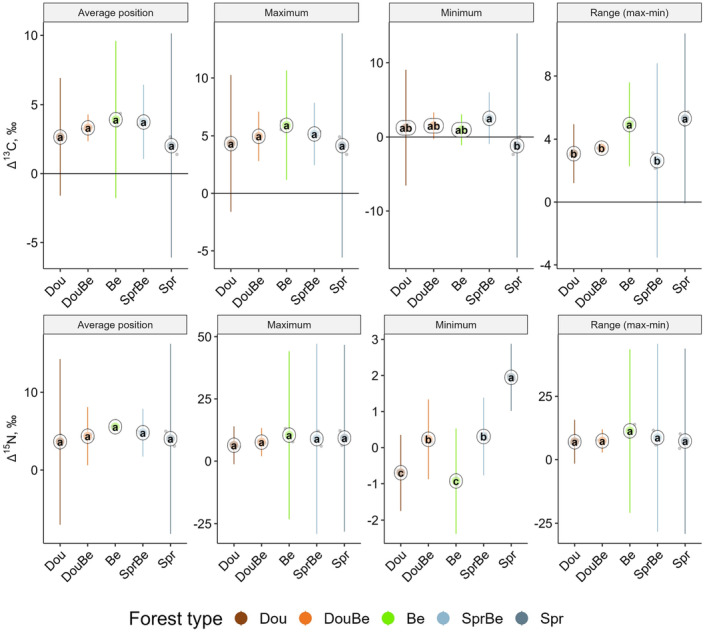
Average position, maximum, minimum, and range between minimum and maximum Δ^13^C (upper panel) and Δ^15^N (lower panel) values of predators in Douglas fir (Dou), Douglas fir‐beech (DouBe), beech (Be), spruce‐beech (SprBe) and spruce forests (Spr); means (circles) confidence intervals and individual measurements (small dots); means marked with different letters differ significantly (*p* < .05, Tukey's HSD test).

In primary decomposers, none of the Δ^13^C metrics (average position, maximum, minimum and range) differed significantly among forest types, but between regions with average position (*F*_1,4_ = 7.06, *p* = .033), maximum (*F*_1,4_ = 8.31, *p* = .024) and minimum (*F*_1,4_ = 8.63, *p* = .022) being 2.0, 1.6 and 2.8‰ higher at the sandy sites, respectively (Figure 6, Table S2). Secondary decomposers showed a similar pattern in Δ^13^C metrics, but neither differed significantly among forest types nor between regions (Table S2, Figure S1 and Data S2). Across forest types and regions, the average position, maximum, minimum and range were 2.7 ± 0.9‰, 4.9 ± 0.5‰, 0.8 ± 1.3‰, and 4.2 ± 1.1‰. In predators, minimum (*F*_5,5_ = 6.85, *p* = .045) and range (*F*_5,5_ = 31.63, *p* = .0028) of Δ^13^C values differed significantly between forest types but not between regions (Table S2); minimum values were highest in beech‐spruce mixture and lowest in spruce, and the range was largest in beech and spruce forests and smaller in the other forest types (Figure 7).

Δ^15^N values of primary and secondary decomposers generally did not differ significantly among forest types and between regions (Table S3); in primary decomposers across forest types and regions the average position, maximum, minimum and range were 0.6 ± 0.7‰, 3.3 ± 1.0‰, −2.6 ± 1.5‰ and 5.9 ± 1.9‰, respective values for secondary decomposers were 2.9 ± 1.5‰, 6.8 ± 1.3‰, −2.3 ± 3.7‰ and 9.2 ± 3.6‰. In predators, minimum Δ^15^N values were highest in spruce and lowest in Douglas fir and beech, with conifer‐beech mixtures being in between (ANOVA; *F*_5,5_ = 227.41, *p* = .0001; Figure 7). Across forest types and regions, the average position, maximum, minimum and range were 4.5 ± 0.8‰, 8.5 ± 2.9‰, 0.2 ± 1.1‰ and 8.4 ± 2.8‰ (Figure S2).

Multidimensional metrics of each of the three guilds neither differed significantly among forest types nor between regions (Table S3, Figure S3), with the exception of isotopic evenness in primary decomposers which was significant higher at sandy than loamy sites (*F*_1,4_ = 11.09, *p* = .013).

## 4 DISCUSSION

We investigated the response of three macrofauna trophic guilds, primary decomposers, secondary decomposers and predators, to different forest types including pure and mixed stands as well as native and introduced conifer species. Overall, differences in macrofauna abundance and species richness in the different forest types were guild specific, with only primary decomposers benefitting from Douglas fir compared to spruce forests. Secondary decomposers and predators reached maximum abundance in spruce forest and spruce‐beech mixtures especially at the sandy sites. Species richness of primary and secondary decomposers generally differed little between forest types, but at sandy sites species richness of predators was lowest in spruce forests and beech‐spruce mixtures. Communities of all three guilds differed between regions, but only the total macrofauna and secondary decomposer communities were influenced by forest type. Stable isotope analysis indicated limited effects of forest type on the trophic niches of macrofauna species of each of the trophic guilds, but strongly influenced Δ^13^C values and food‐chain length of the total macrofauna community.

### 4.1 Soil fauna guilds in different forest types and regions

Region, i.e. loamy and sandy sites, differed significantly in the abundance of trophic guilds but little in their richness, although both showed similar patterns. Typically, differences in abundance among forest types were more pronounced in the sandy than loamy sites, in line with our first hypothesis. At sandy sites, the abundance of primary decomposers in beech and Douglas fir‐beech mixed forests considerably exceeded that in spruce, spruce‐beech mixed and Douglas fir forests. As beech litter is of low food quality this is unlikely due to factors related to litter quality (Jacob et al., 2010; Thomas & Prescott, 2000) and may point to the importance of root‐derived resources. In fact, at sandy sites fine root biomass in beech forests has been shown to be larger than in coniferous forests (Lwila et al., 2021), and fine root biomass and production typically increase with nutrient deficiency (Lwila et al., 2023). In contrast to primary decomposers, secondary decomposers peaked in spruce, and at sandy sites also in beech‐spruce mixed forests, which may be related to the thick litter layer in spruce and associated supply of microbial resources. Previously, it has been shown that saprotrophic beetles prefer spruce to Douglas fir in particular at sandy sites (Gossner et al., 2016). The very low abundance of secondary decomposers in Douglas fir and Douglas fir‐beech forests may also be related to reduced soil microbial biomass associated with reduced root‐derived resources (Lu & Scheu, 2021; Lwila et al., 2021).

Typically, the abundance and species richness of total macrofauna and each of the three macrofauna trophic guilds in mixed stands was intermediate between pure coniferous and pure beech stands, supporting our second hypothesis for abundance but not species richness. This is consistent with earlier studies suggesting that mixed forests of beech and conifers increase habitat complexity and resource availability compared to conifer monocultures for a wide range of taxa including plants, fungi, ground beetles and springtails (Budde et al., 2011; Korboulewsky et al., 2021; Kriegel et al., 2021; Likulunga et al., 2021). Generally, however, differences in abundance between forest stands and also regions were considerably more pronounced than those between richness, as also reported for earthworms and mesofauna (Korboulewsky et al., 2016).

Primary decomposer abundance and richness was higher in Douglas fir than spruce forests, supporting our first and second hypotheses. Presumably, primary decomposers profit from higher food quality and the higher pH of Douglas fir compared to spruce needles (Pontégnie et al., 2005). At the sandy sites the abundance and richness of primary decomposers were highest in beech and Douglas fir‐beech mixtures indicating that potential negative effects of Douglas fir are offset by the presence of beech. Similarly, Engel (2001) found Isopoda and Diplopoda to benefit from Douglas fir compared to spruce, and David et al. (2023) found species richness of macrofauna detritivores to be increased in mixed conifer forests, supporting the positive influence of Douglas fir and its mixture with beech for primary decomposers.

In contrast to primary decomposers, the abundance of secondary decomposers was generally highest in spruce forests and similarly low in beech and Douglas fir forests. As most of the species comprised secondary decomposers, total macrofauna abundance was similar to secondary decomposers. Secondary decomposers comprised predominantly beetle larvae, in particular larvae of Elateridae and Aleocharinae, predominantly living as detritivores, but also consuming plant roots or living as predators (Samoylova & Tiunov, 2017; Wolters, 1989). Based on stable isotope analysis, we classified them as secondary decomposers and in our study they accounted for 40% of the individuals of this trophic guild. They typically reach high density in soils of low pH (Kula, 2014) and are found in high numbers in spruce forests (Schaefer, 1990; Scheu et al., 2003). This resembles the high density of secondary decomposer mesofauna, such as Collembola and Oribatida, in acidic forests with thick organic layers (Korboulewsky et al., 2016; Maraun & Scheu, 2000). Aleocharinae rove beetles favor microhabitats with high amounts of deadwood and thick humus layers (Irmler & Gürlich, 2007). Generally, larvae and adults of rove beetles live as predators and reach higher abundance in spruce than beech forests (Sührig & Schaefer, 2001) preying e.g., on microarthropods such as springtails. However, in part they also feed on fungi and this applies in particular to Aleocharinae (Irmler & Lipkow, 2018; Scheu & Falca, 2000). Although their abundance differed between forest types, species richness remained unaffected by forest type, this, however, needs to be treated with caution as larvae were only identified to subfamily level.

Predator abundance and richness were generally highest in beech forests and at the sandy sites also in spruce and beech‐spruce mixed forests. Predatory macroarthropods predominantly comprised centipedes, which typically reach high density in temperate forests and dominate the biomass of macrofauna predators (Scheu et al., 2003). A number of centipede species reach high abundance in beech dominated forests feeding on a wide spectrum of prey (Bonato et al., 2021; Ferlian et al., 2012; Potapov et al., 2022). Adult and larvae of rove beetles also contributed to the high density of macrofauna predators. Both presumably feed heavily on secondary decomposer mesofauna such as Collembola (Günther et al., 2014; Hartmann, 1979), which reach high densities in spruce and beech forests (Kohlert & Roth, 2000; Salamon et al., 2008).

### 4.2 Community composition

Contrasting the strong difference in total macrofauna community composition among forest types, the community composition of the three macrofauna trophic guilds mainly differed between regions but little among forest types, partly rejecting our third hypothesis. Regional environmental factors have also been shown to be more important than forest type in shaping soil mesofauna communities (Erdmann et al., 2012). NMDS ordination identified soil pH and soil organic carbon as important factors structuring the community composition of total as well as each of the three trophic guilds of macrofauna, with the separation of forest types being most pronounced in total macrofauna communities, which is similar to earlier studies (Pollierer et al., 2021). Strong separation of beech and spruce forests along pH and soil organic carbon gradients reflects soil acidification as well as litter accumulation in spruce stands, with the intermediate position of Douglas fir likely reflecting lower adidification than in spruce forests and generally more palatable litter compared to spruce and beech (Kupka et al., 2013). In addition to abiotic factors, microbial community composition, in particular Gram^+^ bacteria, were identified as important structuring force of total as well as each of the three trophic guilds of macrofauna. Compared to Gram^−^ bacteria, Gram^+^ bacteria more heavily depend on complex litter compounds (Fanin et al., 2019; Kramer & Gleixner, 2008) and their role in separating macrofauna trophic guilds between sandy and loamy sites may reflect that litter resources play a more important role in nourishing macrofauna communities at the loamy than at the sandy sites, similar to what has been observed in Collembola along a mountain gradient (Lux et al., 2024a, 2024b). The fact that the community structure of secondary decomposers also differed among forest types, mostly between Douglas fir‐beech and spruce‐beech mixtures, may also be related to differences in microbial community structure, in particular Gram^+^ bacteria being associated with Douglas fir‐beech mixed forests, likely again reflecting increased litter quality in Douglas fir‐beech mixed forests compared to beech forests, as also indicated by higher abundance of primary decomposers. However, also other factors such as canopy openness not considered in our study may contribute to differences in macrofauna community structure between forest types and this needs closer attention in future studies.

### 4.3 Trophic structure

Generally, the trophic structure of the three trophic guilds of macrofauna varied mostly between coniferous and beech/mixed forest with little difference among forest types and between regions in the three trophic guilds, pointing to the resistance of the trophic structure of soil food webs to variations in environmental factors. The consistency of the average trophic position of each of the three trophic guilds across forest types, as indicated by Δ^15^N values, supports our fourth hypothesis. Total macrofauna community had lower minimum Δ^15^N values in beech and Douglas fir‐beech mixed forests, likely resulting from comprising more primary decomposers with low Δ^15^N values. Presumably, this was also responsible for the wider range of Δ^15^N values in beech compared to conifer forests. Among macrofauna guilds, only minimum Δ^15^N values of predators were significantly higher in spruce forests than in the other forest types. Potentially, predators in spruce forest feed less on low trophic level prey such as primary decomposers than in other forest types (Ferlian & Scheu, 2014; Scheu & Falca, 2000), which likely is related to low food quality of spruce needles resulting in low abundance of primary decomposers, whereas secondary decomposers such as Collembola are thriving in spruce forests (Korboulewsky et al., 2021).

The detrital shift in δ^13^C values, which has been identified as a typical feature of soil food webs, also applied to the studied macrofauna trophic guilds and averaged 3.2‰, which is well in the range reported previously (Potapov et al., 2019). The lower shift in δ^13^C values in total macrofauna in coniferous compared to beech forests may reflect the low quality of beech litter (Klarner et al., 2014). For macrofauna guilds, however, only in predators minimum and range of Δ^13^C values differed among forest types, with in particular spruce and beech‐spruce mixed forests differing from the other forest types. Potentially, this again is related to the thick leaf litter layer in spruce and beech‐spruce mixed forests restricting the access to prey deeper in soil relying on root‐derived resources (Ferlian & Scheu, 2014; Günther et al., 2014). The lower average, maximum and minimum Δ^13^C values of total macrofauna and primary decomposers at the loamy than the sand sites may point to increased consumption of more nutrient rich and less decomposed litter resources at the former sites.

## 5 CONCLUSIONS

Overall, soil macrofauna trophic guilds and communities varied more between regions than among forest types, with differences between forest types being most prominent in total macrofauna communities. However, in particular primary decomposers benefited from non‐native Douglas fir, whereas the opposite was true for secondary decomposers. Presumably, this reflects higher food quality of Douglas fir compared to spruce leaf litter favoring primary decomposers, and poor litter quality of spruce litter resulting in a shift toward secondary decomposers feeding on microorganisms in spruce forests. The influence of mixed forests on soil macrofauna abundance and species richness typically was intermediate between the respective pure stands suggesting that mixed forests may buffer potential detrimental effects of pure stands of both native and non‐native conifers. However, in particular at sandy sites Douglas fir detrimentally affected macrofauna trophic guilds pointing to the importance of regional factors. The strong influence of regional factors was also reflected in the distinct difference in the structure of macrofauna trophic guilds between sandy and loamy sites markedly exceeding the influence of forest types. Differences in Δ^13^C values of total macrofauna communities in different forest types point to the adaptation of soil macrofauna food webs to differences in resource quality and environmental factors between deciduous and conifer forest. Lower minimum Δ^15^N values in beech than conifer forests support this conclusion. Significant changes in the trophic position of macrofauna predators with forest type point to predators as being most sensitive to environmental changes. Overall, the results indicate that mixed forests of beech and conifers may buffer potential detrimental effects of monocultures of conifers with this also applying to non‐native Douglas fir.

## AUTHOR CONTRIBUTIONS

**Ronja Wenglein:** Conceptualization (equal); data curation (equal); formal analysis (lead); investigation (lead); methodology (equal); project administration (supporting); software (equal); validation (equal); visualization (lead); writing – original draft (lead); writing – review and editing (equal). **Jing‐Zhong Lu:** Conceptualization (equal); data curation (equal); formal analysis (supporting); investigation (equal); writing – review and editing (equal). **Stefan Scheu:** Conceptualization (supporting); data curation (supporting); formal analysis (equal); funding acquisition (lead); investigation (supporting); methodology (lead); project administration (lead); supervision (lead); visualization (supporting); writing – original draft (supporting); writing – review and editing (equal).

## CONFLICT OF INTEREST STATEMENT

The authors declare that they have no known competing financial interests or personal relationships that could have appeared to influence the work reported in this paper.

## Supporting information


Data S1.

Data S2.

## Data Availability

Data and codes used in the study are available in the Supplementary Material.
