# Supplementary material for: The influence of forest types including native and non‐native tree species on soil macrofauna depends on site conditions
Source: Ecol Evol. 2024 Sep 18;14(9):e70311. doi: 10.1002/ece3.70311 (PMC11410562; doi:10.1002/ece3.70311)
Supplement: Supplementary file 1 — Data S1. [file ECE3-14-e70311-s002.zip]

**Appendix**

**Table S1**: List of species including higher order taxonomy and trophic guilds assigned by literature or Δ^15^N values from this study.

| Species Name | Assigned Guild | Class/Order | Family | Level of Identification | Reference for Guild |
| --- | --- | --- | --- | --- | --- |
| *Acalyptus carpini* (Fabricius, 1792) | primary decomposer | Coleoptera | Curculionidae | Species | (Anderson, 2012) |
| Acrotrichis (Motschulsky, 1848) | primary decomposer | Coleoptera | Ptiliidae | Genus | Δ^15^N |
| *Ampedus (Ampedus) balteatus* (Linnaeus, 1758) | primary decomposer | Coleoptera | Elateridae | Species | (Nieto et al. 2010) |
| Cerambycidae (larvae) | primary decomposer | Coleoptera | Cerambycidae | Family | (Linsley, 1959) |
| *Ectinus aterrimus* (Linnaeus, 1761) | primary decomposer | Coleoptera | Elateridae | Species | (Pollierer et al. 2021) |
| Habrocerus (Erichson, 1839) | primary decomposer | Coleoptera | Staphylinidae | Genus | Δ^15^N |
| Lordithon (Thomson, 1859) | primary decomposer | Coleoptera | Staphylinidae | Genus | Δ^15^N |
| *Oxystoma craccae* (Linnaeus, 1767) | primary decomposer | Coleoptera | Apionidae | Species | (Karolewski et al. 2020) |
| *Pocadius ferrugineus* (Fabricius, 1775) | primary decomposer | Coleoptera | Nitidulidae | Species | (Bouget et al. 2012) |
| *Pteryx suturalis* (Heer, 1841) | primary decomposer | Coleoptera | Ptiliidae | Species | Δ^15^N |
| *Allajulus nitidus* (Verhoeff, 1891) | primary decomposer | Diplopoda | Julidae | Species | Δ^15^N |
| *Brachydesmus superus* (Latzel, 1884) | primary decomposer | Diplopoda | Polydesmidae | Species | Δ^15^N |
| *Craspedosoma rawlinsi*(Leach, 1814) | primary decomposer | Diplopoda | Craspedosomatidae | Species | (Spelda, 1999) |
| *Cylindroiulus latestriatus* (Curtis, 1845) | primary decomposer | Diplopoda | Julidae | Species | (Stašiov et al. 2021) |
| *Cylindroiulus punctatus* (Leach, 1815) | primary decomposer | Diplopoda | Julidae | Species | Δ^15^N |
| Glomeris | primary decomposer | Diplopoda | Glomeridae | Species | (Pollierer et al. 2009) |
| *Glomeris klugii* (Brandt, 1833) | primary decomposer | Diplopoda | Glomeridae | Species | (Scheu and Falca 2000) |
| *Glomeris marginata* (Villers, 1789) | primary decomposer | Diplopoda | Glomeridae | Species | Δ^15^N |
| Julidae | primary decomposer | Diplopoda | Julidae | Species | (Pollierer et al. 2021) |
| *Julus scandinavius* (Latzel, 1884) | primary decomposer | Diplopoda | Julidae | Species | Δ^15^N |
| *Leptoiulus proximus*(Nemec, 1896) | primary decomposer | Diplopoda | Julidae | Species | Δ^15^N |
| *Ommatoiulus sabulosus* (Linnaeus, 1758) | primary decomposer | Diplopoda | Julidae | Species | (Stašiov et al. 2021) |
| *Proteroiulus fuscus* (Am Stein, 1857) | primary decomposer | Diplopoda | Blaniulidae | Species | (Blower, 1985) |
| *Agriotes (Agriotes) acuminatus* (Stephens, 1830) | secondary decomposer | Coleoptera | Elateridae | Species | Δ^15^N |
| Agyrtidae (larvae) | secondary decomposer | Coleoptera | Agyrtidae | Family | Δ^15^N |
| Aleocharinae (larvae) | secondary decomposer | Coleoptera | Staphylinidae | Subfamily | Δ^15^N |
| *Ampedus (Ampedus) rufipennis* (Stephens, 1830) | secondary decomposer | Coleoptera | Elateridae | Species | Δ^15^N |
| Atheta (Thomson, 1858) | secondary decomposer | Coleoptera | Staphylinidae | Genus | Δ^15^N |
| *Athous (Haplathous) subfuscus* (O. F. Muller, 1764) | secondary decomposer | Coleoptera | Elateridae | Species | Δ^15^N |
| Cantharidae (larvae) | secondary decomposer | Coleoptera | Cantharidae | Family | Δ^15^N |
| Chrysomelidae (larvae) | secondary decomposer | Coleoptera | Chrysomelidae | Family | Δ^15^N |
| Ciidae (larvae) | secondary decomposer | Coleoptera | Ciidae | Family | (Gimmel and Ferro, 2018) |
| *Clambus punctulum* (Beck, 1817) | secondary decomposer | Coleoptera | Clambidae | Species | (Lawrence and Newton, 1980) |
| Coprophilus (Latreille, 1829) | secondary decomposer | Coleoptera | Staphylinidae | Genus | (Heinrich et al. 1991) |
| Curculionidae (larvae) | secondary decomposer | Coleoptera | Curculionidae | Family | Δ^15^N |
| *Dasycerus sulcatus* (Brongniart, 1800) | secondary decomposer | Coleoptera | Staphylinidae | Species | (Hans 1991) |
| Dermestidae (larvae) | secondary decomposer | Coleoptera | Dermestidae | Family | (Zhantiev, 2008) |
| Elateridae (larvae) | secondary decomposer | Coleoptera | Elateridae | Family | Δ^15^N |
| Eusphalerum (Kraatz, 1857) | secondary decomposer | Coleoptera | Staphylinidae | Genus | Δ^15^N |
| Gabrius (Stephens, 1829) | secondary decomposer | Coleoptera | Staphylinidae | Genus | Δ^15^N |
| *Habrocerus capillaricornis* (Gravenhorst, 1806) | secondary decomposer | Coleoptera | Staphylinidae | Species | Δ^15^N |
| *Hylurgops palliatus* (Gyllenhal, 1813) | secondary decomposer | Coleoptera | Curculionidae | Species | (Borkowski & Skrezecz, 2015) |
| *Lamprinodes saginatus* (Gravenhorst, 1806) | secondary decomposer | Coleoptera | Staphylinidae | Species | (Byrd & Castner, 2011) |
| Lampyridae (larvae) | secondary decomposer | Coleoptera | Lampyridae | Family | Δ^15^N |
| *Latridius minutus* (Linnaeus, 1767) | secondary decomposer | Coleoptera | Latridiidae | Species | (Lord et al. 2010) |
| Lycidae (larvae) | secondary decomposer | Coleoptera | Lycidae | Family | (Motyka et al. 2017) |
| *Micrambe abietis*(Paykull, 1798) | secondary decomposer | Coleoptera | Cryptophagidae | Species | Δ^15^N |
| *Notiophilus biguttatus* (Fabricius, 1779) | secondary decomposer | Coleoptera | Carabidae | Species | Δ^15^N |
| Olophrum (Erichson, 1839) | secondary decomposer | Coleoptera | Staphylinidae | Genus | Δ^15^N |
| *Othius punctulatus* (Goeze, 1777) | secondary decomposer | Coleoptera | Staphylinidae | Species | Δ^15^N |
| Otiorhynchus (Germar, 1822) | secondary decomposer | Coleoptera | Curculionidae | Genus | Δ^15^N |
| *Oxytelus (Tanycraerus) laqueatus* (Marsham, 1802) | secondary decomposer | Coleoptera | Staphylinidae | Species | (Byrd & Castner, 2011) |
| *Pella laticollis* (Märkel, 1842) | secondary decomposer | Coleoptera | Staphylinidae | Species | Δ^15^N |
| *Pselaphaulax dresdensis* (Herbst, 1792) | secondary decomposer | Coleoptera | Staphylinidae | Species | Δ^15^N |
| Pselaphinae (larvae) | secondary decomposer | Coleoptera | Staphylinidae | Subfamily | (Irmler et al. 2018) |
| Raphirus | secondary decomposer | Coleoptera | Staphylinidae | Genus | Δ^15^N |
| Rhysodinae (larvae) | secondary decomposer | Coleoptera | Carabidae | Subfamily | (Arnett et al. 2002) |
| Scaphidiinae (larvae) | secondary decomposer | Coleoptera | Staphylinidae | Subfamily | (Irmler et al. 2018) |
| Scydmaenidae (larvae) | secondary decomposer | Coleoptera | Scydmaenidae | Family | Δ^15^N |
| Steninae (larvae) | secondary decomposer | Coleoptera | Staphylinidae | Subfamily | (Irmler et al. 2018) |
| *Telmatophilus brevicollis* (Aube, 1862) | secondary decomposer | Coleoptera | Cryptophagidae | Species | (Otero , 2010) |
| *Trypodendron lineatum* (Olivier, 1795) | secondary decomposer | Coleoptera | Curculionidae | Species | (Lehenberger et al. 2018) |
| *Cylindroiulus caeruleocinctus* (Wood, 1864) | secondary decomposer | Diplopoda | Julidae | Species | Δ^15^N |
| *Mycogona germanica* (Verhoeff, 1892) | secondary decomposer | Diplopoda | Chordeumatidae | Species | Δ^15^N |
| *Polydesmus angustus* (Latzel, 1884) | secondary decomposer | Diplopoda | Polydesmidae | Species | Δ^15^N |
| *Armadillidium vulgare* (Latreille, 1804) | secondary decomposer | Isopoda | Armadillidiidae | Species | Δ^15^N |
| *Ligidium hypnorum* (Cuvier, 1792) | secondary decomposer | Isopoda | Ligiidae | Species | (Scheu and Falca 2000) |
| *Oniscus asellus* (Linnaeus, 1758) | secondary decomposer | Isopoda | Oniscidae | Species | (Gunnarsson & Tunlid, 1986) |
| *Philoscia affinis* (Verhoeff, 1933) | secondary decomposer | Isopoda | Philosciidae | Species | (Schmidt, 2008) |
| *Porcellium conspersum* (Koch, 1841) | secondary decomposer | Isopoda | Trachelipodidae | Species | (Scheu and Falca 2000) |
| *Trichoniscus pusillus* (Brandt, 1833) | secondary decomposer | Isopoda | Trichoniscidae | Species | Δ^15^N |
| *Cryptops hortensis* (Donovan, 1810) | predator | Chilopoda | Cryptopidae | Species | (Voigtländer, 2011) |
| *Geophilus electricus* (Linnaeus, 1758) | predator | Chilopoda | Geophilidae | Species | (Voigtländer, 2011) |
| *Geophilus flavus* (De Geer, 1778) | predator | Chilopoda | Geophilidae | Species | Δ^15^N |
| *Geophilus insculptus* (Attems, 1895) | predator | Chilopoda | Geophilidae | Species | (Voigtländer, 2011) |
| *Geophilus truncorum* (Bergsoe & Meinert, 1866) | predator | Chilopoda | Geophilidae | Species | (Voigtländer, 2011) |
| *Geophilus truncorum* (Bergsoe & Meinert, 1866) | predator | Chilopoda | Geophilidae | Species | (Voigtländer, 2011) |
| Lithobius | predator | Chilopoda | Lithobiidae | Species | (Voigtländer, 2011) |
| *Lithobius (Lithobius) agilis* (C.L. Koch, 1847) | predator | Chilopoda | Lithobiidae | Species | (Pollierer et al. 2009) |
| *Lithobius (Lithobius) borealis* (Meinert, 1868) | predator | Chilopoda | Lithobiidae | Species | (Voigtländer, 2011) |
| *Lithobius (Lithobius) calcaratus* (C.L. Koch, 1844) | predator | Chilopoda | Lithobiidae | Species | (Voigtländer, 2011) |
| *Lithobius (Lithobius) erythrocephalus* (C.L. Koch, 1847) | predator | Chilopoda | Lithobiidae | Species | (Voigtländer, 2011) |
| *Lithobius (Lithobius) forficatus* (Linnaeus, 1758) | predator | Chilopoda | Lithobiidae | Species | (Voigtländer, 2011) |
| *Lithobius (Lithobius) lapidicola* (Meinert, 1872) | predator | Chilopoda | Lithobiidae | Species | (Voigtländer, 2011) |
| *Lithobius (Lithobius) macilentus* (L. Koch, 1862) | predator | Chilopoda | Lithobiidae | Species | (Voigtländer, 2011) |
| *Lithobius (Lithobius) melanops* (Newport, 1845) | predator | Chilopoda | Lithobiidae | Species | (Voigtländer, 2011) |
| *Lithobius (Lithobius) mutabilis* (L. Koch, 1862) | predator | Chilopoda | Lithobiidae | Species | (Ferlian et al. 2012) |
| *Lithobius (Lithobius) muticus* (C.L. Koch, 1847) | predator | Chilopoda | Lithobiidae | Species | (Voigtländer, 2011) |
| *Lithobius (Lithobius) subtilis* (Latzel, 1880) | predator | Chilopoda | Lithobiidae | Species | (Voigtländer, 2011) |
| *Lithobius (Monotarsobius) aeruginosus* (L. Koch, 1862) | predator | Chilopoda | Lithobiidae | Species | (Voigtländer, 2011) |
| *Lithobius (Monotarsobius) crassipes* (L. Koch, 1862) | predator | Chilopoda | Lithobiidae | Species | Δ^15^N |
| *Lithobius (Monotarsobius) curtipes* (C.L. Koch, 1847) | predator | Chilopoda | Lithobiidae | Species | (Eason 1964) |
| *Lithobius (Sigibius) microps* (Meinert, 1868) | predator | Chilopoda | Lithobiidae | Species | Δ^15^N |
| *Schendyla nemorensis* (C.L.Koch, 1837) | predator | Chilopoda | Schendylidae | Species | (Pollierer et al. 2009) |
| *Strigamia acuminata* (Leach, 1815) | predator | Chilopoda | Linotaeniidae | Species | Δ^15^N |
| *Strigamia crassipes* (C.L.Koch, 1835) | predator | Chilopoda | Linotaeniidae | Species | (Voigtländer, 2011) |
| Acalles (Schoenherr, 1825) | predator | Coleoptera | Curculionidae | Species | Δ^15^N |
| *Adalia (Adaliomorpha) conglomerata* (Linnaeus, 1758) | predator | Coleoptera | Coccinellidae | Species | (Hagen 1962) |
| *Anotylus rugosus* (Fabricius, 1775) | predator | Coleoptera | Staphylinidae | Species | (Steenberg et al. 1995) |
| Astenus (Dejean, 1833) | predator | Coleoptera | Staphylinidae | Genus | (Faly et al. 2017) |
| *Atrecus affinis* (Paykull, 1789) | predator | Coleoptera | Staphylinidae | Species | Δ^15^N |
| Bisnius (Stephens, 1829) | predator | Coleoptera | Staphylinidae | Genus | Δ^15^N |
| *Bryaxis puncticollis* (Denny, 1825) | predator | Coleoptera | Staphylinidae | Species | (Schomann et al. 2008) |
| Bythinus (Leach, 1817) | predator | Coleoptera | Staphylinidae | Genus | (Pollierer et al. 2021) |
| Carabidae | predator | Coleoptera | Carabidae | Family | Δ^15^N |
| Cleridae | predator | Coleoptera | Cleridae | Family | Δ^15^N |
| *Dalopius marginatus* (Linnaeus, 1758) | predator | Coleoptera | Elateridae | Genus | Δ^15^N |
| *Heterothops dissimilis* (Gravenhorst, 1802) | predator | Coleoptera | Staphylinidae | Species | Δ^15^N |
| *Loricera pilicornis* (Fabricius, 1775) | predator | Coleoptera | Carabidae | Species | Δ^15^N |
| *Masoreus wetterhallii* (Gyllenhal, 1813) | predator | Coleoptera | Carabidae | Species | (den Boer & Van Dijk, 1994) |
| Microsaurus | predator | Coleoptera | Staphylinidae | Genus | Δ^15^N |
| Neobisnius (Ganglbauer, 1895) | predator | Coleoptera | Staphylinidae | Genus | Δ^15^N |
| Ochthephilus (Mulsant & Rey, 1856) | predator | Coleoptera | Staphylinidae | Genus | (Arnett et al. 2002) |
| Ocypus (Leach, 1819) | predator | Coleoptera | Staphylinidae | Genus | (Orth et al. 1975) |
| *Philonthus (Philonthus) splendens* (Fabricius, 1793) | predator | Coleoptera | Staphylinidae | Species | (Pollierer et al. 2021) |
| Phymatura | predator | Coleoptera | Staphylinidae | Genus | (Arnett et al. 2002) |
| *Poecilus (Poecilus) cupreus* (Linne, 1758) | predator | Coleoptera | Carabidae | Species | (Kegel, 1994) |
| *Pselaphus heisei* (Herbst, 1792) | predator | Coleoptera | Staphylinidae | Species | Δ^15^N |
| Ptenidium (Erichson, 1845) | predator | Coleoptera | Ptiliidae | Genus | Δ^15^N |
| Pterostichus (Bonelli, 1810) | predator | Coleoptera | Carabidae | Genus | (Pollierer et al. 2021) |
| Scarabaeidae | predator | Coleoptera | Scarabaeidae | Family | Δ^15^N |
| Staphylinidae (Latreille, 1804) | predator | Coleoptera | Staphylinidae | Family | Δ^15^N |
| Stenichnus (Thomson, 1859) | predator | Coleoptera | Scydmaenidae | Genus | Δ^15^N |
| Strophosoma (Billberg, 1820) | predator | Coleoptera | Curculionidae | Genus | Δ^15^N |
| *Tachyporus dispar* (Paykull, 1789) | predator | Coleoptera | Staphylinidae | Species | (Dennis et al. 1994) |
| Tasgius (Stephens, 1829) | predator | Coleoptera | Staphylinidae | Genus | (Smetana 1965) |
| *Xantholinus (Xantholinus) linearis* (Olivier, 1795) | predator | Coleoptera | Staphylinidae | Species | (Scheu and Falca 2000) |
| *Xylostiba monilicornis* (Gyllenhal, 1810) | predator | Coleoptera | Staphylinidae | Species | (Ganglbauer, 1895) |

**Table S2:** PERMANOVA results on the Bray-Curtis Community matrix showing the effect of forest type, region and its interaction on community composition of the three trophic guilds (primary decomposers, secondary decomposers, predators). Significant effects are marked in bold; df, degrees of freedom; SS, sum of sqares.

|  | Total Macrofauna | | | | |
| --- | --- | --- | --- | --- | --- |
|  | Df | SumOfSqs | R2 | F-values | p-values |
| forest | **4** | **1.50** | **0.17** | **2.04** | **0.003** |
| **region** | **1** | **0.91** | **0.10** | **4.94** | **0.001** |
| forest x region | 4 | 0.76 | 0.09 | 1.04 | 0.434 |
| Residual | 30 | 5.52 | 0.63 |  |  |
| Total | 24 | 9.27 | 1.00 |  |  |
|  |  |  |  |  |  |
|  | Primary decomposers | | | | |
|  | Df | SumOfSqs | R2 | F-values | p-values |
| forest | 4 | 1.35 | 0.15 | 0.96 | 0.521 |
| **region** | **1** | **1.19** | **0.13** | **3.38** | **0.001** |
| forest x region | 4 | 1.46 | 0.16 | 1.03 | 0.430 |
| Residual | 15 | 5.28 | 0.57 |  |  |
| Total | 24 | 9.27 | 1.00 |  |  |
|  |  |  |  |  |  |
|  | Secondary decomposers | | | | |
|  | Df | SumOfSqs | R2 | F-values | p-values |
| **forest** | **4** | **1.52** | **0.21** | **2.62** | **0.001** |
| **region** | **1** | **0.57** | **0.08** | **3.93** | **0.002** |
| forest x region | 4 | 0.69 | 0.10 | 1.19 | 0.257 |
| Residual | 30 | 4.35 | 0.61 |  |  |
| Total | 39 | 7.12 | 1.00 |  |  |
|  |  |  |  |  |  |
|  | Predators | | | | |
|  | Df | SumOfSqs | R2 | F-values | p-values |
| **forest** | **4** | **1.57** | **0.13** | **1.32** | **0.092** |
| **region** | **1** | **1.07** | **0.09** | **3.62** | **0.002** |
| forest x region | 4 | 0.70 | 0.06 | 0.59 | 0.984 |
| Residual | 30 | 8.91 | 0.73 |  |  |
| Total | 39 | 12.26 | 1.00 |  |  |

**Table S3**: ANOVA F- and p-values on the effect of forest type, region on isotopic metrics of macrofauna and the three trophic guilds (primary decomposers, secondary decomposers, predators). Isotopic metrics include Average position (IPos), maximum (max), minimum (min), and range (range) between minimum and maximum, Isotopic Divergence (IDiv), Isotopic Dispersion (IDis), Isotpic Evenness (IEve), Isotopic Uniqueness (IUni) and Isotopic Richness (IRic) of Δ^13^C and Δ^15^N values of primary decomposers, secondary decomposers and predators. Significant factors are indicated in bold; df, degrees of freedom; SS, sum of sqares.

| Total macrofauna | |  |  |  |  |  |
| --- | --- | --- | --- | --- | --- | --- |
|  |  | df | SS | MS | F-value | p-value |
| min_d13C | Forest | 4 | 0.17 | 0.04 | 2.14 | 0.097 |
|  | Region | 1 | 0.05 | 0.05 | 2.28 | 0.139 |
|  | Residuals | 34 | 0.66 | 0.02 |  |  |
| min_d15N | **Forest** | **4** | **0.17** | **0.04** | **3.88** | **0.011** |
|  | Region | 1 | 0.02 | 0.02 | 1.70 | 0.200 |
|  | Residuals | 34 | 0.36 | 0.01 |  |  |
| max_d13C | **Forest** | **4** | **0.12** | **0.03** | **6.87** | **0.001** |
|  | Region | 1 | 0.02 | 0.02 | 3.27 | 0.078 |
|  | Residuals | 34 | 0.15 | 0.00 |  |  |
| max_d15N | Forest | 4 | 0.04 | 0.01 | 2.27 | 0.082 |
|  | Region | 1 | 0.00 | 0.00 | 0.12 | 0.735 |
|  | Residuals | 34 | 0.16 | 0.00 |  |  |
| range_d13C | Forest | 4 | 0.03 | 0.01 | 0.48 | 0.750 |
|  | Region | 1 | 0.01 | 0.01 | 0.31 | 0.582 |
|  | Residuals | 34 | 0.59 | 0.02 |  |  |
| range_d15N | **Forest** | **4** | **0.29** | **0.07** | **4.00** | **0.009** |
|  | Region | 1 | 0.03 | 0.03 | 1.35 | 0.252 |
|  | Residuals | 34 | 0.61 | 0.02 |  |  |
| IPos_d13C | **Forest** | **4** | **0.16** | **0.04** | **5.80** | **0.001** |
|  | **Region** | **1** | **0.06** | **0.06** | **5.77** | **0.021** |
|  | Residuals | 34 | 0.24 | 0.01 |  |  |
| IPos_d15N | Forest | 4 | 0.01 | 0.00 | 0.48 | 0.747 |
|  | Region | 1 | 0.00 | 0.00 | 0.08 | 0.779 |
|  | Residuals | 34 | 0.23 | 0.01 |  |  |
| IRic | Forest | 4 | 0.01 | 0.00 | 1.93 | 0.127 |
|  | Region | 1 | 0.00 | 0.00 | 0.00 | 0.973 |
|  | Residuals | 34 | 0.05 | 0.00 |  |  |
| IDiv | Forest | 4 | 0.03 | 0.01 | 0.38 | 0.819 |
|  | Region | 1 | 0.02 | 0.02 | 0.91 | 0.347 |
|  | Residuals | 34 | 0.71 | 0.02 |  |  |
| IDis | Forest | 4 | 0.04 | 0.01 | 0.38 | 0.820 |
|  | **Region** | **1** | **0.11** | **0.11** | **4.88** | **0.033** |
|  | Residuals | 34 | 0.84 | 0.02 |  |  |
| IEve | Forest | 4 | 0.04 | 0.01 | 0.45 | 0.768 |
|  | Region | 1 | 0.02 | 0.02 | 1.21 | 0.279 |
|  | Residuals | 34 | 0.69 | 0.02 |  |  |
| IUni | Forest | 4 | 0.17 | 0.04 | 0.95 | 0.449 |
|  | Region | 1 | 0.06 | 0.06 | 1.22 | 0.278 |
|  | Residuals | 34 | 1.55 | 0.05 |  |  |
| Primary decomposers | |  |  |  |  |  |
|  |  | df | SS | MS | F-value | p-value |
| min_d13C | Forest | 4 | 0.13 | 0.03 | 1.40 | 0.407 |
|  | Region | 1 | 0.20 | 0.20 | 8.63 | 0.022 |
|  | Residuals | 3 | 0.07 | 0.02 |  |  |
| min_d15N | Forest | 4 | 0.03 | 0.01 | 0.14 | 0.956 |
|  | Region | 1 | 0.00 | 0.00 | 0.13 | 0.731 |
|  | Residuals | 3 | 0.18 | 0.06 |  |  |
| max_d13C | Forest | 4 | 0.07 | 0.02 | 5.21 | 0.103 |
|  | **Region** | **1** | **0.06** | **0.06** | **8.31** | **0.024** |
|  | Residuals | 3 | 0.01 | 0.00 |  |  |
| max_d15N | Forest | 4 | 0.04 | 0.01 | 0.50 | 0.744 |
|  | Region | 1 | 0.00 | 0.00 | 0.14 | 0.718 |
|  | Residuals | 3 | 0.05 | 0.02 |  |  |
| range_d13C | Forest | 4 | 0.07 | 0.02 | 0.75 | 0.619 |
|  | Region | 1 | 0.04 | 0.04 | 2.01 | 0.199 |
|  | Residuals | 3 | 0.07 | 0.02 |  |  |
| range_d15N | Forest | 4 | 0.12 | 0.03 | 0.30 | 0.865 |
|  | Region | 1 | 0.01 | 0.01 | 0.19 | 0.679 |
|  | Residuals | 3 | 0.30 | 0.10 |  |  |
| IPos_d13C | Forest | 4 | 0.11 | 0.03 | 4.34 | 0.129 |
|  | **Region** | **1** | **0.11** | **0.11** | **7.06** | **0.033** |
|  | Residuals | 3 | 0.02 | 0.01 |  |  |
| IPos_d15N | Forest | 4 | 0.02 | 0.01 | 0.55 | 0.719 |
|  | Region | 1 | 0.00 | 0.00 | 0.03 | 0.871 |
|  | Residuals | 3 | 0.03 | 0.01 |  |  |
| IRic | Forest | 4 | 0.03 | 0.01 | 0.85 | 0.578 |
|  | Region | 1 | 0.00 | 0.00 | 0.11 | 0.751 |
|  | Residuals | 3 | 0.03 | 0.01 |  |  |
| IDiv | Forest | 4 | 0.17 | 0.04 | 1.68 | 0.350 |
|  | Region | 1 | 0.00 | 0.00 | 0.05 | 0.836 |
|  | Residuals | 3 | 0.08 | 0.03 |  |  |
| IDis | Forest | 4 | 0.12 | 0.03 | 0.40 | 0.802 |
|  | Region | 1 | 0.06 | 0.06 | 1.34 | 0.285 |
|  | Residuals | 3 | 0.23 | 0.08 |  |  |
| IEve | Forest | 4 | 0.08 | 0.02 | 1.59 | 0.367 |
|  | **Region** | **1** | **0.22** | **0.22** | **11.09** | **0.013** |
|  | Residuals | 3 | 0.04 | 0.01 |  |  |
| IUni | Forest | 4 | 0.08 | 0.02 | 0.55 | 0.717 |
|  | Region | 1 | 0.00 | 0.00 | 0.02 | 0.896 |
|  | Residuals | 3 | 0.11 | 0.04 |  |  |
| Secondary decomposers | |  |  |  |  |  |
|  |  | df | SS | MS | F-value | p-value |
| min_d13C | Forest | 4 | 0.22 | 0.05 | 2.69 | 0.181 |
|  | Region | 1 | 0.01 | 0.01 | 0.26 | 0.622 |
|  | Residuals | 4 | 0.08 | 0.02 |  |  |
| min_d15N | Forest | 4 | 0.17 | 0.04 | 1.82 | 0.289 |
|  | Region | 1 | 0.10 | 0.10 | 3.01 | 0.121 |
|  | Residuals | 4 | 0.10 | 0.02 |  |  |
| max_d13C | Forest | 4 | 0.03 | 0.01 | 1.67 | 0.315 |
|  | Region | 1 | 0.00 | 0.00 | 0.02 | 0.903 |
|  | Residuals | 4 | 0.02 | 0.00 |  |  |
| max_d15N | Forest | 4 | 0.00 | 0.00 | 0.22 | 0.914 |
|  | Region | 1 | 0.00 | 0.00 | 0.13 | 0.730 |
|  | Residuals | 4 | 0.02 | 0.01 |  |  |
| range_d13C | Forest | 4 | 0.12 | 0.03 | 0.96 | 0.516 |
|  | Region | 1 | 0.01 | 0.01 | 0.25 | 0.630 |
|  | Residuals | 4 | 0.13 | 0.03 |  |  |
| range_d15N | Forest | 4 | 0.16 | 0.04 | 2.65 | 0.184 |
|  | Region | 1 | 0.09 | 0.09 | 3.10 | 0.116 |
|  | Residuals | 4 | 0.06 | 0.02 |  |  |
| IPos_d13C | Forest | 4 | 0.08 | 0.02 | 1.38 | 0.380 |
|  | Region | 1 | 0.03 | 0.03 | 1.53 | 0.251 |
|  | Residuals | 4 | 0.06 | 0.01 |  |  |
| IPos_d15N | Forest | 4 | 0.02 | 0.01 | 0.75 | 0.605 |
|  | Region | 1 | 0.02 | 0.02 | 2.60 | 0.146 |
|  | Residuals | 4 | 0.03 | 0.01 |  |  |
| IRic | Forest | 4 | 0.01 | 0.00 | 0.44 | 0.774 |
|  | Region | 1 | 0.00 | 0.00 | 1.79 | 0.218 |
|  | Residuals | 4 | 0.01 | 0.00 |  |  |
| IDiv | Forest | 4 | 0.07 | 0.02 | 0.40 | 0.799 |
|  | Region | 1 | 0.00 | 0.00 | 0.04 | 0.840 |
|  | Residuals | 4 | 0.19 | 0.05 |  |  |
| IDis | Forest | 4 | 0.07 | 0.02 | 0.32 | 0.851 |
|  | Region | 1 | 0.00 | 0.00 | 0.08 | 0.784 |
|  | Residuals | 4 | 0.20 | 0.05 |  |  |
| IEve | Forest | 4 | 0.04 | 0.01 | 1.50 | 0.353 |
|  | Region | 1 | 0.03 | 0.03 | 3.66 | 0.092 |
|  | Residuals | 4 | 0.03 | 0.01 |  |  |
| IUni | Forest | 4 | 0.04 | 0.01 | 0.91 | 0.537 |
|  | Region | 1 | 0.01 | 0.01 | 0.61 | 0.456 |
|  | Residuals | 4 | 0.05 | 0.01 |  |  |
| Predators |  |  |  |  |  |  |
|  |  | df | SS | MS | F-value | p-value |
| **min_d13C** | **Forest** | **4** | **0.20** | **0.05** | **6.85** | **0.045** |
|  | Region | 1 | 0.02 | 0.02 | 0.80 | 0.397 |
|  | Residuals | 4 | 0.03 | 0.01 |  |  |
| **min_d15N** | **Forest** | **4** | **0.05** | **0.01** | **227.41** | **0.001** |
|  | Region | 1 | 0.00 | 0.00 | 0.03 | 0.873 |
|  | Residuals | 4 | 0.00 | 0.00 |  |  |
| max_d13C | Forest | 4 | 0.05 | 0.01 | 2.27 | 0.224 |
|  | Region | 1 | 0.00 | 0.00 | 0.37 | 0.561 |
|  | Residuals | 4 | 0.02 | 0.01 |  |  |
| max_d15N | Forest | 4 | 0.10 | 0.02 | 0.41 | 0.794 |
|  | Region | 1 | 0.01 | 0.01 | 0.31 | 0.595 |
|  | Residuals | 4 | 0.24 | 0.06 |  |  |
| **range_d13C** | **Forest** | **4** | **0.15** | **0.04** | **31.63** | **0.003** |
|  | Region | 1 | 0.01 | 0.01 | 0.43 | 0.531 |
|  | Residuals | 4 | 0.00 | 0.00 |  |  |
| range_d15N | Forest | 4 | 0.13 | 0.03 | 0.56 | 0.706 |
|  | Region | 1 | 0.01 | 0.01 | 0.23 | 0.645 |
|  | Residuals | 4 | 0.23 | 0.06 |  |  |
| IPos_d13C | Forest | 4 | 0.07 | 0.02 | 4.81 | 0.079 |
|  | Region | 1 | 0.01 | 0.01 | 0.69 | 0.430 |
|  | Residuals | 4 | 0.01 | 0.00 |  |  |
| IPos_d15N | Forest | 4 | 0.02 | 0.01 | 3.14 | 0.147 |
|  | Region | 1 | 0.01 | 0.01 | 2.98 | 0.123 |
|  | Residuals | 4 | 0.01 | 0.00 |  |  |
| IRic | Forest | 4 | 0.03 | 0.01 | 1.91 | 0.274 |
|  | Region | 1 | 0.00 | 0.00 | 0.32 | 0.588 |
|  | Residuals | 4 | 0.02 | 0.00 |  |  |
| IDiv | Forest | 4 | 0.04 | 0.01 | 0.70 | 0.631 |
|  | Region | 1 | 0.02 | 0.02 | 1.79 | 0.218 |
|  | Residuals | 4 | 0.05 | 0.01 |  |  |
| IDis | Forest | 4 | 0.02 | 0.01 | 0.50 | 0.742 |
|  | Region | 1 | 0.01 | 0.01 | 1.53 | 0.251 |
|  | Residuals | 4 | 0.04 | 0.01 |  |  |
| IEve | Forest | 4 | 0.09 | 0.02 | 0.81 | 0.576 |
|  | Region | 1 | 0.00 | 0.00 | 0.03 | 0.857 |
|  | Residuals | 4 | 0.11 | 0.03 |  |  |
| IUni | Forest | 4 | 0.02 | 0.01 | 3.02 | 0.155 |
|  | Region | 1 | 0.00 | 0.00 | 0.11 | 0.745 |
|  | Residuals | 4 | 0.01 | 0.00 |  |  |


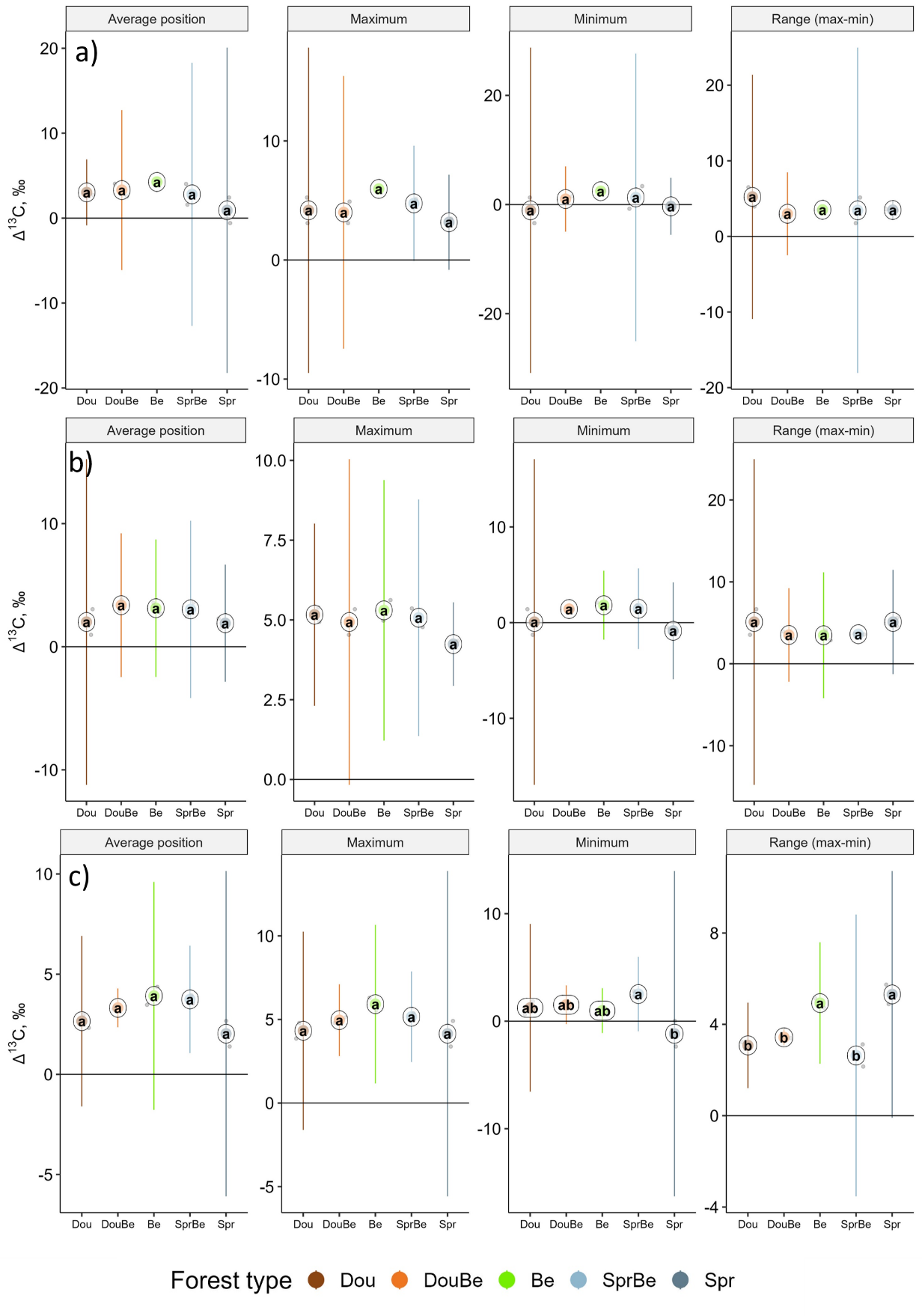


c)

**Figure S1:** One-dimensional metrics (average position, maximum, minimum, and range between minimum and maximum) for ∆^13^C values of macrofauna (a) primary decomposers, (b) secondary decomposers and (c) predators in Douglas fir (Dou), Douglas fir-Beech (DouBe), beech (Be), spruce-beech (SpBe) and spruce (Sp); means (circles) and confidence intervals. Values sharing the same letter do not differ significantly (Tukey’s HSD test, P < 0.05).


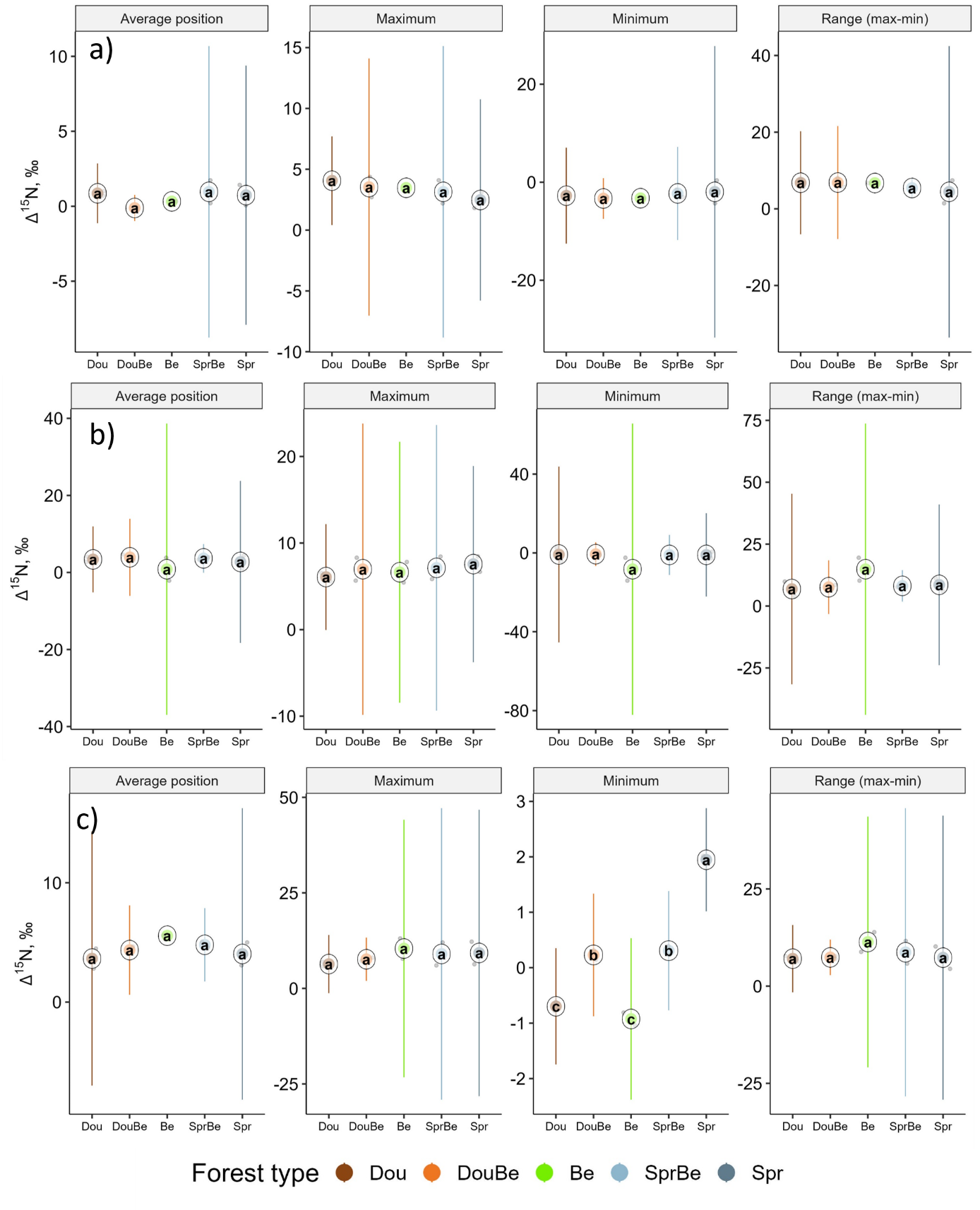


**Figure S2:** One-dimensional metrics for ∆^15^N of macrofauna guilds (a) primary decomposers, (b) Secondary decomposers and (c) predators in Douglas fir (Dou), Douglas fir-Beech (DouBe), beech (Be), spruce-beech (SpBe) and spruce (Sp) for average position, maximum, minimum, and range between minimum and maximum with means (circles) and confidence intervals. Values sharing the same letter do not differ significantly (Tukey’s HSD test, P < 0.05).

b)

c)

c)


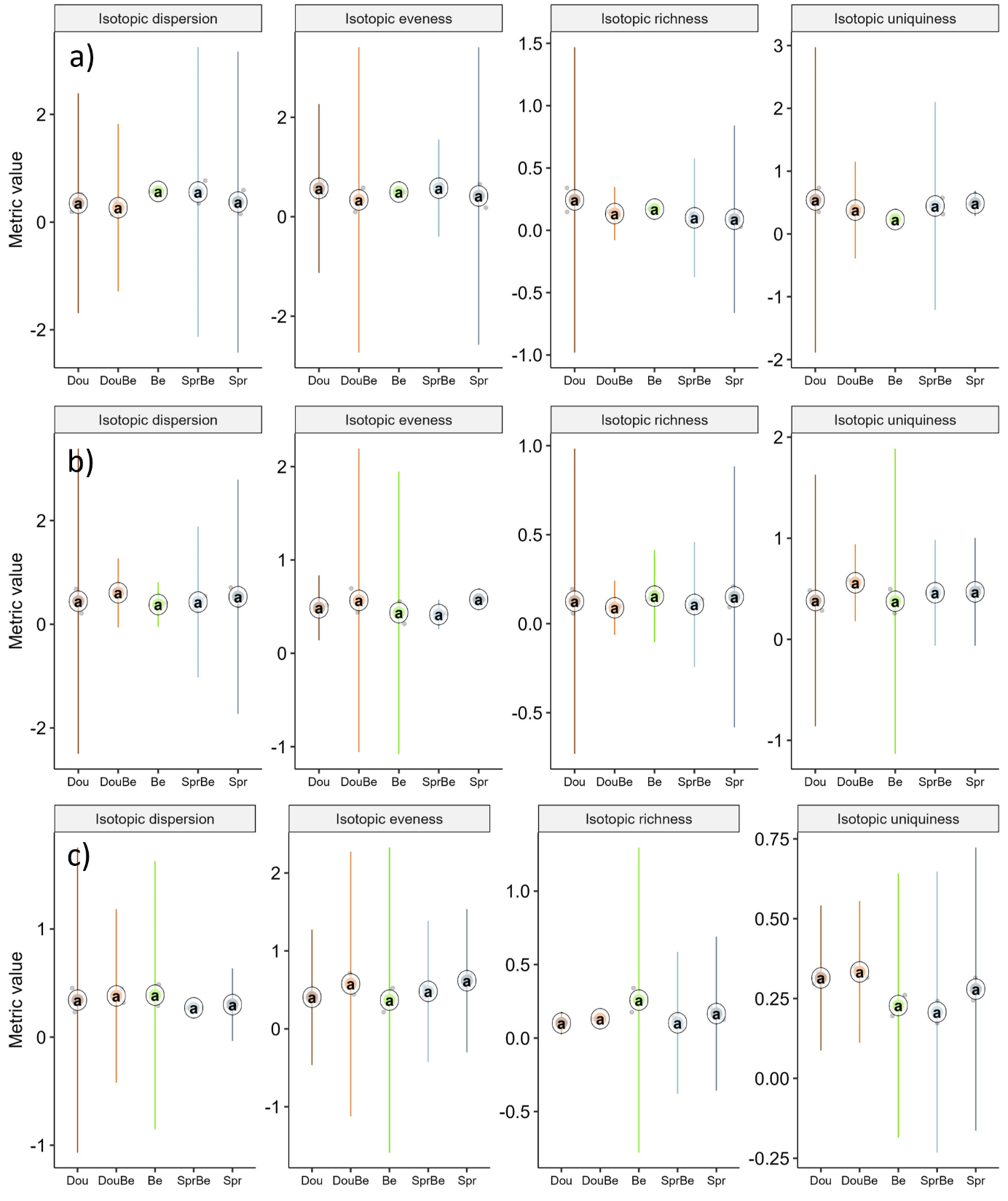


c)

d)

**Figure S3:** Multidimensional metrics for macrofauna guilds (a) primary decomposers, (b) secondary decomposers and (c) predators in Douglas fir (Dou), Douglas fir-Beech (DouBe), beech (Be), spruce-beech (SpBe) and spruce (Sp); for average position, maximum, minimum, and range between minimum and maximum with means (circles) in and confidence intervals. Values sharing the same letter do not differ significantly (Tukey’s HSD test, P < 0.05).
