# Supplementary material for: The influence of forest types including native and non‐native tree species on soil macrofauna depends on site conditions
Source: Ecol Evol. 2024 Sep 18;14(9):e70311. doi: 10.1002/ece3.70311 (PMC11410562; doi:10.1002/ece3.70311)
Supplement: Supplementary file 2 — Data S2. [file ECE3-14-e70311-s001.zip › Stable_Isotope_Raw_Data.docx]

Table S3: Stable Isotope Measurements for soil macrofauna

| Analysis |  | Sample | weight | restweight | totalweight | delta 15N | ATOM % | N amount | N concentration | delta 13C | ATOM % | C amount | C concentration |
| --- | --- | --- | --- | --- | --- | --- | --- | --- | --- | --- | --- | --- | --- |
| 29904 | 10 | 4_3-3_L2_Habrocerus_capillaricornis | 0.211 |  | 0.211 | -2.397 | 0.365428 | 19.98 | 9.47 | -26.79 | 107.638 | 108.02 | 51.19 |
| 29905 | 11 | 5_4-3_A2_Habrocerus_capillaricornis | 0.201 |  | 0.201 | -1.814 | 0.365641 | 23.86 | 11.87 | -22.99 | 108.041 | 101.21 | 50.35 |
| 29906 | 12 | 6_7-4_L1_Habrocerus_capillaricornis | 0.18 |  | 0.18 | -2.889 | 0.365248 | 21.18 | 11.77 | -24.86 | 107.842 | 93 | 51.67 |
| 29907 | 13 | 7_1-4_L2_Otiorhynchus_sp | 0.711 | 2.493 | 3.204 | -2.129 | 0.365526 | 67.39 | 9.48 | -25.49 | 107.776 | 349.19 | 49.11 |
| 29908 | 14 | 8_4-5_L2_Atheta_sp | 0.11 |  | 0.11 | -0.849 | 0.365993 | 11.89 | 10.81 | -26.06 | 107.715 | 59.1 | 53.73 |
| 29909 | 15 | 9_6-3_L2_Atheta_sp | 0.116 |  | 0.116 | 0.523 | 0.366494 | 14.17 | 12.21 | -24.98 | 107.829 | 65.88 | 56.8 |
| 29910 | 16 | 11_8-3_A2_Othius_punctulatus | 0.756 | 2.475 | 3.231 | -0.148 | 0.366249 | 83.9 | 11.1 | -25.29 | 107.796 | 377.52 | 49.94 |
| 29911 | 17 | 12_2-4_A1_Othius_punctulatus | 0.417 |  | 0.417 | 1.245 | 0.366757 | 49.57 | 11.89 | -25.15 | 107.811 | 200.87 | 48.17 |
| 29912 | 18 | 13_4-1_A1_Othius_punctulatus | 0.432 |  | 0.432 | 1.466 | 0.366838 | 46.87 | 10.85 | -27.26 | 107.588 | 220.51 | 51.04 |
| 29913 | 19 | 14_5-5_L2_Strophosoma_sp | 0.779 | 2.902 | 3.681 | -1.523 | 0.365747 | 86.4 | 11.09 | -26.22 | 107.699 | 374.77 | 48.11 |
| 29916 | 22 | 15_7-1_L2_Strophosoma_sp | 0.449 | 2.79 | 3.239 | 0.568 | 0.36651 | 49.25 | 10.97 | -25.04 | 107.823 | 216.26 | 48.17 |
| 29917 | 23 | 16_2-3_L1_Strophosoma_sp | 1.006 | 2.813 | 3.819 | 2.258 | 0.367127 | 123.65 | 12.29 | -26.75 | 107.642 | 447.7 | 44.5 |
| 29918 | 24 | 17_2-4_L1_Pella_laticollis | 0.11 |  | 0.11 | -1.551 | 0.365737 | 12.11 | 11.01 | -26.12 | 107.709 | 57.59 | 52.36 |
| 29919 | 25 | 18_7-1_L2_Notiophilus_bigutattus | 0.764 | 1.08 | 1.844 | -0.838 | 0.365997 | 93.74 | 12.27 | -27.06 | 107.609 | 365.26 | 47.81 |
| 29920 | 26 | 19_8-5_L2_Notiophilus_bigutattus | 0.945 | 1.175 | 2.12 | -4.311 | 0.364729 | 110.25 | 11.67 | -27.93 | 107.517 | 456.38 | 48.29 |
| 29921 | 27 | 20_2-5_A1_Notiophilus_bigutattus | 0.979 | 0.888 | 1.867 | -2.639 | 0.36534 | 110.98 | 11.34 | -28.49 | 107.458 | 469.51 | 47.96 |
| 29922 | 28 | 21_3-3_L2_Loricera_pilicornis | 0.785 | 4.357 | 5.142 | 7.479 | 0.369032 | 82.53 | 10.51 | -28.88 | 107.416 | 400.66 | 51.04 |
| 29923 | 29 | 22_3-2_A1_Agriotes_acuminatus | 0.611 | 1.084 | 1.695 | 0.497 | 0.366484 | 73.92 | 12.1 | -26.56 | 107.662 | 290.4 | 47.53 |
| 29924 | 30 | 23_4-1_L1_Raphirus_sp | 0.817 | 4.081 | 4.898 | 0.442 | 0.366464 | 91.15 | 11.16 | -27.63 | 107.548 | 404.1 | 49.46 |
| 29925 | 31 | 24_4-1_L1_Lordithon_sp | 0.408 |  | 0.408 | -3.02 | 0.365201 | 45.08 | 11.05 | -31.97 | 107.089 | 199.6 | 48.92 |
| 29928 | 34 | 26_4-1_A1_Habrocerus_sp | 0.184 |  | 0.184 | -2.442 | 0.365412 | 19.16 | 10.41 | -26.74 | 107.643 | 90.56 | 49.22 |
| 29929 | 35 | 27_4-5_L1_Acalles_sp | 0.553 |  | 0.553 | 0.243 | 0.366392 | 60.23 | 10.89 | -31.14 | 107.176 | 262.94 | 47.55 |
| 29930 | 36 | 28_6-3_A2_Gabrius_sp | 0.547 |  | 0.547 | -0.486 | 0.366126 | 54.51 | 9.96 | -25.64 | 10.776 | 256.64 | 46.92 |
| 29931 | 37 | 29_6-5_A2_Gabrius_sp | 0.23 |  | 0.23 | 1.512 | 0.366855 | 26.81 | 11.66 | -23.97 | 107.937 | 116.45 | 50.63 |
| 29932 | 38 | 30_4-1_A1_Gabrius_sp | 0.761 |  | 0.761 | 0.556 | 0.366506 | 88.66 | 11.65 | -27.59 | 107.553 | 372.78 | 48.99 |
| 29933 | 39 | 34_7-5_L2_Atrectus_affinis | 0.374 |  | 0.374 | -0.729 | 0.366037 | 43.6 | 11.66 | -24.92 | 107.835 | 181.28 | 48.47 |
| 29934 | 40 | 35_4-5_L1_Atrectus_affinis | 0.477 |  | 0.477 | 1.13 | 0.366715 | 55.8 | 11.7 | -25.72 | 107.752 | 227.15 | 47.62 |
| 29935 | 41 | 36_5-3_L1_Atrectus_affinis | 0.846 | 3.061 | 3.907 | 0.213 | 0.366381 | 99.29 | 11.74 | -24.97 | 107.831 | 405.88 | 47.98 |
| 29936 | 42 | 37_5-5_A1_Dalopius_marginatus | 0.753 | 1.127 | 1.88 | 2.592 | 0.367249 | 89.02 | 11.82 | -24.32 | 1.079 | 382.99 | 50.86 |
| 29937 | 43 | 38_4-5_A1_Dalopius_margniatus | 0.655 | 2.78 | 3.435 | 1.57 | 0.366876 | 64.73 | 9.88 | -26.39 | 107.681 | 350.81 | 53.56 |
| 29940 | 46 | 39_4-4_A1_Dalopius_margniatus | 0.866 | 1.981 | 2.847 | 3.68 | 0.367646 | 84.88 | 9.8 | -24.84 | 107.845 | 487.13 | 56.25 |
| 29941 | 47 | 42_8-1_L2_Eusphalerum_sp | 0.287 |  | 0.287 | -2.79 | 0.365285 | 35.64 | 12.42 | -25.22 | 107.804 | 139.71 | 48.68 |
| 29942 | 48 | 43_5-4_L1_Bisnius_sp | 0.492 |  | 0.492 | -0.563 | 0.366097 | 57.39 | 11.66 | -24.74 | 107.855 | 242.59 | 49.31 |
| 29943 | 49 | 44_2-3_L2_Bisnius_sp | 0.441 |  | 0.441 | 2.594 | 0.367249 | 50.76 | 11.51 | -25.15 | 107.811 | 221.16 | 50.15 |
| 29944 | 50 | 45_7-5_L1_Neobisnius_sp | 0.241 |  | 0.241 | -1.803 | 0.365645 | 27.32 | 11.34 | -26.45 | 107.673 | 121.02 | 50.22 |
| 29945 | 51 | 46_7-3_L1_Neobisnius_sp | 0.38 |  | 0.38 | 2.015 | 0.367038 | 45.49 | 11.97 | -26.18 | 107.703 | 185.25 | 48.75 |
| 29946 | 52 | 47_6-3_L2_Neobisnius_sp | 0.227 |  | 0.227 | 1.403 | 0.366815 | 26.75 | 11.78 | -25.01 | 107.826 | 114.65 | 50.51 |
| 29947 | 53 | 49_3-4_L2_Microsaurus_sp | 0.299 |  | 0.299 | 6.861 | 0.368807 | 35.53 | 11.88 | -26.06 | 107.715 | 146.94 | 49.14 |
| 29948 | 54 | 51_6-5_L1_Ampedus_rufipennis | 0.889 | 6.091 | 6.98 | -2.161 | 0.365514 | 111.35 | 12.53 | -25.64 | 10.776 | 427.64 | 48.1 |
| 29949 | 55 | 52_7-5_L1_Phymatura_sp | 0.113 |  | 0.113 | -2.947 | 0.365227 | 14.05 | 12.43 | -26.07 | 107.714 | 62.91 | 55.67 |
| 30007 | 6 | 53_2-1_L1_Olophrum_sp | 0.265 |  | 0.265 | -1.406 | 0.36579 | 30.59 | 11.54 | -25.98 | 1.077.230 | 128.78 | 48.6 |
| 30008 | 7 | 54_4-2_L2_Olophrum_sp | 0.155 |  | 0.155 | -1.936 | 0.365596 | 17.31 | 11.17 | -26.07 | 1.077.139 | 76.42 | 49.31 |
| 30009 | 8 | 55_6-5_L2_Heterothops_dissimilis | 0.301 |  | 0.301 | 0.014 | 0.366308 | 35.84 | 11.91 | -24.12 | 1.079.175 | 142.12 | 47.22 |
| 30010 | 9 | 56_5-5_L2_Heterothops_dissimilis | 0.229 |  | 0.229 | 2.245 | 0.367122 | 26.38 | 11.52 | -27.25 | 1.075.903 | 111.07 | 48.5 |
| 30011 | 10 | 57_8-4_A2_Athous_subfucus | 0.715 | 2.48 | 3.195 | 0.134 | 0.366352 | 89.25 | 12.48 | -24.24 | 1.079.050 | 326.26 | 45.63 |
| 30012 | 11 | 58_7-4_A2_Athous_subfucus | 0.945 | 3.603 | 4.548 | -0.055 | 0.366283 | 108.75 | 11.51 | -25.85 | 1.077.370 | 474.01 | 50.16 |
| 30013 | 12 | 61_1-3_L2_Trichoniscus_pusillus | 0.205 |  | 0.205 | -1.31 | 0.365825 | 19.98 | 9.75 | -25.14 | 1.078.114 | 83.39 | 40.68 |
| 30014 | 13 | 62_2-4_A2_Trichoniscus_pusillus | 0.14 |  | 0.14 | -0.793 | 0.366013 | 14.5 | 10.36 | -25.79 | 1.077.434 | 60.44 | 43.17 |
| 30015 | 14 | 63_3-1_L2_Trichoniscus_pusillus | 0.243 |  | 0.243 | -0.192 | 0.366233 | 19.22 | 7.91 | -25.21 | 1.078.038 | 81.13 | 33.39 |
| 30016 | 15 | 64_3-2_A2_Trichoniscus_pusillus | 0.277 |  | 0.277 | -0.145 | 0.36625 | 21.63 | 7.81 | -24.94 | 1.078.325 | 90.34 | 32.61 |
| 30019 | 18 | 65_4-2_A2_Trichoniscus_pusillus | 0.166 |  | 0.166 | -1.047 | 0.365921 | 14.23 | 8.57 | -25.61 | 1.077.620 | 63.59 | 38.31 |
| 30020 | 19 | 66_4-3_L2_Trichoniscus_pusillus | 0.126 |  | 0.126 | -1.724 | 0.365674 | 11.21 | 8.9 | -24.82 | 1.078.441 | 49.55 | 39.32 |
| 30021 | 20 | 67_5-1_L2_Trichoniscus_pusillus | 0.214 |  | 0.214 | 2.159 | 0.367091 | 19.49 | 9.11 | -24.71 | 1.078.558 | 84.35 | 39.41 |
| 30022 | 21 | 68_5-4_L2_Trichoniscus_pusillus | 0.262 |  | 0.262 | -2.149 | 0.365519 | 20.98 | 8.01 | -24 | 1.079.300 | 87.91 | 33.56 |
| 30023 | 22 | 69_5-5_L2_Trichoniscus_pusillus | 0.147 |  | 0.147 | 0.147 | 0.366356 | 10.69 | 7.27 | -25.86 | 1.077.354 | 49.93 | 33.96 |
| 30024 | 23 | 70_6-1_L2_Trichoniscus_pusillus | 0.145 |  | 0.145 | -1.372 | 0.365802 | 11.81 | 8.15 | -24.94 | 1.078.323 | 55.06 | 37.97 |
| 30025 | 24 | 71_6-3_L2_Trichoniscus_pusillus | 0.139 |  | 0.139 | -1.236 | 0.365852 | 11.24 | 8.09 | -24.07 | 1.079.231 | 49.92 | 35.91 |
| 30026 | 25 | 72_8-1_L2_Trichoniscus_pusillus | 0.276 |  | 0.276 | -0.82 | 0.366004 | 20.75 | 7.52 | -24.14 | 1.079.158 | 89.57 | 32.45 |
| 30027 | 26 | 73_8-3_L2_Trichoniscus_pusillus | 0.312 |  | 0.312 | -1.882 | 0.365616 | 27.09 | 8.68 | -25.84 | 1.077.377 | 113.89 | 36.5 |
| 30028 | 27 | 74_1-2_L1_Trichoniscus_pusillus | 0.224 |  | 0.224 | -1.268 | 0.36584 | 17.48 | 7.8 | -25.47 | 1.077.760 | 79.99 | 35.71 |
| 30031 | 30 | 75_2-3_L1_Trichoniscus_pusillus | 0.252 |  | 0.252 | -2.064 | 0.36555 | 22.16 | 8.8 | -25.52 | 1.077.709 | 92.56 | 36.73 |
| 30032 | 31 | 76_3-3_A1_Trichoniscus_pusillus | 0.192 |  | 0.192 | -0.828 | 0.366001 | 17.15 | 8.93 | -25.24 | 1.078.007 | 72.13 | 37.57 |
| 30033 | 32 | 77_4-1_A2_Trichoniscus_pusillus | 0.321 |  | 0.321 | 0.409 | 0.366452 | 25.65 | 7.99 | -25.45 | 1.077.784 | 112.28 | 34.98 |
| 30034 | 33 | 78_4-5_L1_Trichoniscus_pusillus | 0.242 |  | 0.242 | -1.558 | 0.365734 | 21.2 | 8.76 | -27.07 | 1.076.092 | 88.09 | 36.4 |
| 30035 | 34 | 79_5-2_L1_Trichoniscus_pusillus | 0.179 |  | 0.179 | -0.042 | 0.366288 | 15.42 | 8.61 | -24.64 | 1.078.628 | 67.32 | 37.61 |
| 30036 | 35 | 80_6-1_L2_Oniscus_asellus | 0.961 | 7.98 | 8.941 | -7.29 | 0.363642 | 53.93 | 5.61 | -26.62 | 1.076.561 | 270.16 | 28.11 |
| 30037 | 36 | 81_6-3_L2_Oniscus_asellus | 0.41 |  | 0.41 | -2.9 | 0.365244 | 32.59 | 7.95 | -25.04 | 1.078.220 | 153.05 | 37.33 |
| 30038 | 37 | 82_8-3_L2_Oniscus_asellus | 0.997 | 10.694 | 11.691 | -4.988 | 0.364483 | 52.99 | 5.32 | -24.44 | 1.078.845 | 297.75 | 29.86 |
| 30039 | 38 | 83_4-1_L1_Oniscus_asellus | 0.795 | 11.668 | 12.463 | -4.102 | 0.364806 | 40.6 | 5.11 | -25.73 | 1.077.497 | 216.51 | 27.23 |
| 30040 | 39 | 84_5-1_L2_Armadillidium_vulgare | 0.972 |  | 0.972 | -1.048 | 0.365921 | 73.79 | 7.59 | -26.88 | 1.076.287 | 330.82 | 34.03 |
| 30043 | 42 | 85_5-5_L2_Armadillidium_vulgare | 0.754 | 0.837 | 1.591 | -1.744 | 0.365666 | 36.68 | 4.86 | -28.38 | 1.074.718 | 241.12 | 31.98 |
| 30044 | 43 | 86_8-1_L2_Armadillidium_vulgare | 0.658 | 0.676 | 1.334 | -5.212 | 0.364401 | 45.04 | 6.84 | -25.69 | 1.077.534 | 199.75 | 30.36 |
| 30045 | 44 | 87_8-2_L2_Armadillidium_vulgare | 0.632 | 0.608 | 1.24 | -5.94 | 0.364135 | 53.81 | 8.51 | -27.23 | 1.075.925 | 227.21 | 35.95 |
| 30046 | 45 | 88_8-4_L2_Armadillidium_vulgare | 0.978 | 1.888 | 2.866 | -5.082 | 0.364448 | 60.85 | 6.22 | -25.18 | 1.078.071 | 277.02 | 28.32 |
| 30047 | 46 | 89_1-3_B1_Julus_scandinavius | 0.838 | 13.711 | 14.549 | -1.898 | 0.36561 | 43.32 | 5.17 | -24.55 | 1.078.730 | 239.77 | 28.61 |
| 30048 | 47 | 90_5-2_B1_Julus_scandinavius | 0.403 |  | 0.403 | -0.618 | 0.366078 | 28.48 | 7.07 | -24.44 | 1.078.844 | 129.45 | 32.12 |
| 30049 | 48 | 91_3-1_L1_Mycogena_germanica | 0.626 | 0.615 | 1.241 | -0.159 | 0.366245 | 51.51 | 8.23 | -25.19 | 1.078.055 | 200.17 | 31.98 |
| 30050 | 49 | 92_3-3_A2_Glomeris_marginata | 0.729 |  | 0.729 | -3.871 | 0.36489 | 35.16 | 4.82 | -24.26 | 1.079.035 | 183.27 | 25.14 |
| 30051 | 50 | 93_4-1_L1_Glomeris_marginata | 0.542 |  | 0.542 | -5.612 | 0.364255 | 32.66 | 6.03 | -25.48 | 1.077.750 | 150.28 | 27.73 |
| 30052 | 51 | 94_4-4_A1_Glomeris_marginata | 0.996 | 0.637 | 1.633 | -4.629 | 0.364613 | 64.81 | 6.51 | -28.41 | 1.074.690 | 302.23 | 30.34 |
| 30055 | 54 | 182_5-3_A1_Schendyla_nemorensis | 0.227 |  | 0.227 | 0.468 | 0.366474 | 30.1 | 13.26 | -24.53 | 1.078.748 | 114.89 | 50.61 |
| 30056 | 55 | 183_5-4_A1_Schendyla_nemorensis | 0.143 |  | 0.143 | 0.799 | 0.366594 | 18.7 | 13.08 | -23.81 | 1.079.502 | 71.75 | 50.17 |
| 30057 | 56 | 184_5-5_L1_Schendyla_nemorensis | 0.963 | 0.048 | 1.011 | 1.754 | 0.366943 | 122.75 | 12.75 | -26.17 | 1.077.030 | 445.56 | 46.27 |
| 30058 | 57 | 185_7-1_B1_Schendyla_nemorensis | 0.484 |  | 0.484 | 1.517 | 0.366856 | 59.79 | 12.35 | -25.2 | 1.078.044 | 220.69 | 45.6 |
| 30059 | 58 | 186_7-2_B1_Schendyla_nemorensis | 0.421 |  | 0.421 | 2.098 | 0.367069 | 55.18 | 13.11 | -24.59 | 1.078.683 | 190.63 | 45.28 |
| 30060 | 59 | 187_7-3_A1_Schendyla_nemorensis | 0.619 |  | 0.619 | 2.298 | 0.367141 | 65.89 | 10.64 | -26.21 | 1.076.991 | 302.57 | 48.88 |
| 30061 | 60 | 188_8-1_L1_Schendyla_nemorensis | 0.176 |  | 0.176 | 1.362 | 0.3668 | 23.5 | 13.35 | -23.42 | 1.079.904 | 83.81 | 47.62 |
| 30062 | 61 | 189_8-2_B1_Schendyla_nemorensis | 0.4 |  | 0.4 | 1.768 | 0.366948 | 53.32 | 13.33 | -24.5 | 1.078.778 | 182.54 | 45.63 |
| 30063 | 62 | 190_8-4_B1_Schendyla_nemorensis | 0.368 |  | 0.368 | 0.622 | 0.36653 | 47.11 | 12.8 | -23.93 | 1.079.376 | 164.61 | 44.73 |
| 30064 | 63 | 191_8-5_B1_Schendyla_nemorensis | 0.147 |  | 0.147 | 0.686 | 0.366553 | 18.59 | 12.64 | -23.67 | 1.079.645 | 72.65 | 49.42 |
| 30067 | 66 | 192_2-1_B2_Schendyla_nemorensis | 0.422 |  | 0.422 | 3.477 | 0.367572 | 51.68 | 12.25 | -24.76 | 1.078.508 | 189.25 | 44.85 |
| 30068 | 67 | 193_4-3_B2_Schendyla_nemorensis | 0.966 |  | 0.966 | 0.581 | 0.366515 | 123.22 | 12.76 | -25.57 | 1.077.660 | 438.93 | 45.44 |
| 30069 | 68 | 194_6-4_B2_Schendyla_nemorensis | 0.38 |  | 0.38 | -0.083 | 0.366273 | 42.65 | 11.22 | -25.25 | 1.077.999 | 184.87 | 48.65 |
| 30070 | 69 | 198_8-2_A1_Geophilus_truncorum | 0.333 |  | 0.333 | 1.334 | 0.36679 | 17.61 | 5.29 | -24.27 | 1.079.020 | 67.69 | 20.33 |
| 30071 | 70 | 199_8-3_B1_Geophilus_truncorum | 0.126 |  | 0.126 | -1.499 | 0.365756 | 16.62 | 13.19 | -24.06 | 1.079.240 | 63.52 | 50.42 |
| 30072 | 71 | 200_8-4_A1_Geophilus_truncorum | 0.116 |  | 0.116 | -0.334 | 0.366181 | 15.2 | 13.11 | -24.17 | 1.079.128 | 59.79 | 51.54 |
| 30073 | 72 | 201_8-5_A1_Geophilus_truncorum | 0.454 |  | 0.454 | -0.176 | 0.366239 | 57.95 | 12.76 | -23.08 | 1.080.269 | 205.51 | 45.27 |
| 30074 | 73 | 202_1-4_L2_Geophilus_truncorum | 0.109 |  | 0.109 | 2.755 | 0.367308 | 14.9 | 13.67 | -24.15 | 1.079.148 | 57.9 | 53.12 |
| 30075 | 74 | 203_5-5_A2_Geophilus_truncorum | 0.205 |  | 0.205 | 2.727 | 0.367298 | 27.33 | 13.33 | -23.68 | 1.079.640 | 102.85 | 50.17 |
| 30076 | 75 | 204_1-3_L1_Lithobius_melanops | 0.395 |  | 0.395 | 0.44 | 0.366463 | 41.38 | 10.48 | -25.24 | 1.078.005 | 180.5 | 45.7 |
| 30079 | 78 | 205_2-3_A1_Lithobius_melanops | 0.67 | 1.342 | 2.012 | -0.691 | 0.366051 | 81.91 | 12.23 | -26.2 | 107.701 | 295.65 | 44.13 |
| 30080 | 79 | 206_4-1_L1_Lithobius_melanops | 0.924 | 1.645 | 2.569 | -0.903 | 0.365973 | 122.25 | 13.23 | -25.9 | 107.731 | 423.74 | 45.86 |
| 30081 | 80 | 207_4-3_A1_Lithobius_melanops | 0.459 | 3.349 | 3.808 | -0.252 | 0.366211 | 58.93 | 12.84 | -25.06 | 107.819 | 208.51 | 45.43 |
| 30082 | 81 | 208_4-4_L1_Lithobius_melanops | 0.626 | 8.396 | 9.022 | -0.002 | 0.366302 | 75.02 | 11.98 | -24.6 | 107.867 | 269.39 | 43.03 |
| 30083 | 82 | 209_4-1_A1_Lithobius_lapidicola | 0.482 | 2.234 | 2.716 | -0.975 | 0.365947 | 62.48 | 12.96 | -26.63 | 107.655 | 215.41 | 44.69 |
| 30084 | 83 | 210_6-3_L1_Lithobius_lapidicola | 0.776 |  | 0.776 | -1.131 | 0.36589 | 106.16 | 13.68 | -24.86 | 107.841 | 353.12 | 45.51 |
| 30085 | 84 | 211_5-5_L1_Lithobius_lapidicola | 0.481 |  | 0.481 | -0.274 | 0.366203 | 65.18 | 13.55 | -28.44 | 107.466 | 217.9 | 45.3 |
| 30086 | 85 | 212_4-1_A1_Lithobius_muticus | 0.856 |  | 0.856 | -1.945 | 0.365593 | 115.12 | 13.45 | -26.65 | 107.653 | 386.4 | 45.14 |
| 30087 | 86 | 213_5-3_L1_Lithobius_muticus | 0.724 | 2.805 | 3.529 | -0.854 | 0.365991 | 98.36 | 13.59 | -23.69 | 107.963 | 322.7 | 44.57 |
| 30088 | 87 | 214_5-5_A1_Lithobius_muticus | 0.63 | 1.132 | 1.762 | 1.22 | 0.366748 | 86.26 | 13.69 | -25.58 | 107.765 | 287.16 | 45.58 |
| 30091 | 90 | 215_7-4_L1_Lithobius_muticus | 0.426 | 3.303 | 3.729 | -1.865 | 0.365622 | 54.85 | 12.88 | -25.26 | 107.799 | 198.37 | 46.57 |
| 30092 | 91 | 216_5-2_B1_Cryptops_hortensis | 0.379 |  | 0.379 | 3.628 | 0.367627 | 48.55 | 12.81 | -22.98 | 108.037 | 169.13 | 44.62 |
| 30093 | 92 | 217_6-2_L2_Lithobius_agilis | 0.5 |  | 0.5 | -1.457 | 0.365771 | 67.18 | 13.44 | -24.23 | 107.907 | 225.83 | 45.17 |
| 30094 | 93 | 218_7-4_L1_Lithobius_erythrocephalus | 0.35 |  | 0.35 | -0.854 | 0.365991 | 46.49 | 13.28 | -24.52 | 107.876 | 159.76 | 45.65 |
| 30095 | 94 | 219_2-2_L2_Lithobius_erythrocephalus | 0.401 |  | 0.401 | -0.039 | 0.366289 | 52.11 | 13 | -24.97 | 107.829 | 181.54 | 45.27 |
| 30096 | 95 | 220_4-4_L2_Lithobius_erythrocephalus | 0.805 |  | 0.805 | 0.032 | 0.366314 | 102.56 | 12.74 | -24.85 | 107.842 | 346.04 | 42.99 |
| 30097 | 96 | 221_2-2_L1_Lithobius_curtipes | 0.836 | 1.599 | 2.435 | 0.534 | 0.366498 | 109.78 | 13.13 | -24.89 | 107.837 | 375.04 | 44.86 |
| 30098 | 97 | 223_4-2_L1_Lithobius_crassipes | 0.664 | 1.019 | 1.683 | 0.289 | 0.366408 | 87.59 | 13.19 | -25.08 | 107.817 | 298.03 | 44.88 |
| 30099 | 98 | 224_4-3_L1_Lithobius_crassipes | 0.795 | 0.482 | 1.277 | 0.557 | 0.366506 | 107.08 | 13.47 | -24.34 | 107.895 | 358.25 | 45.06 |
| 30100 | 99 | 225_5-2_L1_Lithobius_crassipes | 0.71 |  | 0.71 | 2.069 | 0.367058 | 93.59 | 13.18 | -23.79 | 107.952 | 315.33 | 44.41 |
| 30113 | 6 | 226_8-4_L1_Lithobius_crassipes | 0.46 | 1.066 | 1.526 | -1.717 | 0.365676 | 59.96 | 13.03 | -25.24 | 1.078.025 | 210.86 | 45.84 |
| 30114 | 7 | 227_8-1_L2_Lithobius_crassipes | 0.749 | 0.966 | 1.715 | -1.31 | 0.365825 | 103.67 | 13.84 | -24.11 | 1.079.240 | 349.39 | 46.65 |
| 30115 | 8 | 228_2-4_A1_Lithobius_aeruginosus | 0.169 |  | 0.169 | 0.05 | 0.366321 | 22.83 | 13.51 | -23.99 | 1.079.373 | 82.23 | 48.66 |
| 30116 | 9 | 229_2-3_L1_Lithobius_aeruginosus | 0.248 |  | 0.248 | 0.648 | 0.366539 | 33.81 | 13.63 | -24.54 | 1.078.783 | 121.93 | 49.16 |
| 30117 | 10 | 230_4-2_L1_Lithobius_aeruginosus | 0.1 |  | 0.1 | 0.495 | 0.366483 | 13.25 | 13.25 | -24.33 | 1.079.005 | 50.5 | 50.5 |
| 30118 | 11 | 232_1-3_L2_Geophilus_alpinus | 0.757 | 0.139 | 0.896 | 3.385 | 0.367538 | 99.23 | 13.11 | -24.56 | 1.078.754 | 351.07 | 46.38 |
| 30119 | 12 | 233_1-5_A2_Geophilus_alpinus | 0.217 |  | 0.217 | 1.627 | 0.366897 | 27.51 | 12.68 | -24.17 | 1.079.175 | 102.47 | 47.22 |
| 30120 | 13 | 235_7-3_L2_Agyrtidae | 0.192 |  | 0.192 | 2.151 | 0.367088 | 22.85 | 11.9 | -25.4 | 1.077.863 | 93.7 | 48.8 |
| 30121 | 14 | 237_4-4_L1_Agyrtidae | 0.147 |  | 0.147 | -2.167 | 0.365512 | 18.96 | 12.89 | -24.68 | 1.078.626 | 71.14 | 48.4 |
| 30122 | 15 | 242_3-2_B2_Carabidae | 0.483 | 3.036 | 3.519 | 1.945 | 0.367013 | 58.68 | 12.15 | -24.89 | 1.078.403 | 225.55 | 46.7 |
| 30125 | 18 | 244_6-3_A2_Carabidae | 0.35 | 0.821 | 1.171 | -2.391 | 0.36543 | 36.88 | 10.54 | -24.73 | 1.078.580 | 161.21 | 46.06 |
| 30126 | 19 | 248_4-4_A2_Curculionidae | 0.201 |  | 0.201 | 0.506 | 0.366488 | 23.34 | 11.61 | -26.32 | 1.076.872 | 92.82 | 46.18 |
| 30127 | 20 | 250_5-3_L2_Curculionidae | 0.319 |  | 0.319 | -5.745 | 0.364206 | 36.1 | 11.32 | -26.48 | 1.076.697 | 138.8 | 43.51 |
| 30128 | 21 | 251_5-2_L2_Crysomelidae | 0.611 |  | 0.611 | -1.038 | 0.365924 | 69.13 | 11.31 | -26.92 | 1.076.235 | 281.83 | 46.13 |
| 30129 | 22 | 253_6-1_L2_Lampyridae | 0.423 |  | 0.423 | -1.955 | 0.365589 | 57.25 | 13.53 | -25.26 | 1.078.008 | 200.45 | 47.39 |
| 30130 | 23 | 254_4-2_L2_Lampyridae | 0.316 |  | 0.316 | -1.478 | 0.365764 | 38.39 | 12.15 | -25.88 | 1.077.345 | 144.01 | 45.57 |
| 30131 | 24 | 255_8-1_L2_Lampyridae | 0.374 |  | 0.374 | -2.352 | 0.365444 | 43.62 | 11.66 | -23.95 | 1.079.405 | 171.42 | 45.84 |
| 30132 | 25 | 258_1-3_L2_Cantharidae | 0.92 |  | 0.92 | 0.862 | 0.366617 | 113.61 | 12.35 | -25.01 | 1.078.276 | 427.18 | 46.43 |
| 30133 | 26 | 259_1-4_L2_Cantharidae | 0.251 |  | 0.251 | -2.35 | 0.365445 | 30.88 | 12.3 | -26.19 | 1.077.009 | 115.56 | 46.04 |
| 30134 | 27 | 265_2-5_L2_Cantharidae | 0.26 |  | 0.26 | -4.814 | 0.364546 | 26.8 | 10.31 | -26.79 | 1.076.372 | 113.31 | 43.58 |
| 30137 | 30 | 272_4-2_L2_Cantharidae | 0.78 |  | 0.78 | 2.997 | 0.367397 | 3.97 | 0.51 | -24.28 | 1.079.056 | 19.57 | 2.51 |
| 30138 | 31 | 286_7-2_L2_Cantharidae | 0.117 |  | 0.117 | -0.215 | 0.366224 | 13.39 | 11.44 | -23.24 | 1.080.167 | 57.19 | 48.88 |
| 30139 | 32 | 289_7-5_L2_Cantharidae | 0.192 |  | 0.192 | -3.699 | 0.364953 | 23.67 | 12.33 | -25.12 | 1.078.162 | 92.13 | 47.99 |
| 30140 | 33 | 292_8-3_L2_Cantharidae | 0.902 |  | 0.902 | -3.027 | 0.365198 | 116.24 | 12.89 | -24.5 | 1.078.819 | 422.32 | 46.82 |
| 30141 | 34 | 295_1-1_B1_Elateridae | 0.648 |  | 0.648 | -3.04 | 0.365193 | 64.83 | 10.01 | -27.89 | 1.075.193 | 296.01 | 45.68 |
| 30142 | 35 | 296_1-2_A1_Elateridae | 0.491 |  | 0.491 | 1.618 | 0.366893 | 61.44 | 12.51 | -25.04 | 1.078.244 | 229.8 | 46.8 |
| 30143 | 36 | 297_1-4_A1_Elateridae | 0.529 |  | 0.529 | -0.566 | 0.366096 | 53.86 | 10.18 | -25.23 | 1.078.041 | 238.28 | 45.04 |
| 30144 | 37 | 298_1-5_B1_Elateridae | 0.951 |  | 0.951 | -1.512 | 0.365751 | 112.24 | 11.8 | -23.96 | 1.079.396 | 436.15 | 45.86 |
| 30145 | 38 | 299_2-2_A1_Elateridae | 0.613 | 1.005 | 1.618 | 6.747 | 0.368765 | 66.95 | 10.92 | -23.61 | 1.079.769 | 281.12 | 45.86 |
| 30146 | 39 | 300_2-3_A1_Elateridae | 0.359 |  | 0.359 | 3.726 | 0.367663 | 47.54 | 13.24 | -23.26 | 1.080.149 | 168.71 | 46.99 |
| 30149 | 42 | 301_2-4_A1_Elateridae | 1 |  | 1 | 2.166 | 0.367093 | 131.37 | 13.14 | -23.36 | 1.080.037 | 472.15 | 47.22 |
| 30150 | 43 | 302_2-5_A1_Elateridae | 0.978 |  | 0.978 | 0.618 | 0.366528 | 125.23 | 12.8 | -24.03 | 1.079.327 | 452.93 | 46.31 |
| 30151 | 44 | 303_3-1_B2_Elateridae | 0.664 | 1.049 | 1.713 | 0.564 | 0.366509 | 81.99 | 12.35 | -26.25 | 1.076.945 | 305.62 | 46.03 |
| 30152 | 45 | 305_3-3_A2_Elateridae | 0.515 | 3.658 | 4.173 | 3.011 | 0.367402 | 67.27 | 13.06 | -24.3 | 1.079.036 | 246.07 | 47.78 |
| 30153 | 46 | 306_3-4_A2_Elateridae | 0.86 |  | 0.86 | 1.129 | 0.366715 | 112.69 | 13.1 | -23.89 | 1.079.473 | 402.9 | 46.85 |
| 30154 | 47 | 307_3-5_B1_Elateridae | 0.758 |  | 0.758 | 1.798 | 0.366959 | 98.79 | 13.03 | -23.42 | 1.079.981 | 358.38 | 47.28 |
| 30155 | 48 | 308_4-1_A1_Elateridae | 0.892 |  | 0.892 | -0.107 | 0.366264 | 113.66 | 12.74 | -26.26 | 1.076.940 | 424.3 | 47.57 |
| 30156 | 49 | 310_4-3_B1_Elateridae | 0.866 |  | 0.866 | 1.418 | 0.36682 | 104 | 12.01 | -24.56 | 1.078.754 | 394.81 | 45.59 |
| 30157 | 50 | 311_4-4_A1_Elateridae | 0.528 | 2.222 | 2.75 | 3.113 | 0.367439 | 66.06 | 12.51 | -23.32 | 1.080.081 | 245.11 | 46.42 |
| 30158 | 51 | 312_4-5_A1_Elateridae | 0.236 |  | 0.236 | -1.084 | 0.365907 | 30.76 | 13.03 | -25.19 | 1.078.086 | 111.7 | 47.33 |
| 30161 | 54 | 95_5-5_A1_Glomeris_marginata | 0.607 | 2.425 | 3.032 | -7.523 | 0.363557 | 21.3 | 3.51 | -25.57 | 1.077.679 | 135.06 | 22.25 |
| 30162 | 55 | 96_6-3_L1_Glomeris_marginata | 0.831 |  | 0.831 | -5.153 | 0.364422 | 50.87 | 6.12 | -24.67 | 1.078.636 | 239.98 | 28.88 |
| 30163 | 56 | 97_7-3_A1_Glomeris_marginata | 0.963 | 0.755 | 1.718 | -4.131 | 0.364795 | 61 | 6.33 | -24.64 | 1.078.670 | 301.33 | 31.29 |
| 30164 | 57 | 98_4-5_L2_Glomeris_marginata | 0.827 | 6.093 | 6.92 | -3.505 | 0.365024 | 43.69 | 5.28 | -29.52 | 1.073.448 | 358.99 | 43.41 |
| 30165 | 58 | 99_5-3_A2_Glomeris_marginata | 0.729 | 13.929 | 14.658 | -5.192 | 0.364408 | 39.93 | 5.48 | -24.37 | 1.078.963 | 211.25 | 28.98 |
| 30166 | 59 | 100_6-1_L2_Glomeris_marginata | 0.929 |  | 0.929 | -5.709 | 0.364219 | 49.24 | 5.3 | -25.38 | 1.077.875 | 259.42 | 27.93 |
| 30167 | 60 | 101_6-2_A2_Glomeris_marginata | 0.559 |  | 0.559 | -5.135 | 0.364429 | 31.2 | 5.58 | -26.09 | 1.077.119 | 198.94 | 35.59 |
| 30168 | 61 | 102_8-3_L2_Glomeris_marginata | 0.746 |  | 0.746 | -5.958 | 0.364128 | 44.99 | 6.03 | -24.99 | 1.078.293 | 232.94 | 31.23 |
| 30169 | 62 | 103_5-2_L1_Allajulus_nitidus | 0.721 |  | 0.721 | -2.193 | 0.365503 | 43.53 | 6.04 | -23.62 | 1.079.763 | 206.76 | 28.68 |
| 30170 | 63 | 104_1-1_B2_Allajulus_nitidus | 0.516 |  | 0.516 | -2.242 | 0.365485 | 30.19 | 5.85 | -25.17 | 1.078.109 | 151.23 | 29.31 |
| 30173 | 66 | 106_1-3_B2_Allajulus_nitidus | 0.78 | 0.564 | 1.344 | -0.432 | 0.366145 | 51.91 | 6.65 | -23.72 | 1.079.657 | 225.03 | 28.85 |
| 30174 | 67 | 107_4-5_L2_Allajulus_nitidus | 0.881 | 0.961 | 1.842 | -4.822 | 0.364543 | 49.13 | 5.58 | -28.87 | 1.074.148 | 376.75 | 42.76 |
| 30175 | 68 | 108_5-3_L2_Allajulus_nitidus | 0.837 | 1.386 | 2.223 | -5.768 | 0.364198 | 43.34 | 5.18 | -23.1 | 1.080.315 | 211.12 | 25.22 |
| 30176 | 69 | 109_5-4_L2_Allajulus_nitidus | 0.939 | 2.437 | 3.376 | -5.09 | 0.364445 | 57.02 | 6.07 | -24 | 1.079.357 | 278.03 | 29.61 |
| 30177 | 70 | 110_5-2_A1_Cylindroiulus_punctatus | 0.641 | 8.68 | 9.321 | -2.123 | 0.365528 | 29.54 | 4.61 | -22.7 | 1.080.753 | 160.97 | 25.11 |
| 30178 | 71 | 111_6-3_A1_Cylindroiulus_punctatus | 0.882 |  | 0.882 | -4.79 | 0.364555 | 44.98 | 5.1 | -24.36 | 1.078.971 | 221.71 | 25.14 |
| 30179 | 72 | 112_8-1_L1_Cylindroiulus_punctatus | 0.611 | 8.461 | 9.072 | -3.549 | 0.365008 | 26.33 | 4.31 | -22.49 | 1.080.973 | 149.34 | 24.44 |
| 30180 | 73 | 113_8-3_A1_Cylindroiulus_punctatus | 0.722 | 0.181 | 0.903 | -6.189 | 0.364044 | 38.44 | 5.32 | -24.68 | 1.078.633 | 198.54 | 27.5 |
| 30181 | 74 | 114_5-4_A2_Cylindroiulus_punctatus | 0.678 | 0.505 | 1.183 | -4.606 | 0.364622 | 37.29 | 5.5 | -23.75 | 1.079.620 | 188.72 | 27.83 |
| 30182 | 75 | 115_6-1_L2_Cylindroiulus_punctatus | 0.96 | 18.076 | 19.036 | -3.484 | 0.365031 | 47.9 | 4.99 | -24.35 | 1.078.984 | 281.31 | 29.3 |
| 30185 | 78 | 116_6-2_A2_Cylindroiulus_punctatus | 0.858 |  | 0.858 | -2.981 | 0.365215 | 47.33 | 5.52 | -25.18 | 107.809 | 275.19 | 32.07 |
| 30186 | 79 | 117_8-2_L2_Cylindroiulus_punctatus | 0.599 | 0.364 | 0.963 | -3.069 | 0.365183 | 31.7 | 5.29 | -24.37 | 107.896 | 170.06 | 28.39 |
| 30187 | 80 | 118_5-4_L1_Cylindroiulus_caeruleocintus | 0.32 |  | 0.32 | -2.393 | 0.365429 | 16.48 | 5.15 | -22.65 | 108.08 | 85.05 | 26.58 |
| 30188 | 81 | 120_7-3_A1_Cylindroiulus_caeruleocintus | 0.209 |  | 0.209 | -0.282 | 0.3662 | 11.89 | 5.69 | -24.85 | 107.845 | 66.6 | 31.87 |
| 30189 | 82 | 121_8-1_L1_Cylindroiulus_caeruleocintus | 0.622 |  | 0.622 | -1.831 | 0.365635 | 35.61 | 5.72 | -23.37 | 108.003 | 173.88 | 27.95 |
| 30190 | 83 | 122_8-4_L1_Cylindroiulus_caeruleocintus | 0.39 |  | 0.39 | -4.149 | 0.364789 | 22.62 | 5.8 | -24.61 | 107.87 | 112.45 | 28.83 |
| 30191 | 84 | 123_5-5_L1_Brachydesmus_superus | 0.825 |  | 0.825 | -2.163 | 0.365513 | 47.92 | 5.81 | -25.29 | 107.797 | 219.33 | 26.59 |
| 30192 | 85 | 124_6-3_B1_Brachydesmus_superus | 0.26 |  | 0.26 | -3.658 | 0.364968 | 12.57 | 4.84 | -23.48 | 107.991 | 66.99 | 25.77 |
| 30193 | 86 | 125_7-4_L1_Brachydesmus_superus | 0.156 |  | 0.156 | -3.331 | 0.365087 | 8.9 | 5.71 | -24.28 | 107.905 | 46.74 | 29.96 |
| 30194 | 87 | 126_8-2_L1_Brachydesmus_superus | 0.663 |  | 0.663 | -3.095 | 0.365173 | 38.78 | 5.85 | -24.19 | 107.915 | 182.79 | 27.57 |
| 30197 | 90 | 127_4-2_A2_Brachydesmus_superus | 0.88 |  | 0.88 | -2.802 | 0.36528 | 50.78 | 5.77 | -24.68 | 107.863 | 252.06 | 28.64 |
| 30198 | 91 | 128_5-3_L2_Brachydesmus_superus | 0.777 |  | 0.777 | -3.624 | 0.36498 | 36.3 | 4.67 | -22.96 | 108.047 | 201.34 | 25.91 |
| 30199 | 92 | 129_6-2_L2_Brachydesmus_superus | 0.135 |  | 0.135 | -4.507 | 0.364658 | 7.86 | 5.82 | -23.98 | 107.937 | 41.22 | 30.54 |
| 30200 | 93 | 130_7-4_L1_Polydesmus_angustus | 0.851 | 1.502 | 2.353 | -3.696 | 0.364954 | 40.23 | 4.73 | -23.23 | 108.018 | 207.47 | 24.38 |
| 30201 | 94 | 131_8-2_A1_Polydesmus_angustus | 0.469 | 0.172 | 0.641 | -2.073 | 0.365546 | 24.52 | 5.23 | -23.12 | 108.03 | 118.62 | 25.29 |
| 30202 | 95 | 132_8-2_A1_Leptoiulus_proximus | 0.575 | 2.678 | 3.253 | -4.917 | 0.364509 | 30.92 | 5.38 | -23.63 | 107.975 | 153.43 | 26.68 |
| 30203 | 96 | 133_1-2_L1_Strigamia_acuminata | 0.948 | 0.599 | 1.547 | 2.003 | 0.367034 | 125.78 | 13.27 | -24.77 | 107.853 | 442.64 | 46.69 |
| 30204 | 97 | 135_2-1_B1_Schendyla_nemorensis | 0.449 |  | 0.449 | 2.536 | 0.367229 | 54.73 | 12.19 | -25.56 | 107.768 | 213.22 | 47.49 |
| 30205 | 98 | 136_2-3_L1_Strigamia_acuminata | 0.156 |  | 0.156 | 2.005 | 0.367035 | 20.12 | 12.9 | -24.98 | 107.831 | 74.5 | 47.76 |
| 30206 | 99 | 137_2-4_L1_Strigamia_acuminata | 0.579 | 2.503 | 3.082 | 1.65 | 0.366905 | 75.81 | 13.09 | -24.51 | 107.881 | 275.58 | 47.6 |
| 30209 | 102 | 138_3-2_L1_Strigamia_acuminata | 0.94 | 0.927 | 1.867 | 0.935 | 0.366644 | 125.65 | 13.37 | -25.19 | 107.808 | 437.62 | 46.55 |
| 30210 | 103 | 139_3-3_L1_Strigamia_acuminata | 0.757 | 1.658 | 2.415 | 0.424 | 0.366458 | 96.96 | 12.81 | -25.84 | 107.739 | 346.24 | 45.74 |
| 30211 | 104 | 140_4-1_L1_Strigamia_acuminata | 0.776 | 1.302 | 2.078 | 0.453 | 0.366468 | 101.78 | 13.12 | -26.32 | 107.688 | 354.91 | 45.74 |
| 30212 | 105 | 141_4-3_L1_Strigamia_acuminata | 0.792 | 3.642 | 4.434 | 1.071 | 0.366694 | 106.68 | 13.47 | -24.44 | 107.889 | 376.8 | 47.58 |
| 30213 | 106 | 142_4-2_L2_Strigamia_acuminata | 0.702 | 0.672 | 1.374 | 0.838 | 0.366609 | 95.76 | 13.64 | -25.27 | 107.799 | 329.27 | 46.9 |
| 30214 | 107 | 143_1-2_L1_Lithobius_curtipes | 0.275 |  | 0.275 | 1.846 | 0.366976 | 37.25 | 13.54 | -24.36 | 107.897 | 130.26 | 47.37 |
| 30215 | 108 | 144_1-4_L1_Lithobius_curtipes | 0.244 |  | 0.244 | -0.134 | 0.366254 | 33.46 | 13.71 | -25.14 | 107.814 | 117.06 | 47.98 |
| 30216 | 109 | 145_2-4_A1_Lithobius_curtipes | 0.943 | 0.509 | 1.452 | 0.068 | 0.366328 | 125.89 | 13.35 | -24.19 | 107.915 | 432.19 | 45.83 |
| 30217 | 110 | 146_3-2_A1_Lithobius_curtipes | 0.435 | 0.212 | 0.647 | 1.226 | 0.36675 | 54.47 | 12.52 | -25.18 | 107.81 | 210.99 | 48.5 |
| 30218 | 111 | 147_3-5_B1_Lithobius_curtipes | 0.615 | 0.056 | 0.671 | 1.674 | 0.366914 | 78.43 | 12.75 | -24.73 | 107.858 | 284.42 | 46.25 |
| 30221 | 114 | 148_4-1_L1_Lithobius_curtipes | 0.17 |  | 0.17 | -1.022 | 0.36593 | 23.39 | 13.76 | -25.65 | 107.759 | 85.9 | 50.53 |
| 30222 | 115 | 149_4-3_A1_Lithobius_curtipes | 0.738 | 1.172 | 1.91 | 0.68 | 0.366551 | 98.1 | 13.29 | -24.2 | 107.914 | 348.99 | 47.29 |
| 30223 | 116 | 150_8-3_L1_Lithobius_curtipes | 0.739 |  | 0.739 | -1.416 | 0.365786 | 99.91 | 13.52 | -24.14 | 107.92 | 343.72 | 46.51 |
| 30224 | 117 | 151_8-5_L1_Lithobius_curtipes | 0.551 | 1.159 | 1.71 | -2.834 | 0.365268 | 72.68 | 13.19 | -26.18 | 107.702 | 255.64 | 46.4 |
| 30225 | 118 | 152_3-3_L2_Lithobius_curtipes | 0.674 |  | 0.674 | 1.398 | 0.366813 | 89.58 | 13.29 | -24.82 | 107.848 | 305.11 | 45.27 |
| 30226 | 119 | 153_4-5_A2_Lithobius_curtipes | 0.209 |  | 0.209 | 2.597 | 0.367251 | 28.19 | 13.49 | -24.43 | 107.89 | 97.81 | 46.8 |
| 30227 | 120 | 154_6-1_L2_Lithobius_curtipes | 0.109 |  | 0.109 | 0.137 | 0.366353 | 14.44 | 13.25 | -24.94 | 107.835 | 55.17 | 50.61 |
| 30228 | 121 | 155_6-4_L2_Lithobius_curtipes | 0.781 | 0.182 | 0.963 | -1 | 0.365938 | 107.22 | 13.73 | -24.07 | 107.928 | 363.66 | 46.56 |
| 30229 | 122 | 156_8-4_L2_Lithobius_curtipes | 0.588 | 0.574 | 1.162 | -1.787 | 0.365651 | 80.38 | 13.67 | -24.59 | 107.873 | 275.55 | 46.86 |
| 30230 | 123 | 157_1-3_L1_Geophilus_flavus | 0.128 |  | 0.128 | 3.486 | 0.367575 | 15.68 | 12.25 | -24.66 | 107.865 | 63.67 | 49.75 |
| 30233 | 126 | 160_2-3_L1_Lithobius_microps | 0.269 |  | 0.269 | 1.297 | 0.366776 | 36.36 | 13.52 | -24.98 | 107.831 | 126.8 | 47.14 |
| 30234 | 127 | 161_3-2_L1_Lithobius_microps | 0.339 |  | 0.339 | 0.533 | 0.366497 | 46.51 | 13.72 | -24.67 | 107.864 | 159.89 | 47.17 |
| 30235 | 128 | 162_3-3_L1_Lithobius_microps | 0.112 |  | 0.112 | 0.547 | 0.366503 | 15.91 | 14.2 | -24.26 | 107.908 | 57.41 | 51.26 |
| 30236 | 129 | 163_5-2_A1_Lithobius_microps | 0.874 | 0.013 | 0.887 | 2.657 | 0.367273 | 118.18 | 13.52 | -23.47 | 107.993 | 412.46 | 47.19 |
| 30237 | 130 | 164_5-4_A1_Lithobius_microps | 0.207 |  | 0.207 | 0.136 | 0.366353 | 28.22 | 13.63 | -23.51 | 107.988 | 99.47 | 48.05 |
| 30238 | 131 | 165_3-2_L1_Lithobius_macilentus | 0.872 | 4.497 | 5.369 | -0.735 | 0.366035 | 116.68 | 13.38 | -26.22 | 107.698 | 412.05 | 47.25 |
| 30239 | 132 | 166_5-5_L1_Lithobius_macilentus | 0.788 | 0.871 | 1.659 | 0.978 | 0.36666 | 105.94 | 13.44 | -25.53 | 107.772 | 368.47 | 46.76 |
| 30240 | 133 | 167_2-1_A1_Geophilus_electricus | 0.846 |  | 0.846 | 0.483 | 0.366479 | 110.03 | 13.01 | -24.35 | 107.898 | 390.39 | 46.15 |
| 30241 | 134 | 168_4-3_B1_Geophilus_electricus | 0.896 | 0.444 | 1.34 | -0.782 | 0.366017 | 119.39 | 13.33 | -24.46 | 107.886 | 421.17 | 47.01 |
| 30242 | 135 | 169_2-4_A1_Lithobius_borealis | 0.948 | 0.383 | 1.331 | -0.721 | 0.36604 | 126.59 | 13.35 | -25.43 | 107.782 | 451.02 | 47.58 |
| 30245 | 138 | 170_4-1_L1_Lithobius_borealis | 0.734 | 2.772 | 3.506 | -1.617 | 0.365713 | 94.53 | 12.88 | -25.93 | 107.729 | 343.57 | 46.81 |
| 30246 | 139 | 171_4-2_L1_Lithobius_borealis | 0.622 |  | 0.622 | -0.14 | 0.366252 | 84.16 | 13.53 | -25.3 | 107.796 | 293.21 | 47.14 |
| 30247 | 140 | 172_3-3_A2_Lithobius_borealis | 0.547 |  | 0.547 | 0.869 | 0.36662 | 74.16 | 13.56 | -24.63 | 107.868 | 255.65 | 46.74 |
| 30248 | 141 | 173_4-3_A2_Lithobius_borealis | 0.87 |  | 0.87 | -0.913 | 0.36597 | 116.96 | 13.44 | -24.55 | 107.877 | 402.41 | 46.25 |
| 30249 | 142 | 174_2-4_A1_Lithobius_calcaratus | 0.654 |  | 0.654 | -0.24 | 0.366215 | 86.31 | 13.2 | -25.3 | 107.797 | 315.51 | 48.24 |
| 30250 | 143 | 175_4-3_L1_Lithobius_calcaratus | 0.941 | 2.182 | 3.123 | -0.361 | 0.366171 | 126.35 | 13.43 | -25.1 | 107.818 | 434.94 | 46.22 |
| 30251 | 144 | 176_7-4_L1_Lithobius_calcaratus | 0.997 |  | 0.997 | -0.941 | 0.365959 | 135.91 | 13.63 | -23.77 | 107.961 | 463.38 | 46.48 |
| 30252 | 145 | 177_3-1_B1_Schendyla_nemorensis | 0.391 |  | 0.391 | 2.745 | 0.367305 | 49.42 | 12.64 | -25.19 | 107.809 | 181.09 | 46.31 |
| 30253 | 146 | 178_3-2_A1_Schendyla_nemorensis | 0.386 |  | 0.386 | 1.436 | 0.366827 | 50.89 | 13.18 | -25.24 | 107.803 | 180.24 | 46.69 |
| 30254 | 147 | 180_5-1_B1_Schendyla_nemorensis | 0.115 |  | 0.115 | 2.31 | 0.367146 | 14.51 | 12.62 | -25.77 | 107.747 | 55.18 | 47.98 |
| 30267 | 6 | 313_5-1_B1_Elateridae | 0.482 |  | 0.482 | 2.028 | 0.367043 | 62.87 | 13.04 | -23.36 | 1.080.037 | 226.38 | 46.97 |
| 30268 | 7 | 314_5-2_B2_Elateridae | 0.364 |  | 0.364 | 4.2 | 0.367836 | 45.03 | 12.37 | -22.77 | 1.080.669 | 172.98 | 47.52 |
| 30269 | 8 | 315_5-3_A1_Elateridae | 0.8 | 2.317 | 3.117 | 0.138 | 0.366353 | 102.79 | 12.85 | -23.65 | 1.079.735 | 382.37 | 47.8 |
| 30270 | 9 | 316_5-4_A1_Elateridae | 0.191 |  | 0.191 | -1.443 | 0.365776 | 21.47 | 11.24 | -23.61 | 1.079.773 | 93.41 | 48.91 |
| 30271 | 10 | 317_5-5_A1_Elateridae | 0.786 | 0.866 | 1.652 | 1.94 | 0.367011 | 100.15 | 12.74 | -25.76 | 1.077.468 | 364.51 | 46.38 |
| 30272 | 11 | 318_6-1_A2_Elateridae | 0.266 |  | 0.266 | 0.92 | 0.366639 | 36 | 13.53 | -24 | 1.079.351 | 129.92 | 48.84 |
| 30273 | 12 | 320_6-3_B1_Elateridae | 0.447 |  | 0.447 | 2.882 | 0.367355 | 58.08 | 12.99 | -22.78 | 1.080.663 | 209.27 | 46.82 |
| 30274 | 13 | 321_6-4_A1_Elateridae | 0.283 |  | 0.283 | 0.675 | 0.366549 | 38.71 | 13.68 | -23.53 | 1.079.863 | 142.32 | 50.29 |
| 30275 | 14 | 322_6-5_B1_Elateridae | 0.617 |  | 0.617 | 0.458 | 0.36647 | 81.21 | 13.16 | -23.3 | 1.080.103 | 295.32 | 47.86 |
| 30276 | 15 | 323_7-1_A1_Elateridae | 0.413 |  | 0.413 | 0.121 | 0.366347 | 56.04 | 13.57 | -23.44 | 1.079.952 | 202.12 | 48.94 |
| 30279 | 18 | 324_7-2_A1_Elateridae | 0.46 |  | 0.46 | 1.5 | 0.36685 | 62.53 | 13.59 | -22.74 | 1.080.702 | 222.67 | 48.41 |
| 30280 | 19 | 325_7-3_A1_Elateridae | 0.527 |  | 0.527 | 0.156 | 0.36636 | 59.01 | 11.2 | -25.09 | 1.078.191 | 254.08 | 48.21 |
| 30281 | 20 | 326_7-4_A1_Elateridae | 0.245 |  | 0.245 | -0.425 | 0.366148 | 32.23 | 13.15 | -23.63 | 1.079.754 | 122.06 | 49.82 |
| 30282 | 21 | 327_7-5_A1_Elateridae | 0.433 |  | 0.433 | -0.759 | 0.366026 | 57.45 | 13.27 | -23.77 | 1.079.600 | 208.12 | 48.06 |
| 30283 | 22 | 328_8-1_A1_Elateridae | 0.402 |  | 0.402 | 1.073 | 0.366695 | 49.82 | 12.39 | -23.02 | 1.080.406 | 197.94 | 49.24 |
| 30284 | 23 | 329_8-2_A2_Elateridae | 0.506 |  | 0.506 | 1.021 | 0.366676 | 65.86 | 13.02 | -23.27 | 1.080.133 | 243.2 | 48.06 |
| 30285 | 24 | 330_8-3_B1_Elateridae | 0.245 |  | 0.245 | -2.904 | 0.365243 | 23.73 | 9.69 | -26.21 | 1.076.993 | 116.4 | 47.51 |
| 30286 | 25 | 331_8-4_A1_Elateridae | 0.537 |  | 0.537 | -0.971 | 0.365949 | 70.65 | 13.16 | -23.52 | 1.079.874 | 256.06 | 47.68 |
| 30287 | 26 | 332_8-5_L1_Elateridae | 1.197 | 1.183 | 2.38 | -2.433 | 0.365415 | 154.46 | 12.9 | -23.72 | 1.079.652 | 570.84 | 47.69 |
| 30288 | 27 | 339_2-2_L1_Staphylinidae | 0.164 |  | 0.164 | 1.456 | 0.366834 | 20.87 | 12.73 | -24.07 | 1.079.283 | 82.52 | 50.32 |
| 30291 | 30 | 342_2-4_A1_Staphylinidae | 0.188 |  | 0.188 | 0.551 | 0.366504 | 25 | 13.3 | -23.93 | 1.079.434 | 94 | 50 |
| 30292 | 31 | 343_2-5_L2_Staphylinidae | 0.337 |  | 0.337 | -1.259 | 0.365843 | 44.15 | 13.1 | -24.87 | 1.078.426 | 170.27 | 50.53 |
| 30293 | 32 | 344_3-1_L1_Staphylinidae | 0.844 | 0.863 | 1.707 | 1.263 | 0.366764 | 96.19 | 11.4 | -24.69 | 1.078.614 | 431.77 | 51.16 |
| 30294 | 33 | 346_3-3_L1_Staphylinidae | 0.202 |  | 0.202 | 0.467 | 0.366473 | 26.41 | 13.08 | -23.74 | 1.079.634 | 100.36 | 49.68 |
| 30295 | 34 | 350_4-2_L2_Staphylinidae | 0.111 |  | 0.111 | -2.311 | 0.36546 | 15.08 | 13.58 | -24.94 | 1.078.354 | 60.28 | 54.3 |
| 30296 | 35 | 351_4-3_L2_Staphylinidae | 0.541 | 1.469 | 2.01 | -0.472 | 0.366131 | 67.68 | 12.51 | -24.7 | 1.078.609 | 258.53 | 47.79 |
| 30297 | 36 | 353_4-5_A1_Staphylinidae | 0.681 | 0.373 | 1.054 | 1.49 | 0.366847 | 81.5 | 11.97 | -25.19 | 1.078.083 | 335.12 | 49.21 |
| 30298 | 37 | 354_4-5_A1_Aleocharinae | 0.161 |  | 0.161 | 0.934 | 0.366644 | 20.25 | 12.58 | -25.18 | 1.078.094 | 82.28 | 51.11 |
| 30299 | 38 | 355_5-1_L1_Staphylinidae | 0.219 |  | 0.219 | 0.852 | 0.366614 | 31.12 | 14.21 | -26.79 | 1.076.376 | 175.49 | 80.13 |
| 30300 | 39 | 356_5-2_L2_Staphylinidae | 0.187 |  | 0.187 | 1.144 | 0.36672 | 24.54 | 13.13 | -23.6 | 1.079.779 | 93.9 | 50.21 |
| 30303 | 42 | 360_5-5_L2_Staphylinidae | 0.271 |  | 0.271 | 2.269 | 0.367131 | 35.73 | 13.18 | -26.14 | 1.077.071 | 135.06 | 49.84 |
| 30304 | 43 | 361_6-1_L2_Staphylinidae | 1.184 | 4.944 | 6.128 | -1.212 | 0.36586 | 152.27 | 12.86 | -25.36 | 1.077.903 | 597.74 | 50.48 |
| 30305 | 44 | 363_6-2_L2_Staphylinidae | 0.656 | 1.716 | 2.372 | -1.548 | 0.365738 | 82.18 | 12.53 | -26.37 | 1.076.818 | 315.83 | 48.14 |
| 30306 | 45 | 365_6-3_L1_Staphylinidae | 0.177 |  | 0.177 | 0.112 | 0.366344 | 23.37 | 13.2 | -24.15 | 1.079.198 | 91 | 51.41 |
| 30307 | 46 | 366_6-4_L1_Staphylinidae | 0.127 |  | 0.127 | -0.847 | 0.365994 | 15.56 | 12.26 | -24.38 | 1.078.945 | 67.45 | 53.11 |
| 30308 | 47 | 368_6-5_L1_Staphylinidae | 0.41 |  | 0.41 | 0.21 | 0.36638 | 55.13 | 13.45 | -24.77 | 1.078.531 | 204.03 | 49.76 |
| 30309 | 48 | 370_7-1_B1_Staphylinidae | 0.158 |  | 0.158 | -0.06 | 0.366281 | 20.62 | 13.05 | -24.06 | 1.079.289 | 81.22 | 51.4 |
| 30310 | 49 | 371_7-2_L1_Staphylinidae | 0.28 |  | 0.28 | -2.303 | 0.365462 | 35.95 | 12.84 | -26.29 | 1.076.904 | 138.76 | 49.56 |
| 30311 | 50 | 372_7-3_L1_Staphylinidae | 0.244 |  | 0.244 | 1.631 | 0.366898 | 32.42 | 13.29 | -25.22 | 1.078.054 | 122.38 | 50.16 |
| 30312 | 51 | 373_7-4_L1_Staphylinidae | 1.029 |  | 1.029 | -0.755 | 0.366028 | 134.23 | 13.04 | -23.99 | 1.079.369 | 498.78 | 48.47 |
| 30315 | 54 | 374_7-5_L1_Staphylinidae | 0.144 |  | 0.144 | -1.592 | 0.365722 | 18.63 | 12.94 | -24.64 | 1.078.667 | 75.51 | 52.44 |
| 30316 | 55 | 379_8-3_L1_Staphylinidae | 0.217 |  | 0.217 | -1.909 | 0.365606 | 28.46 | 13.12 | -24.85 | 1.078.445 | 109.1 | 50.27 |
| 30317 | 56 | 380_8-4_L1_Staphylinidae | 0.455 |  | 0.455 | -1.582 | 0.365725 | 57.92 | 12.73 | -25.41 | 1.077.853 | 228.82 | 50.29 |
| 30318 | 57 | 381_8-5_L1_Staphylinidae | 0.4 |  | 0.4 | -0.804 | 0.366009 | 52.74 | 13.18 | -24.73 | 1.078.575 | 193.05 | 48.26 |
| 30319 | 58 | 385_1-4_L1_Dendrobaena_octaedra | 0.587 | 1.845 | 2.432 | -3.134 | 0.365159 | 45.56 | 7.76 | -25.41 | 1.077.853 | 235.61 | 40.14 |
| 30320 | 59 | 386_4-1_A1_Dendrodrilus_rubidus | 0.848 | 3.612 | 4.46 | 0.413 | 0.366454 | 117.26 | 13.83 | -24.54 | 1.078.782 | 389.15 | 45.89 |
| 30321 | 60 | 387_3-2_L1_Dendrobaena_octaedra | 1.12 | 3.522 | 4.642 | -4.623 | 0.364616 | 87.27 | 7.79 | -26.84 | 1.076.314 | 350.09 | 31.26 |
| 30322 | 61 | 388_5-3_L1_Dendrobaena_octaedra | 1.082 | 1.366 | 2.448 | -5.203 | 0.364404 | 134.01 | 12.39 | -24.25 | 1.079.088 | 483.28 | 44.67 |
| 30323 | 62 | 389_6-5_L1_Dendrobaena_octaedra | 1.478 | 3.725 | 5.203 | -5.891 | 0.364153 | 202.24 | 13.68 | -25.12 | 1.078.156 | 718.73 | 48.63 |
| 19919 | 7 | 1_1-2_L1_Acrotrichis_sp | 0.032 |  | 0.032 | -3.534 | 0.365013 | 3.6 | 11.25 | -26.61 | 1.076.631 | 15.8 | 49.37 |
| 19920 | 8 | 2_3-2_L1_Acrotrichis_sp | 0.035 |  | 0.035 | -3.864 | 0.364893 | 3.61 | 10.31 | -26.36 | 1.076.890 | 16.09 | 45.96 |
| 19921 | 9 | 3_4-1_A1_Acrotrichis_sp | 0.038 |  | 0.038 | -2.337 | 0.36545 | 4.29 | 11.28 | -26.34 | 1.076.899 | 20.54 | 54.06 |
| 19922 | 10 | 10_7-5_L1_Atheta_sp | 0.075 |  | 0.075 | -3.17 | 0.365146 | 8.85 | 11.79 | -27.55 | 1.075.574 | 43.64 | 58.18 |
| 19923 | 11 | 25_5-5_L1_Ptenidium_sp | 0.019 |  | 0.019 | 7.356 | 0.368987 | 2.23 | 11.75 | -25.19 | 1.078.117 | 11.59 | 60.99 |
| 19924 | 12 | 31_4-5_L2_Pteryx_suturalis | 0.021 |  | 0.021 | -3.375 | 0.365071 | 2.88 | 13.7 | -25.93 | 1.077.343 | 15.92 | 75.81 |
| 19925 | 13 | 32_4-4_L1_Pteryx_suturalis | 0.015 |  | 0.015 | -4.206 | 0.364768 | 1.78 | 11.88 | -24.65 | 1.078.668 | 8.12 | 54.15 |
| 19926 | 14 | 33_4-4_L1_Pselaphaulax_dresdensis | 0.043 |  | 0.043 | -0.257 | 0.366209 | 5 | 11.62 | -25.27 | 1.078.026 | 21.75 | 50.57 |
|  | 15 | 40_7-4_L1_Eusphalerum_sp | 0.08 |  | 0.08 | -6.991 | 0.363751 | 0.11 | 0.13 | -25 |  |  |  |
| 19928 | 16 | 41_6-4_L1_Eusphalerum_sp | 0.036 |  | 0.036 | 0.581 | 0.366515 | 4.36 | 12.12 | -25.83 | 1.077.443 | 18.85 | 52.35 |
| 19931 | 19 | 48_5-5_L2_Pselaphus_heisei | 0.063 |  | 0.063 | 0.74 | 0.366573 | 7.54 | 11.97 | -25.92 | 1.077.328 | 31.42 | 49.88 |
| 19932 | 20 | 50_1-5_L1_Micrambe_abietis | 0.081 |  | 0.081 | -1.134 | 0.365889 | 23.92 | 29.54 | -26.28 | 1.076.921 | 97.49 | 120.36 |
| 19933 | 21 | 59_6-2_L2_Stenichnus_sp | 0.089 |  | 0.089 | -2.262 | 0.365477 | 10.13 | 11.38 | -26.89 | 1.076.282 | 44.11 | 49.56 |
| 19934 | 22 | 60_5-2_L2_Stenichnus_sp | 0.078 |  | 0.078 | 1.968 | 0.367021 | 8.34 | 10.69 | -26.28 | 1.076.944 | 37.92 | 48.62 |
| 19935 | 23 | 105_1-2_B2_Allajulus_nitidus | 0.092 |  | 0.092 | -1.438 | 0.365778 | 8.04 | 8.74 | -25.89 | 1.077.353 | 40.03 | 43.51 |
| 19936 | 24 | 119_6-5_L1_Cylindroiulus_caeruleocintus | 0.043 |  | 0.043 | -4.389 | 0.364701 | 3.01 | 7.01 | -24.3 | 1.079.043 | 14.45 | 33.59 |
| 19937 | 25 | 134_1-3_L1_Strigamia_acuminata | 0.069 |  | 0.069 | 2.509 | 0.367218 | 8.5 | 12.32 | -25.44 | 1.077.840 | 35.45 | 51.38 |
| 19938 | 26 | 158_1-3_L1_Lithobius_microps | 0.069 |  | 0.069 | 2.846 | 0.367341 | 6.99 | 10.13 | -25.04 | 1.078.270 | 32.32 | 46.84 |
| 19939 | 27 | 159_2-2_L1_Lithobius_microps | 0.046 |  | 0.046 | 1.682 | 0.366917 | 6.35 | 13.81 | -24.53 | 1.078.818 | 22.85 | 49.68 |
| 19940 | 28 | 179_4-2_B1_Schendyla_nemorensis | 0.037 |  | 0.037 | 2.074 | 0.36706 | 4.89 | 13.21 | -25.43 | 1.077.857 | 19.01 | 51.38 |
| 19943 | 31 | 181_5-2_L1_Schendyla_nemorensis | 0.096 |  | 0.096 | 2.282 | 0.367136 | 12.54 | 13.06 | -24.45 | 1.078.911 | 44.56 | 46.41 |
| 19944 | 32 | 195_8-3_A2_Schendyla_nemorensis | 0.063 |  | 0.063 | 0.744 | 0.366574 | 8.1 | 12.86 | -24.84 | 1.078.490 | 31.8 | 50.48 |
| 19945 | 33 | 196_3-2_A1_Geophilus_truncorum | 0.055 |  | 0.055 | 2.102 | 0.36707 | 7.76 | 14.11 | -25.27 | 1.078.023 | 31.42 | 57.13 |
| 19946 | 34 | 197_8-1_B1_Geophilus_truncorum | 0.041 |  | 0.041 | 0.154 | 0.366359 | 5.22 | 12.74 | -23.13 | 1.080.292 | 19.7 | 48.04 |
| 19947 | 35 | 222_3-1_L1_Lithobius_crassipes | 0.08 |  | 0.08 | 0.167 | 0.366364 | 12.49 | 15.61 | -24.43 | 1.078.926 | 44.36 | 55.45 |
| 19948 | 36 | 231_1-1_A2_Geophilus_alpinus | 0.026 |  | 0.026 | 2.84 | 0.367339 | 3.38 | 13.01 | -25.12 | 1.078.193 | 14.42 | 55.46 |
| 19949 | 37 | 234_3-2_B2_Geophilus_ribauti | 0.046 |  | 0.046 | 2.86 | 0.367347 | 6.6 | 14.34 | -25.82 | 1.077.448 | 24.73 | 53.76 |
| 19950 | 38 | 236_8-3_L2_Agyrtidae | 0.08 |  | 0.08 | -5.256 | 0.364385 | 9.15 | 11.43 | -25.67 | 1.077.596 | 38.6 | 48.25 |
| 19951 | 39 | 238_2-2_L2_Oxyporinae | 0.03 |  | 0.03 | -0.419 | 0.36615 | 3.77 | 12.58 | -24.89 | 1.078.433 | 15.53 | 51.78 |
| 19952 | 40 | 239_8-2_L2_Cleridae | 0.034 |  | 0.034 | -1.146 | 0.365885 | 5.22 | 15.36 | -25.99 | 1.077.268 | 19.11 | 56.2 |
| 19955 | 43 | 240_2-4_L1_Cleridae | 0.085 |  | 0.085 | 0.429 | 0.366459 | 11.85 | 13.94 | -25.08 | 1.078.228 | 40.67 | 47.85 |
| 19956 | 44 | 241_6-2_L1_Cleridae | 0.046 |  | 0.046 | -1.414 | 0.365787 | 6.15 | 13.37 | -25.75 | 1.077.521 | 22.93 | 49.85 |
| 19957 | 45 | 243_4-1_A2_Carabidae | 0.026 |  | 0.026 | -1.829 | 0.365635 | 3.49 | 13.42 | -26.79 | 1.076.458 | 13.73 | 52.8 |
| 19958 | 46 | 245_6-1_A2_Scydmaenidae | 0.015 |  | 0.015 | 2.2 | 0.367106 | 1.92 | 12.82 | -24.82 | 1.078.493 | 9.38 | 62.52 |
| 19959 | 47 | 246_4-5_L2_Scydmaenidae | 0.018 |  | 0.018 | -1.078 | 0.365909 | 1.92 | 10.69 | -28.18 | 1.075.100 | 8.94 | 49.69 |
| 19960 | 48 | 247_8-5_L2_Scydmaenidae | 0.016 |  | 0.016 | -3.042 | 0.365193 | 1.76 | 11.02 | -26.92 | 1.076.397 | 7.9 | 49.4 |
| 19961 | 49 | 249_2-2_A2_Curculionidae | 0.05 |  | 0.05 | 2.812 | 0.367329 | 6.07 | 12.14 | -24.81 | 1.078.513 | 22.91 | 45.82 |
| 19962 | 50 | 252_2-2_L1_Scarabeidae | 0.094 |  | 0.094 | 1.514 | 0.366855 | 9.22 | 9.81 | -26.73 | 1.076.457 | 43.64 | 46.43 |
| 19963 | 51 | 256_1-1_L2_Cantharidae | 0.022 |  | 0.022 | -2.8 | 0.365281 | 1.79 | 8.12 | -28.77 | 1.074.460 | 10.63 | 48.34 |
| 19964 | 52 | 257_1-2_L2_Cantharidae | 0.028 |  | 0.028 | -0.756 | 0.366027 | 3.01 | 10.74 | -25.88 | 1.077.392 | 15.71 | 56.11 |
|  | 55 | 260_1-5_L2_Cantharidae | 0.079 |  | 0.079 | 3.177 | 0.367462 | 0.07 | 0.09 | -25 |  |  |  |
| 19968 | 56 | 261_2-1_A2_Cantharidae | 0.029 |  | 0.029 | -0.19 | 0.366233 | 5.13 | 17.69 | -25.32 | 1.077.980 | 20.11 | 69.34 |
| 19969 | 57 | 262_2-2_A2_Cantharidae | 0.041 |  | 0.041 | 1.265 | 0.366764 | 5.94 | 14.49 | -24.42 | 1.078.933 | 25.09 | 61.2 |
|  | 58 | 263_2-3_A2_Cantharidae | 0.05 |  | 0.05 | -14.523 | 0.361002 | 0.07 | 0.15 | -25 |  |  |  |
| 19971 | 59 | 264_2-4_B1_Cantharidae | 0.013 |  | 0.013 | -0.115 | 0.366261 | 6.24 | 47.96 | -25.05 | 1.078.259 | 26.42 | 203.24 |
| 19972 | 60 | 266_3-1_L2_Cantharidae | 0.054 |  | 0.054 | 1.085 | 0.366699 | 2.78 | 5.14 | -24.55 | 1.078.773 | 11.62 | 21.52 |
| 19973 | 61 | 267_3-2_A2_Cantharidae | 0.022 |  | 0.022 | 1.006 | 0.36667 | 4.79 | 21.75 | -25.57 | 1.077.712 | 18.94 | 86.09 |
|  | 62 | 268_3-3_A2_Cantharidae | 0.035 |  | 0.035 | -9.71 | 0.362759 | 0.07 | 0.2 | -25 |  |  |  |
| 19975 | 63 | 269_3-4_L2_Cantharidae | 0.015 |  | 0.015 | 0.443 | 0.366464 | 6.22 | 41.48 | -24.91 | 1.078.407 | 25.35 | 168.97 |
| 19976 | 64 | 270_3-5_L2_Cantharidae | 0.036 |  | 0.036 | 1.257 | 0.366762 | 4.29 | 11.91 | -25.63 | 1.077.656 | 17.13 | 47.6 |
| 19979 | 67 | 271_4-1_A2_Cantharidae | 0.033 |  | 0.033 | -1.98 | 0.36558 | 92.39 | 279.97 | -26.42 | 1.076.762 | 347.5 | 1053.02 |
| 19980 | 68 | 273_4-3_A1_Cantharidae | 0.031 |  | 0.031 | 2.018 | 0.367039 | 4.22 | 13.61 | -23.06 | 1.080.343 | 15.88 | 51.24 |
| 19981 | 69 | 274_4-4_L2_Cantharidae | 0.551 |  | 0.551 | -0.604 | 0.366082 | 61.55 | 11.17 | -25.03 | 1.078.285 | 235.81 | 42.8 |
| 19982 | 70 | 275_4-5_A1_Cantharidae | 0.022 |  | 0.022 | 2.157 | 0.36709 | 3.03 | 13.75 | -25.26 | 1.078.040 | 14.98 | 68.11 |
| 19983 | 71 | 276_5-1_L2_Cantharidae | 0.047 |  | 0.047 | 2.136 | 0.367082 | 5.69 | 12.1 | -24.95 | 1.078.364 | 23.64 | 50.3 |
| 19984 | 72 | 277_5-2_A2_Cantharidae | 0.046 |  | 0.046 | 2.876 | 0.367352 | 6.69 | 14.54 | -23.25 | 1.080.173 | 25 | 54.34 |
| 19985 | 73 | 278_5-4_A1_Cantharidae | 0.007 |  | 0.007 | 0.379 | 0.366441 | 0.76 | 10.79 | -26.53 | 1.076.878 | 4.84 | 69.1 |
| 19986 | 74 | 279_5-5_A2_Cantharidae | 0.014 |  | 0.014 | 0.906 | 0.366633 | 1.6 | 11.4 | -25.3 | 1.078.009 | 9.12 | 65.11 |
| 19987 | 75 | 280_6-1_L1_Cantharidae | 0.073 |  | 0.073 | -0.553 | 0.366101 | 8.66 | 11.86 | -23.49 | 1.079.939 | 34.79 | 47.66 |
| 19988 | 76 | 281_6-2_L2_Cantharidae | 0.028 |  | 0.028 | -1.279 | 0.365836 | 3.71 | 13.27 | -24.55 | 1.078.785 | 14.2 | 50.71 |
| 20008 | 7 | 282_6-3_L2_Cantharidae | 0.011 |  | 0.011 | 0.718 | 0.366565 | 1.6 | 14.52 | -25.45 | 1.077.876 | 6.94 | 63.05 |
| 20009 | 8 | 283_6-4_L2_Cantharidae | 0.049 |  | 0.049 | -0.62 | 0.366077 | 6.72 | 13.72 | -24.24 | 1.079.126 | 26.62 | 54.33 |
| 20010 | 9 | 284_6-5_A2_Cantharidae | 0.081 |  | 0.081 | -0.757 | 0.366027 | 9.89 | 12.21 | -23.81 | 1.079.591 | 42.36 | 52.3 |
| 20011 | 10 | 285_7-1_L2_Cantharidae | 0.026 |  | 0.026 | 0.983 | 0.366662 | 3.81 | 14.64 | -24.1 | 1.079.253 | 14.7 | 56.52 |
| 20012 | 11 | 287_7-3_A2_Cantharidae | 0.012 |  | 0.012 | 0.774 | 0.366585 | 1.72 | 14.33 | -26.71 | 1.076.579 | 9.51 | 79.22 |
| 20013 | 12 | 288_7-4_A2_Cantharidae | 0.012 |  | 0.012 | 0.089 | 0.366335 | 1.8 | 14.96 | -25.13 | 1.078.189 | 8.23 | 68.59 |
| 20014 | 13 | 290_8-1_L2_Cantharidae | 0.022 |  | 0.022 | -0.632 | 0.366072 | 3.21 | 14.58 | -23.4 | 1.079.962 | 12.29 | 55.88 |
| 20015 | 14 | 291_8-2_L1_Cantharidae | 0.022 |  | 0.022 | -0.308 | 0.366191 | 3.4 | 15.45 | -26.18 | 1.077.088 | 14.17 | 64.39 |
| 20016 | 15 | 293_8-4_L1_Cantharidae | 0.007 |  | 0.007 | 1.496 | 0.366849 | 1.41 | 20.14 | -25.25 | 1.078.067 | 6.62 | 94.57 |
| 20017 | 16 | 294_8-5_L2_Cantharidae | 0.069 |  | 0.069 | -1.383 | 0.365798 | 9.07 | 13.15 | -23.33 | 1.080.102 | 35.75 | 51.81 |
| 20020 | 19 | 304_3-2_A2_Elateridae | 0.066 |  | 0.066 | 1.995 | 0.367031 | 9.93 | 15.04 | -24.68 | 1.078.661 | 34.91 | 52.9 |
| 20021 | 20 | 309_4-2_L2_Elateridae | 0.024 |  | 0.024 | 1.989 | 0.367029 | 3.64 | 15.19 | -24.23 | 1.079.114 | 13.83 | 57.61 |
| 20022 | 21 | 319_6-2_A1_Elateridae | 0.093 |  | 0.093 | 0.705 | 0.36656 | 10.33 | 11.11 | -24.21 | 1.079.164 | 44.6 | 47.95 |
| 20023 | 22 | 333_1-1_L1_Aleocharinae | 0.033 |  | 0.033 | -0.095 | 0.366268 | 4.46 | 13.51 | -26.47 | 1.076.759 | 21.9 | 66.35 |
| 20024 | 23 | 334_1-2_L1_Staphylinidae | 0.023 |  | 0.023 | 3.621 | 0.367624 | 3.38 | 14.71 | -24.38 | 1.078.960 | 14.16 | 61.56 |
| 20025 | 24 | 335_1-3_L2_Aleocharinae | 0.014 |  | 0.014 | 0.349 | 0.36643 | 1.75 | 12.49 | -24.75 | 1.078.568 | 8.04 | 57.46 |
| 20026 | 25 | 336_1-4_L1_Aleocharinae | 0.007 |  | 0.007 | -0.89 | 0.365978 | 0.84 | 12.03 | -26.26 | 1.077.137 | 4.83 | 69 |
| 20027 | 26 | 337_1-5_L1_Aleocharinae | 0.055 |  | 0.055 | -0.347 | 0.366176 | 7.28 | 13.24 | -24.63 | 1.078.708 | 29.31 | 53.29 |
| 20028 | 27 | 338_2-1_L1_Aleocharinae | 0.04 |  | 0.04 | -0.401 | 0.366157 | 5.38 | 13.45 | -23.4 | 1.080.011 | 21.78 | 54.44 |
| 20029 | 28 | 340_2-2_L1_Aleocharinae | 0.015 |  | 0.015 | -0.666 | 0.36606 | 2.33 | 15.55 | -25.08 | 1.078.231 | 9.75 | 64.97 |
| 20032 | 31 | 341_2-3_L1_Staphylinidae | 0.089 |  | 0.089 | 1.28 | 0.36677 | 11.57 | 13 | -25.65 | 1.077.620 | 44.7 | 50.22 |
| 20033 | 32 | 345_3-2_L1_Aleocharinae | 0.04 |  | 0.04 | -1.147 | 0.365884 | 5.11 | 12.78 | -25.39 | 1.077.907 | 20.65 | 51.63 |
| 20034 | 33 | 347_3-4_L1_Aleocharinae | 0.04 |  | 0.04 | 0.111 | 0.366343 | 5.25 | 13.13 | -24.39 | 1.078.966 | 21.38 | 53.45 |
| 20035 | 34 | 348_3-5_L1_Aleocharinae | 0.059 |  | 0.059 | -0.906 | 0.365972 | 7.81 | 13.24 | -24.38 | 1.078.983 | 30.78 | 52.17 |
| 20036 | 35 | 349_4-1_A1_Staphylinidae | 0.016 |  | 0.016 | 1.726 | 0.366933 | 2.47 | 15.42 | -26.89 | 1.076.386 | 10.54 | 65.88 |
| 20037 | 36 | 352_4-4_L1_Staphylinidae | 0.067 |  | 0.067 | 1.064 | 0.366691 | 9.68 | 14.45 | -23.65 | 1.079.761 | 34.44 | 51.41 |
| 20038 | 37 | 357_5-3_L2_Staphylinidae | 0.007 |  | 0.007 | -3.06 | 0.365186 | 0.89 | 12.68 | -28.11 | 1.075.507 | 3.92 | 56.01 |
| 20039 | 38 | 358_5-3_L1_Aleocharinae | 0.032 |  | 0.032 | -1.358 | 0.365807 | 4.78 | 14.94 | -27.03 | 1.076.143 | 29.3 | 91.55 |
| 20040 | 39 | 359_5-4_L2_Staphylinidae | 0.057 |  | 0.057 | -0.798 | 0.366012 | 8 | 14.04 | -23.92 | 1.079.467 | 29.33 | 51.45 |
| 20041 | 40 | 362_6-1_L2_Aleocharinae | 0.023 |  | 0.023 | -0.152 | 0.366247 | 2.99 | 13.01 | -25.27 | 1.078.040 | 12.8 | 55.66 |
| 20044 | 43 | 364_6-2_L2_Aleocharinae | 0.044 |  | 0.044 | -2.566 | 0.365366 | 5.96 | 13.55 | -24.68 | 1.078.649 | 22.67 | 51.52 |
| 20045 | 44 | 367_6-4_L1_Aleocharinae | 0.055 |  | 0.055 | -1.914 | 0.365604 | 7.24 | 13.17 | -25.01 | 1.078.307 | 29.46 | 53.57 |
| 20046 | 45 | 369_7-1_L1_Aleocharinae | 0.082 |  | 0.082 | -1.377 | 0.3658 | 11.08 | 13.51 | -24.12 | 1.079.261 | 42.91 | 52.33 |
| 20047 | 46 | 375_7-2_L2_Aleocharinae | 0.09 |  | 0.09 | -0.689 | 0.366051 | 11.05 | 12.28 | -25.03 | 1.078.284 | 44.68 | 49.65 |
| 20048 | 47 | 376_7-5_L2_Aleocharinae | 0.031 |  | 0.031 | -3.768 | 0.364928 | 3.79 | 12.23 | -25.27 | 1.078.028 | 16.59 | 53.52 |
| 20049 | 48 | 377_8-1_L1_Aleocharinae | 0.051 |  | 0.051 | -2.175 | 0.365509 | 7.38 | 14.48 | -23.78 | 1.079.618 | 29.07 | 57 |
| 20050 | 49 | 378_8-2_L1_Staphylinidae | 0.052 |  | 0.052 | 1.135 | 0.366717 | 6.47 | 12.44 | -24.72 | 1.078.610 | 26.82 | 51.57 |
| 20051 | 50 | 382_8-4_L2_Aleocharinae | 0.023 |  | 0.023 | -2.394 | 0.365429 | 2.96 | 12.89 | -24.29 | 1.079.051 | 13 | 56.52 |
| 20052 | 51 | 383_3-5_L2_Staphylinidae | 0.071 |  | 0.071 | -0.275 | 0.366203 | 10 | 14.08 | -25.47 | 1.077.810 | 36.69 | 51.67 |
| 20053 | 52 | 384_3-2_L2_Staphylinidae | 0.025 |  | 0.025 | 0.626 | 0.366531 | 3.28 | 13.14 | -26.11 | 1.077.159 | 14.05 | 56.2 |
| 20056 | 55 | 40_7-4_L1_Eusphalerum_sp | 0.08 |  | 0.08 | -1.168 | 0.365877 | 8.66 | 10.82 | -26.93 | 1.076.241 | 41.91 | 52.39 |
| 20057 | 56 | 260_1-5_L2_Cantharidae | 0.079 |  | 0.079 | -1.088 | 0.365906 | 10.31 | 13.05 | -25.16 | 1.078.140 | 41.06 | 51.97 |
| 20058 | 57 | 263_2-3_A2_Cantharidae | 0.05 |  | 0.05 | 2.737 | 0.367302 | 1.79 | 3.58 | -24.19 | 1.079.129 | 8.9 | 17.8 |

Table S4: Stable Isotope Values for litter for each plot and layer

| id_soil | id_plot | id_quintet | id_forest | layer | Delta_C_litter | Delta_N_litter |
| --- | --- | --- | --- | --- | --- | --- |
| 1.1_A | 1.1 | 1 | 1 | A | -27.5395 | -0.2985 |
| 1.1_B | 1.1 | 1 | 1 | B | -26.6159 | 1.2729 |
| 1.1_L | 1.1 | 1 | 1 | L | -28.0701 | -4.9188 |
| 1.2_A | 1.2 | 1 | 2 | A | -28.102 | -0.4589 |
| 1.2_B | 1.2 | 1 | 2 | B | -26.4255 | 2.2083 |
| 1.2_L | 1.2 | 1 | 2 | L | -27.8829 | -4.4518 |
| 1.3_A | 1.3 | 1 | 3 | A | -27.2285 | 0.5642 |
| 1.3_B | 1.3 | 1 | 3 | B | -26.6538 | 3.39 |
| 1.3_L | 1.3 | 1 | 3 | L | -29.73 | -4.5812 |
| 1.4_A | 1.4 | 1 | 4 | A | -27.5022 | -1.7659 |
| 1.4_B | 1.4 | 1 | 4 | B | -26.3347 | 1.0557 |
| 1.4_L | 1.4 | 1 | 4 | L | -28.5813 | -5.3374 |
| 1.5_A | 1.5 | 1 | 5 | A | -25.7797 | -2.0618 |
| 1.5_B | 1.5 | 1 | 5 | B | -26.2855 | 1.1494 |
| 1.5_L | 1.5 | 1 | 5 | L | -27.8271 | -5.2832 |
| 2.1_A | 2.1 | 2 | 1 | A | -27.7973 | -1.4771 |
| 2.1_B | 2.1 | 2 | 1 | B | -27.4254 | 0.5201 |
| 2.1_L | 2.1 | 2 | 1 | L | -28.7893 | -3.7581 |
| 2.2_A | 2.2 | 2 | 2 | A | -27.3739 | -1.5572 |
| 2.2_B | 2.2 | 2 | 2 | B | -26.8924 | 1.1647 |
| 2.2_L | 2.2 | 2 | 2 | L | -28.6354 | -4.3956 |
| 2.3_A | 2.3 | 2 | 3 | A | -27.5816 | -0.451 |
| 2.3_B | 2.3 | 2 | 3 | B | -27.0514 | 2.4521 |
| 2.3_L | 2.3 | 2 | 3 | L | -29.5888 | -5.5811 |
| 2.4_A | 2.4 | 2 | 4 | A | -27.6816 | -1.113 |
| 2.4_B | 2.4 | 2 | 4 | B | -26.8392 | 1.7043 |
| 2.4_L | 2.4 | 2 | 4 | L | -29.4176 | -5.4102 |
| 2.5_A | 2.5 | 2 | 5 | A | -27.2336 | -2.7951 |
| 2.5_B | 2.5 | 2 | 5 | B | -27.1528 | 1.2327 |
| 2.5_L | 2.5 | 2 | 5 | L | -28.2506 | -6.1733 |
| 3.1_A | 3.1 | 3 | 1 | A | -27.8485 | -2.5876 |
| 3.1_B | 3.1 | 3 | 1 | B | -27.6637 | -0.472 |
| 3.1_L | 3.1 | 3 | 1 | L | -28.286 | -4.5069 |
| 3.2_A | 3.2 | 3 | 2 | A | -28.0275 | -2.0095 |
| 3.2_B | 3.2 | 3 | 2 | B | -27.7275 | 1.5555 |
| 3.2_L | 3.2 | 3 | 2 | L | -29.4589 | -4.7715 |
| 3.3_A | 3.3 | 3 | 3 | A | -27.9613 | -1.7266 |
| 3.3_B | 3.3 | 3 | 3 | B | -27.7796 | 1.0213 |
| 3.3_L | 3.3 | 3 | 3 | L | -30.029 | -5.6027 |
| 3.4_A | 3.4 | 3 | 4 | A | -27.7255 | -0.2738 |
| 3.4_B | 3.4 | 3 | 4 | B | -27.5103 | 3.6536 |
| 3.4_L | 3.4 | 3 | 4 | L | -29.7509 | -5.1576 |
| 3.5_A | 3.5 | 3 | 5 | A | -27.6975 | -2.4712 |
| 3.5_B | 3.5 | 3 | 5 | B | -27.7655 | -0.1999 |
| 3.5_L | 3.5 | 3 | 5 | L | -28.4527 | -5.7473 |
| 4.1_A | 4.1 | 4 | 1 | A | -27.5201 | -1.3305 |
| 4.1_B | 4.1 | 4 | 1 | B | -27.3495 | 0.9897 |
| 4.1_L | 4.1 | 4 | 1 | L | -28.5642 | -4.3194 |
| 4.2_A | 4.2 | 4 | 2 | A | -27.1647 | -0.8829 |
| 4.2_B | 4.2 | 4 | 2 | B | -26.8156 | 1.7571 |
| 4.2_L | 4.2 | 4 | 2 | L | -28.7636 | -4.5962 |
| 4.3_A | 4.3 | 4 | 3 | A | -27.6707 | -1.4724 |
| 4.3_B | 4.3 | 4 | 3 | B | -27.6547 | 0.0285 |
| 4.3_L | 4.3 | 4 | 3 | L | -29.6542 | -5.3088 |
| 4.4_A | 4.4 | 4 | 4 | A | -27.6404 | -1.5975 |
| 4.4_B | 4.4 | 4 | 4 | B | -27.6838 | 1.1416 |
| 4.4_L | 4.4 | 4 | 4 | L | -29.0237 | -4.7012 |
| 4.5_A | 4.5 | 4 | 5 | A | -27.249 | -0.8131 |
| 4.5_B | 4.5 | 4 | 5 | B | -27.0802 | 2.4432 |
| 4.5_L | 4.5 | 4 | 5 | L | -28.7841 | -5.2004 |
| 5.1_A | 5.1 | 5 | 1 | A | -28.0613 | -4.1562 |
| 5.1_B | 5.1 | 5 | 1 | B | -28.2999 | -1.9488 |
| 5.1_L | 5.1 | 5 | 1 | L | -28.6479 | -4.3946 |
| 5.2_A | 5.2 | 5 | 2 | A | -26.9834 | -2.401 |
| 5.2_B | 5.2 | 5 | 2 | B | -28.1068 | 0.4908 |
| 5.2_L | 5.2 | 5 | 2 | L | -28.2924 | -4.8995 |
| 5.3_A | 5.3 | 5 | 3 | A | -27.7997 | -3.6413 |
| 5.3_B | 5.3 | 5 | 3 | B | -28.1348 | -1.3852 |
| 5.3_L | 5.3 | 5 | 3 | L | -28.9307 | -6.5951 |
| 5.4_A | 5.4 | 5 | 4 | A | -28.1627 | -3.0688 |
| 5.4_B | 5.4 | 5 | 4 | B | -27.9769 | 0.4252 |
| 5.4_L | 5.4 | 5 | 4 | L | -27.7857 | -6.4812 |
| 5.5_A | 5.5 | 5 | 5 | A | -28.592 | -3.2224 |
| 5.5_B | 5.5 | 5 | 5 | B | -28.093 | -0.4605 |
| 5.5_L | 5.5 | 5 | 5 | L | -28.4655 | -4.8712 |
| 6.1_A | 6.1 | 6 | 1 | A | -27.6672 | -2.0963 |
| 6.1_B | 6.1 | 6 | 1 | B | -27.2015 | 0.2724 |
| 6.1_L | 6.1 | 6 | 1 | L | -27.9066 | -5.3162 |
| 6.2_A | 6.2 | 6 | 2 | A | -28.2515 | -2.1421 |
| 6.2_B | 6.2 | 6 | 2 | B | -27.576 | -0.2227 |
| 6.2_L | 6.2 | 6 | 2 | L | -28.5587 | -6.4072 |
| 6.3_A | 6.3 | 6 | 3 | A | -27.9067 | -2.4658 |
| 6.3_B | 6.3 | 6 | 3 | B | -27.8323 | -0.3961 |
| 6.3_L | 6.3 | 6 | 3 | L | -29.6926 | -6.3756 |
| 6.4_A | 6.4 | 6 | 4 | A | -27.9581 | -3.7953 |
| 6.4_B | 6.4 | 6 | 4 | B | -28.1616 | -0.3057 |
| 6.4_L | 6.4 | 6 | 4 | L | -29.0176 | -6.6747 |
| 6.5_A | 6.5 | 6 | 5 | A | -27.6559 | -5.1646 |
| 6.5_B | 6.5 | 6 | 5 | B | -26.9603 | -0.2036 |
| 6.5_L | 6.5 | 6 | 5 | L | -27.7827 | -7.1096 |
| 7.1_A | 7.1 | 7 | 1 | A | -28.2216 | -2.5822 |
| 7.1_B | 7.1 | 7 | 1 | B | -28.2314 | 0.7196 |
| 7.1_L | 7.1 | 7 | 1 | L | -28.4649 | -5.3594 |
| 7.2_A | 7.2 | 7 | 2 | A | -27.747 | -1.0428 |
| 7.2_B | 7.2 | 7 | 2 | B | -27.9311 | 1.9551 |
| 7.2_L | 7.2 | 7 | 2 | L | -28.5429 | -5.8716 |
| 7.3_A | 7.3 | 7 | 3 | A | -29.4595 | -2.8275 |
| 7.3_B | 7.3 | 7 | 3 | B | -27.887 | 1.5543 |
| 7.3_L | 7.3 | 7 | 3 | L | -29.7305 | -5.6634 |
| 7.4_A | 7.4 | 7 | 4 | A | -26.9852 | -3.348 |
| 7.4_B | 7.4 | 7 | 4 | B | -27.2544 | 0.3566 |
| 7.4_L | 7.4 | 7 | 4 | L | -28.0567 | -6.7712 |
| 7.5_A | 7.5 | 7 | 5 | A | -27.822 | -5.1997 |
| 7.5_B | 7.5 | 7 | 5 | B | -27.4605 | -1.637 |
| 7.5_L | 7.5 | 7 | 5 | L | -28.3298 | -8.3103 |
| 8.1_A | 8.1 | 8 | 1 | A | -27.6227 | -3.1084 |
| 8.1_B | 8.1 | 8 | 1 | B | -27.9193 | -2.6469 |
| 8.1_L | 8.1 | 8 | 1 | L | -27.7365 | -5.6055 |
| 8.2_A | 8.2 | 8 | 2 | A | -28.0218 | -2.5722 |
| 8.2_B | 8.2 | 8 | 2 | B | -27.8523 | -1.9917 |
| 8.2_L | 8.2 | 8 | 2 | L | -28.706 | -5.7262 |
| 8.3_A | 8.3 | 8 | 3 | A | -27.8975 | -4.3558 |
| 8.3_B | 8.3 | 8 | 3 | B | -27.7476 | -0.4593 |
| 8.3_L | 8.3 | 8 | 3 | L | -29.2665 | -7.5421 |
| 8.4_A | 8.4 | 8 | 4 | A | -27.7334 | -4.8683 |
| 8.4_B | 8.4 | 8 | 4 | B | -27.5049 | -2.0864 |
| 8.4_L | 8.4 | 8 | 4 | L | -28.5586 | -6.9395 |
| 8.5_A | 8.5 | 8 | 5 | A | -27.3545 | -4.2359 |
| 8.5_B | 8.5 | 8 | 5 | B | -27.5788 | -1.3343 |
| 8.5_L | 8.5 | 8 | 5 | L | -27.4697 | -6.8988 |
