# Supplementary material for: The influence of forest types including native and non‐native tree species on soil macrofauna depends on site conditions
Source: Ecol Evol. 2024 Sep 18;14(9):e70311. doi: 10.1002/ece3.70311 (PMC11410562; doi:10.1002/ece3.70311)
Supplement: Supplementary file 2 — Data S2. [file ECE3-14-e70311-s001.zip › Wenglein_et_al_R_code.docx]

####################################################################################################################

# This is the script used for my community research about

#"The influence of forest types including native and non-native tree species on

#soil macrofauna depends on site conditions"

####################################################################################################################

####-----Library------------########

library(readxl)

library(ggsignif) # for adding significant lines into plots

library(ggplot2)

library(MASS)

library(ellipse)

library(tidyverse) # collections of usefull packages, i.e. ggplot, dplyr, ...

library(tidyr)

library(dplyr)

library(ggpubr) # additional functions to combine plots nicely

library(vegan) # ordinations

library(viridis)

library(car) # Anova Type 2 + 3

library(foreign)

library(agricolae) # Tukey-HSD test

library(nlme)

library(lme4) # LMM

library(patchwork) # to combine plots

library(ggrepel) # to avoid overlapping labels

library(data.table)

library(pacman)

library(stringr)

library(rstatix)

library(kableExtra)

library(stats) # for stepwise forward selection

library(knitr)

library(devtools)

library (multcomp) # for Turkey HSD tests of GLM

library(effects)

library(emmeans)

library(nortest) # Tests for data distribution, i.e. normality

library(purrr)

library(RVAideMemoire) # Test for Permanova Stepwise

#install.packages('tidyr')

######----- Information ------###########

## my colors for forest types

# "chocolate4","chocolate2","chartreuse2","lightskyblue3", "lightskyblue4"

#### ----- Data preparation----############

rm(list=ls())

#read in the files

#List with all species and the assigned guilds by literature and 15C stable isotope data

species_guilds <- read.csv2("Data/Species_Guilds.csv")

#Full species with all

macrofauna <- read.csv2("Data/Macrofauna.csv")

## environmental factors

env <- readRDS("Data/env.rds")

mesofaunabiomass <- read.csv2("Data/mesofaunabiomass.csv")

# Drop the layers - not what I am focusing on here. I want to keep it simple

# Also aggregate the pseudo replicates to reduce the number of zeros

data <- macrofauna[,c(1,3,4,6:137)]

data <- aggregate(.~id_plot, data, sum)

# because I summed 3 layer of 2 pseudo replicates I need to divide id_quintet and id_forest by 6

data$id_quintet <- data$id_quintet / 6

data$id_forest <- data$id_forest / 6

#It is easier to work with abbreviation than numbers for forest types

data <- data %>%

mutate(data, forest_type = case_when (endsWith(id_plot, "1") ~ "Dou",

endsWith(id_plot, "2") ~ "DouBe",

endsWith(id_plot, "3") ~ "Be",

endsWith(id_plot, "4") ~ "SpBe",

endsWith(id_plot, "5") ~ "Sp"))

data$forest_type <- factor(data$forest_type,levels=c('Dou','DouBe','Be','SpBe', 'Sp'))

# region 5,6,7,8 are Sandy, regions 1,2,3,4 are loamy

data <- data %>%

mutate(region= if_else(.$id_quintet >= 5, 'Sandy', 'Loamy'))

########---------- Data long for assigning different guilds ------------------############

#Primary decomposers - feeding mainly on fresh litter

#Secondary decomposers - feeding mainly on litter colonized by fungi and microbes

#Predators - prey on other animals

### Long data format for all the species, Forest Type and Region for each plot

data_long <- gather(data, Species, Numbers, Strigamia.acuminata:Philoscia.affinis, factor_key=TRUE)

## Assigning the Guilds - Megasternum.concinnum is left out - known to be a freshwater beetle - identification error

#as well as Psephenidae, Myrmecia (1 occurence)

data_long$Guild <- species_guilds$Guild[match(data_long$Species,species_guilds$Species)]

## we use sum as we want to count how many are there in total

long_guild <- aggregate(data_long$Numbers, by=list(data_long$forest_type, data_long$region, data_long$Guild), FUN=sum)

colnames(long_guild) <- c("forest_type", "region","Guild", "abundance")

long_guild_sum <- aggregate(data_long$Numbers, by=list(data_long$Guild), FUN=sum)

summary_stats <- long_guild %>% group_by(Guild) %>% summarize(mean = mean(abundance),se = se(abundance))

## for differences in species richness for the different guilds

#long_guild <- apply(data_long$richness, by = list(data_long$forest_type, data_long$region, data_long$Guild)> 0, 1, sum )

##### Dividing into the different guilds

primary_long <- filter(data_long, Guild == "primary decomposer")

secondary_long <- filter(data_long, Guild == "secondary decomposer")

predator_long <- filter(data_long, Guild == "predator")

### back to the wide format

primary <- primary_long %>%

pivot_wider(id_cols = c(id_plot, id_quintet, forest_type, region),

names_from = "Species", values_from = "Numbers" ) #%>% select(-Guild)

secondary <- secondary_long %>%

pivot_wider(id_cols = c(id_plot, id_quintet, forest_type, region),

names_from = "Species", values_from = "Numbers" ) #%>% select(-Guild)

predator <- predator_long %>%

pivot_wider(id_cols = c(id_plot, id_quintet, forest_type, region),

names_from = "Species", values_from = "Numbers" ) #%>% select(-Guild)

### Abundance and Richness for the Groups #################

data$forest_type <- factor(data$forest_type,levels=c('Dou','DouBe','Be','SpBe', 'Sp'))

primary$forest_type <- factor(primary$forest_type,levels=c('Dou','DouBe','Be','SpBe', 'Sp'))

secondary$forest_type <- factor(secondary$forest_type,levels=c('Dou','DouBe','Be','SpBe', 'Sp'))

predator$forest_type <- factor(predator$forest_type,levels=c('Dou','DouBe','Be','SpBe', 'Sp'))

#Abundance

data$abundance <- rowSums(data[, c(4:135)])

primary$abundance <- rowSums(primary[, c(5:27)])

secondary$abundance <- rowSums(secondary[, c(5:53)])

predator$abundance <- rowSums(predator[, c(5:61)])

## for Abundance per squarmeter we need to total area they cover = 0.062 (2 cores with 20 cm diameter)

# now we multiply by x 16.13 for one m^2

data$plotabundance <-data$abundance *16.13

primary$plotabundance <-primary$abundance *16.13

secondary$plotabundance <- secondary$abundance *16.13

predator$plotabundance <- predator$abundance *16.13

#Richness without juveniles

data$species_rich <- apply(data[, c(4:10,12:119,121:125,127:135)]> 0,1, sum)

primary$species_rich <- apply(primary[, c(5:19,21:23,25:27)] > 0,1, sum)

secondary$species_rich <- apply(secondary[, c(5:53)] > 0,1, sum)

predator$species_rich <- apply(predator[, c(5:11,13:61)] > 0,1, sum)

###### Univariant Analysis for Groups ###############

se <- function(x) sd(x) / sqrt(length(x)) # Create own function

##Abundance

#All Macrofauna

all.abundance <- data %>% group_by(forest_type, region) %>%

summarize(min = min(plotabundance),median = median(plotabundance),mean = mean(plotabundance),

sd= sd(plotabundance), se =se(plotabundance), max = max(plotabundance))

#Primary

primary.abundance <- primary %>% group_by(forest_type, region) %>%

summarize(min = min(plotabundance),median = median(plotabundance),mean = mean(plotabundance),

sd= sd(plotabundance), se =se(plotabundance), max = max(plotabundance))

primary.abundance$Group <- 'Primary'

#Secondary

secondary.abundance <- secondary %>% group_by(forest_type, region) %>%

summarize(min = min(plotabundance),median = median(plotabundance),mean = mean(plotabundance),

sd= sd(plotabundance), se =se(plotabundance),max = max(plotabundance))

secondary.abundance$Group <- 'Secondary'

#Predator

predator.abundance <- predator %>% group_by(forest_type, region) %>%

summarize(min = min(plotabundance),median = median(plotabundance),mean = mean(plotabundance),

sd= sd(plotabundance), se =se(plotabundance),max = max(plotabundance))

predator.abundance$Group <- 'Predator'

guild.abundance <- rbind(primary.abundance, secondary.abundance, predator.abundance)

write.csv(guild.abundance, "Results\\Manuskript\\Univariant_guild_abundance.csv", row.names=FALSE)

## Species Richness

#All Macrofauna

all.rich <- data %>% group_by(forest_type, region) %>%

summarize(min = min(species_rich),median = median(species_rich),mean = mean(species_rich),

sd= sd(species_rich), se =se(species_rich), max = max(species_rich))

#Primary

primary.rich <- primary %>% group_by(forest_type, region) %>%

summarize(min = min(species_rich),median = median(species_rich),mean = mean(species_rich),

sd= sd(species_rich), se =se(abundance),max = max(species_rich))

primary.rich$Group <- 'Primary'

#Secondary

secondary.rich <- secondary %>% group_by(forest_type, region) %>%

summarize(min = min(species_rich),median = median(species_rich),mean = mean(species_rich),

sd= sd(species_rich), se =se(abundance),max = max(species_rich))

secondary.rich$Group <- 'Secondary'

#Predator

predator.rich <- predator %>% group_by(forest_type, region) %>%

summarize(min = min(species_rich),median = median(species_rich),mean = mean(species_rich),

sd= sd(species_rich), se =se(abundance),max = max(species_rich))

predator.rich$Group <- 'Predator'

guild.rich <- rbind(primary.rich, secondary.rich, predator.rich)

write.csv(guild.rich, "Results\\Manuskript\\Univariant_guild_richness.csv", row.names=FALSE)

###Full numbers

guild.abundance %>% group_by(Group) %>%

summarize(min = min(min),median = median(median),mean = mean(mean),

sd= sd(sd), se =se(se), max = max(max))

guild.rich %>% group_by( Group) %>%

summarize(min = min(min),median = median(median),mean = mean(mean),

sd= sd(sd), se =se(se), max = max(max))

########### Statistic Models ###################################################

#Model check function for checking residuals

model.check <- function(model = NULL, var = NULL){

par(mfrow = c(1,2))

qqnorm(resid(model)); qqline(resid(model))

plot(fitted(model), residuals(model, type = c("pearson")));abline(a=0,b=0)

par(mfrow = c(1,1))

}

### starting off with a simple anova

### to test between the guilds we use the mean for the guilds for forest_types and regions

two.way2 <- aov(mean ~ Group * forest_type + region, data = guild.abundance)

summary(two.way2)

sink("Results\\Manuskript\\anova_guilds_mean.txt")

summary(two.way2)

sink()

#### ---------Generalized Linear Mixed Models -----------------########################

###going with glmer because it fits the data better

#### full macrofauna

##Abundance

ma1a <- glmer(abundance ~ forest_type + region + (1 | id_plot), family = poisson, data = data)

ma2a <- glm(abundance ~ forest_type, family = poisson, data = data)

ma3a <- glmer(abundance ~ forest_type * region + (1 | id_quintet), family = poisson, data = data)

ma4a <- glmer(abundance ~ forest_type * region + (1 | id_plot), family = poisson, data = data)

alrt_result <- anova( ma2a, ma3a, ma1a, test = "Chisq")

ma1a.check <- model.check(ma1a)

ma2a.check <- model.check(ma2a)

ma3a.check <- model.check(ma3a) #<- best fit

ma4a.check <- model.check(ma4a)

car::Anova(ma3a, type=3)

summary(glht(ma3a, mcp(forest_type="Tukey",interaction_average = TRUE)))

summary(glht(ma3a, mcp(region="Tukey",interaction_average = TRUE)))

##species_rich

ma1r <- glmer(species_rich ~ forest_type + region + (1 | id_plot), family = poisson, data = data)

ma2r <- glm(species_rich ~ forest_type, family = poisson, data = data)

ma3r <- glmer(species_rich ~ forest_type * region + (1 | id_quintet), family = poisson, data = data)

ma4r <- glmer(species_rich ~ forest_type * region + (1 | id_plot), family = poisson, data = data)

alrt_result4 <- anova( ma2r, ma3r, ma1r, test = "Chisq")

ma1r.check <- model.check(ma1r)

ma2r.check <- model.check(ma2r)

ma3r.check <- model.check(ma3r)

ma4r.check <- model.check(ma4r) #<- best fit

car::Anova(ma4r, type=3)

summary(glht(ma4r, mcp(forest_type="Tukey",interaction_average = TRUE)))

summary(glht(ma4r, mcp(region="Tukey",interaction_average = TRUE)))

####GLMM for Guilds

#### Abundance

#Primary

m1a <- glmer(abundance ~ forest_type + region + (1 | id_plot), family = poisson, data = primary)

m2a <- glm(abundance ~ forest_type, family = poisson, data = primary)

m3a <- glmer(abundance ~ forest_type * region + (1 | id_quintet), family = poisson, data = primary)

m4a <- glmer(abundance ~ forest_type * region + (1 | id_plot), family = poisson, data = primary)

lrt_result <- anova( m2a, m3a, m1a, test = "Chisq")

m1a.check <- model.check(m1a)

m2a.check <- model.check(m2a)

m3a.check <- model.check(m3a) #<- best fit

m4a.check <- model.check(m4a)

car::Anova(m3a, type=3)

summary(glht(m3a, mcp(forest_type="Tukey",interaction_average = TRUE)))

summary(glht(m3a, mcp(region="Tukey",interaction_average = TRUE)))

##Secondary

m5a <- glmer(abundance ~ forest_type + region + (1 | id_plot), family = poisson, data = secondary)

m6a <- glm(abundance ~ forest_type, family = poisson, data = secondary)

m7a <- glmer(abundance ~ forest_type * region + (1 | id_quintet), family = poisson, data = secondary)

m8a <- glmer(abundance ~ forest_type * region + (1 | id_plot), family = poisson, data = secondary)

lrt_result2 <- anova( m5a, m6a, m7a, m8a, test = "Chisq")

m5a.check <- model.check(m5a)

m6a.check <- model.check(m6a)

m7a.check <- model.check(m7a) #<- best fit

m8a.check <- model.check(m8a)

car::Anova(m7a, type=3)

summary(glht(m7a, mcp(forest_type="Tukey",interaction_average = TRUE)))

summary(glht(m7a, mcp(region="Tukey",interaction_average = TRUE)))

##Predatory

m9a <- glmer(abundance ~ forest_type + region + (1 | id_plot), family = poisson, data = predator)

m10a <- glm(abundance ~ forest_type, family = poisson, data = predator)

m11a <- glmer(abundance ~ forest_type * region + (1 | id_quintet), family = poisson, data = predator)

m12a <- glmer(abundance ~ forest_type * region + (1 | id_plot), family = poisson, data = predator)

lrt_result3 <- anova( m9a, m10a, m11a, m12a, test = "Chisq")

m9a.check <- model.check(m9a)

m10a.check <- model.check(m10a)

m11a.check <- model.check(m11a) #<- best fit

m12a.check <- model.check(m12a)

car::Anova(m11a, type=3)

summary(glht(m11a, mcp(forest_type="Tukey",interaction_average = TRUE)))

summary(glht(m11a, mcp(region="Tukey",interaction_average = TRUE)))

#### Richness

#Primary

m1r <- glmer(species_rich ~ forest_type + region + (1 | id_plot), family = poisson, data = primary)

m2r <- glm(species_rich ~ forest_type, family = poisson, data = primary)

m3r <- glmer(species_rich ~ forest_type * region + (1 | id_quintet), family = poisson, data = primary)

m4r <- glmer(species_rich ~ forest_type * region + (1 | id_plot), family = poisson, data = primary)

lrt_result4 <- anova( m2r, m3r, m1r, test = "Chisq")

m1r.check <- model.check(m1r)

m2r.check <- model.check(m2r)

m3r.check <- model.check(m3r)

m4r.check <- model.check(m4r) #<- best fit

car::Anova(m4r, type=3)

summary(glht(m4r, mcp(forest_type="Tukey",interaction_average = TRUE)))

summary(glht(m4r, mcp(region="Tukey",interaction_average = TRUE)))

##Secondary

m5r <- glmer(species_rich ~ forest_type + region + (1 | id_plot), family = poisson, data = secondary)

m6r <- glm(species_rich ~ forest_type* region, family = poisson, data = secondary)

m7r <- glmer(species_rich ~ forest_type * region + (1 | id_quintet), family = poisson, data = secondary)

m8r <- glmer(species_rich ~ forest_type * region + (1 | id_plot), family = poisson, data = secondary)

lrt_result2 <- anova( m5r, m6r, m7r, m8r, test = "Chisq")

m5r.check <- model.check(m5r)

m6r.check <- model.check(m6r) #<- best fit and not singular

m7r.check <- model.check(m7r)

m8r.check <- model.check(m8r)

car::Anova(m6r, type=3)

summary(glht(m6r, mcp(forest_type="Tukey",interaction_average = TRUE)))

summary(glht(m6r, mcp(region="Tukey",interaction_average = TRUE)))

##Predatory

m9r <- glmer(species_rich ~ forest_type + region + (1 | id_plot), family = poisson, data = predator)

m10r <- glm(species_rich ~ forest_type, family = poisson, data = predator)

m11r <- glmer(species_rich ~ forest_type * region + (1 | id_quintet), family = poisson, data = predator)

m12r <- glmer(species_rich ~ forest_type * region + (1 | id_plot), family = poisson, data = predator)

lrt_result3 <- anova( m9r, m10r, m11r, m12r, test = "Chisq")

m9r.check <- model.check(m9r)

m10r.check <- model.check(m10r)

m11r.check <- model.check(m11r) #<- best fit

m12r.check <- model.check(m12r) #singular

car::Anova(m11r, type=3)

summary(glht(m11r, mcp(forest_type="Tukey",interaction_average = TRUE)))

summary(glht(m11r, mcp(region="Tukey",interaction_average = TRUE)))

#####-----------Abundances and Richness with Forest types and Regions Interaction --------###########################

# Full Macrofauna

pd <- position_dodge(0.2) # move them .05 to the left and right

all_abund_region_plot <- ggplot(all.abundance, aes(x=forest_type, y=mean, colour=region, group=region, fill = region)) +

geom_errorbar(aes(ymin=mean-se, ymax=mean+se), width=.1, position=pd) +

geom_line(position=pd, linetype = 2) + geom_point(position=pd, size=3, shape=21, fill="white") + # 21 is filled circle

xlab("Forest type") + ylab("Abundance (ind / m\u00B2)") + scale_colour_hue(l=20) + # Use darker colors, lightness=40

ggtitle("Total Macrofauna") +

labs(color='Region')+

scale_color_manual(values = c("#F05039","#1F449C" ))+

expand_limits(y=0) + # Expand y range

scale_y_continuous(breaks=0:10*400) + # Set tick every 4

theme(plot.title = element_text(size=1))+

theme_bw() + theme(legend.position="bottom", plot.title = element_text(size=14)) + theme(legend.text = element_text(size=14),axis.title.x = element_text(size=13), axis.text.x = element_text(angle = 45, hjust = 1, size =12)) # Position legend in bottom right

all_abund_region_plot

all_rich_region_plot <- ggplot(all.rich, aes(x=forest_type, y=mean, colour=region, group=region)) +

geom_errorbar(aes(ymin= ifelse(mean-se < 0,0, mean - se), ymax=mean+se), width=.1, position=pd) +

geom_line(position=pd, linetype = 2) + geom_point(position=pd, size=3, shape=21, fill="white") + # 21 is filled circle

xlab("Forest type") + ylab("Species richness (number of species per sample)") + scale_colour_hue(l=20) + # Use darker colors, lightness=40

ggtitle(" ") +

labs(color='Region')+

scale_color_manual(values = c("#F05039","#1F449C" ))+

expand_limits(y=0) + # Expand y range

scale_y_continuous(breaks=0:10*2) + # Set tick every 4

theme_bw() + theme(legend.position="bottom", legend.title = element_text(size=14),legend.key.size = unit(1, 'cm')) + theme(legend.text = element_text(size=14),axis.title.x = element_text(size=13), axis.text.x = element_text(angle = 45, hjust = 1, size =12)) # Position legend in bottom right

all_rich_region_plot

all_rich_regionplot <- ggarrange(all_abund_region_plot, all_rich_region_plot,

labels = c(),

ncol = 2, nrow = 1, common.legend = TRUE, legend = "bottom")

ggsave(filename = "Results/Manuskript/1Figure_all_macrofauna_abundance_richness_pdf.pdf", device = "pdf", height = 12, width = 20, units = "cm", dpi = 300)

###For the three guilds

#Abundance

pd <- position_dodge(0.2) # move them .05 to the left and right

abund_region_primary_plot <- ggplot(primary.abundance, aes(x=forest_type, y=mean, colour=region, group=region)) +

geom_errorbar(aes(ymin=mean-se, ymax=mean+se), width=.1, position=pd) +

geom_line(position=pd, linetype = 2) + geom_point(position=pd, size=3, shape=21, fill="white") + # 21 is filled circle

xlab("Forest type") + ylab("Abundance (ind / m\u00B2)") + scale_colour_hue(l=20) + # Use darker colors, lightness=40

ggtitle("Primary decomposers") +

labs(color='Region')+

scale_color_manual(values = c("#F05039","#1F449C" ))+

expand_limits(y=0) + # Expand y range

scale_y_continuous(breaks=0:10*40) + # Set tick every 4

theme(plot.title = element_text(size=1))+

theme_bw() + theme(legend.position="bottom", plot.title = element_text(size=11), axis.text.x = element_text(angle = 45, hjust = 1, size =12)) # Position legend in bottom right

abund_region_primary_plot

abund_region_secondary_plot <- ggplot(secondary.abundance, aes(x=forest_type, y=mean, colour=region, group=region)) +

geom_errorbar(aes(ymin=mean-se, ymax=mean+se), width=.1, position=pd) +

geom_line(position=pd, linetype = 2) + geom_point(position=pd, size=3, shape=21, fill="white") + # 21 is filled circle

xlab("Forest type") + ylab("Abundance (ind / m\u00B2)") + scale_colour_hue(l=20) + # Use darker colors, lightness=40

ggtitle("Secondary decomposers") +

labs(color='Region')+

scale_color_manual(values = c("#F05039","#1F449C" ))+

expand_limits(y=0) + # Expand y range

scale_y_continuous(breaks=0:12*200) + # Set tick every 4

theme(plot.title = element_text(size=8))+

theme_bw() + theme(legend.position="bottom", plot.title = element_text(size=11), axis.text.x = element_text(angle = 45, hjust = 1, size =12)) # Position legend in bottom right

abund_region_secondary_plot

abund_region_predator_plot <- ggplot(predator.abundance, aes(x=forest_type, y=mean, colour=region, group=region)) +

geom_errorbar(aes(ymin=mean-se, ymax=mean+se), width=.1, position=pd) +

geom_line(position=pd, linetype = 2) + geom_point(position=pd, size=3, shape=21, fill="white") + # 21 is filled circle

xlab("Forest type") + ylab("Abundance (ind / m\u00B2)") + scale_colour_hue(l=20) + # Use darker colors, lightness=40

ggtitle("Predators") +

labs(color='Region')+

scale_color_manual(values = c("#F05039","#1F449C" ))+

expand_limits(y=0) + # Expand y range

scale_y_continuous(breaks=0:22*100) + # Set tick every 4

theme(plot.title = element_text(size=8))+

theme_bw() + theme(legend.position="bottom", plot.title = element_text(size=11), axis.text.x = element_text(angle = 45, hjust = 1, size =12)) # Position legend in bottom right

abund_region_predator_plot

Abund_guild_regionplot <- ggarrange(abund_region_primary_plot, abund_region_secondary_plot, abund_region_predator_plot,

labels = c(),

ncol = 3, nrow = 1, common.legend = TRUE, legend = "none")

Abund_guild_regionplot

#ggsave(filename = "Results/Manuskript/Abundance_guilds_regions.png", device = "png", height = 10, width = 20, units = "cm")

### Richness Region

rich_region_primary_plot <- ggplot(primary.rich, aes(x=forest_type, y=mean, colour=region, group=region)) +

geom_errorbar(aes(ymin= ifelse(mean-se < 0,0, mean - se), ymax=mean+se), width=.1, position=pd) +

geom_line(position=pd, linetype = 2) + geom_point(position=pd, size=3, shape=21, fill="white") + # 21 is filled circle

xlab("Forest type") + ylab("Species richness (number of species per sample)") + scale_colour_hue(l=20) + # Use darker colors, lightness=40

#ggtitle("Primary Decomposer") +

scale_color_manual(values = c("#F05039","#1F449C" ))+

labs(color='Region')+

expand_limits(y=0) + # Expand y range

scale_y_continuous(breaks=0:10*2) + # Set tick every 4

theme_bw() + theme(legend.position="bottom", legend.title = element_text(size=14),legend.key.size = unit(1, 'cm')) + theme(legend.text = element_text(size=14), axis.text.x = element_text(angle = 45, hjust = 1, size =12)) # Position legend in bottom right

rich_region_primary_plot

rich_region_secondary_plot <- ggplot(secondary.rich, aes(x=forest_type, y=mean, colour=region, group=region)) +

geom_errorbar(aes(ymin= ifelse(mean-se < 0,0, mean - se), ymax=mean+se), width=.1, position=pd) +

geom_line(position=pd, linetype = 2) + geom_point(position=pd, size=3, shape=21, fill="white") + # 21 is filled circle

xlab("Forest type") + ylab("Species richness (number of species per sample)") + scale_colour_hue(l=20) + # Use darker colors, lightness=40

#ggtitle("Secondary Decomposer") +

labs(color='Region')+

scale_color_manual(values = c("#F05039","#1F449C" ))+

expand_limits(y=0) + # Expand y range

scale_y_continuous(breaks=0:20*4) + # Set tick every 4

theme_bw() + theme(legend.position="bottom", legend.title = element_text(size=14),legend.key.size = unit(1, 'cm')) + theme(legend.text = element_text(size=14), axis.text.x = element_text(angle = 45, hjust = 1, size =12)) # Position legend in bottom right

rich_region_secondary_plot

rich_region_predator_plot <- ggplot(predator.rich, aes(x=forest_type, y=mean, colour=region, group=region)) +

geom_errorbar(aes(ymin= ifelse(mean-se < 0,0, mean - se), ymax=mean+se), width=.1, position=pd) +

geom_line(position=pd, linetype = 2) + geom_point(position=pd, size=3, shape=21, fill="white") + # 21 is filled circle

xlab("Forest type") + ylab("Species richness (number of species per sample)") + scale_colour_hue(l=20) + # Use darker colors, lightness=40

#ggtitle("Predator") +

labs(color='Region')+

scale_color_manual(values = c("#F05039","#1F449C" ))+

expand_limits(y=0) + # Expand y range

scale_y_continuous(breaks=0:22*2) + # Set tick every 4

theme_bw() + theme(legend.position="bottom", legend.title = element_text(size=14), legend.key.size = unit(1, 'cm')) + theme(legend.text = element_text(size=14), axis.text.x = element_text(angle = 45, hjust = 1, size =12)) # Position legend in bottom right

rich_region_predator_plot

rich_guild_regionplot <- ggarrange(rich_region_primary_plot, rich_region_secondary_plot, rich_region_predator_plot,

labels = c(),

ncol = 3, nrow = 1, common.legend = TRUE, legend = "bottom")

rich_guild_regionplot

#ggsave(filename = "Results/Manuskript/Richness_guilds_regions.png", device = "png", height = 10, width = 20, units = "cm")

Abund_guild_regionplot /rich_guild_regionplot

ggsave(filename = "Results/Manuskript/2Guilds_abundance_richness_interaction_pdf.pdf", device = "pdf", height = 20, width = 20, units = "cm", dpi = 300)

###############--------------- NMDS ---------#########################

######## environmental factors###################

##aggregating mesofauna biomass for the plots, as I dropped the layers #######

mesofaunabiomass$id_plot <- paste(mesofaunabiomass$id_quintet, mesofaunabiomass$id_forest, sep="_")

meso <- mesofaunabiomass[,c(5:8)]

meso$WW_total_collembola <- as.numeric(meso$WW_total_collembola)

meso$WW_total_oribatidAdult <- as.numeric(meso$WW_total_oribatidAdult)

meso$WW_total_oribatidNymph <- as.numeric(meso$WW_total_oribatidNymph)

meso$id_plot <- as.factor(meso$id_plot)

meso <- aggregate(.~id_plot, meso, sum)

### other environmental factors

env1 <- env %>%

mutate(env, forest_type = case_when (endsWith(id_forest, "1") ~ "Dou",

endsWith(id_forest, "2") ~ "DouBe",

endsWith(id_forest, "3") ~ "Be",

endsWith(id_forest, "4") ~ "SpBe",

endsWith(id_forest, "5") ~ "Sp"))

env1$forest_type <- factor(env1$forest_type,levels=c('Dou','DouBe','Be','SpBe', 'Sp'))

# region north as nutrient rich and south as nutrient poor

env1 <- env1 %>% mutate(region= if_else(.$id_quintet >= 5, 'North', 'South'))

#combining mesofaunabiomass with the rest

env1 <- cbind(env1, meso[,c(2:4)])

env.variables <- env1 %>% group_by( forest_type, region) %>% summarise(across(5:15, mean))

colnames(env1)[c(5:12,15:17)] <- c("pH", "C","C/N","Water%","Gram+","Gram-","Fungi","Litter","Collembola","Oribatida","Oribatida_Nymph")

env_factors <- env1[c(5:12,15:17)]

##stepwise forward selection

#define intercept-only model

intercept_only <- lm(pH ~ 1, data=env_factors)

#define model with all predictors

env_all <- lm(pH ~ ., data=env_factors)

#perform forward stepwise regression

forward <- step(intercept_only, direction='forward', scope=formula(env_all))

#view results of forward stepwise regression

forward$anova

#view final model

forward$coefficients

## model selected pH, C, Litter, Gram+, Gram - and Oribatida

#we leave out Oribatida because they are not directed related to primary and secondary decomposers and poor prey

env_selected <- env_factors[,c(1,2,5,6,8)]

##########################NMDS#############################

####Full Community

s1 <- "Strigamia.acuminata"

s2 <- "Philoscia.affinis"

N1 <- which(colnames(data)==s1)

N2 <- which(colnames(data)==s2)

N1; N2

## keep species that occur in => 2 plots (>= 5% of 40 plots)

species.freq <- specnumber(data[,N1:N2], MARGIN=2)

X <- names(species.freq)[which(species.freq > 1)]

X # resulted in 68 taxa

sptrans <- data[,X]

fauna.dist <- vegdist(sptrans,method="bray")

## PREMANOVA

perm <- adonis2(fauna.dist~forest_type*region,data=data)

perm

#pairwise adonis

pairwise.perm.manova(fauna.dist,data$region,nperm=999)

pairwise.perm.manova(fauna.dist,data$forest_type,nperm=999)

betadisper(fauna.dist, data$forest_type, type = c("median","centroid"), bias.adjust = FALSE)

# NMDS primary

set.seed(121)

NMDS <- metaMDS(data[,X],distance = "bray", k=3)

stressplot(NMDS)

stress_NMDS <- NMDS$stress

###envfit

en = envfit(NMDS, env_selected, permutations = 999, na.rm = TRUE)

plot(NMDS)

plot(en)

en_coord_cont = as.data.frame(scores(en, "vectors")) * ordiArrowMul(en)

## coordinates for plot

site.scores <- as.data.frame(scores(NMDS, "sites"))

dff <- cbind(data[ , c(136, 137)], data[,X])

dff <- cbind(dff, site.scores)

centroids <- dff %>% group_by(forest_type) %>% select(NMDS1, NMDS2) %>% summarise_all(list(mean)) %>% rename("mean_NMDS1"="NMDS1","mean_NMDS2"="NMDS2")

dff <- dff %>% left_join(.,centroids,by=c("forest_type")) %>% mutate_at(vars(forest_type),as.factor)

species.scores <- as.data.frame(scores(NMDS, "species")) #Using the scores function from vegan to extract the species scores and convert to a data.frame

species.scores$species <- rownames(species.scores) # create a column of species, from the rownames of species.scores

head(species.scores)

### Ellipses

nmds_plot_all <- ggplot(dff, aes(x=NMDS1,y=NMDS2)) +

labs(title ='Macrofauna', subtitle = 'Stress = 0.1792') +

stat_ellipse(data = dff, geom = "polygon", aes(x=NMDS1, y=NMDS2, fill = forest_type),alpha = 0.5, level = 0.3,linetype='dotted')+

#stat_ellipse(data = dff.pr, geom = "polygon", aes(x=NMDS1, y=NMDS2, fill = region),alpha = 0.4, level = 0.3,linetype='dotted')+

geom_segment(aes(x = 0, y = 0, xend = NMDS1, yend = NMDS2),

data = en_coord_cont, arrow = arrow(length= unit(0.25, "cm")), colour = "darkblue") +

geom_text(data = en_coord_cont, aes(x = NMDS1, y = NMDS2), colour = "darkblue", hjust="outward", vjust="outward",

fontface = "bold", size = 4,label = row.names(en_coord_cont)) +

#geom_text_repel(data=species.scores,aes(x=NMDS1,y=NMDS2,label=species),alpha=0.9, size = 3, fontface =3) +

geom_point(aes(x=NMDS1,y=NMDS2, shape = region), size = 3) +

geom_label(aes(x=mean_NMDS1,y=mean_NMDS2,color=forest_type),label=dff$forest_type,fontface=2,size=4,label.size=0,alpha=0.3, label.r=unit(0.5, "lines")) +

theme_classic(base_size = 10) +

theme(panel.grid.major = element_line(color = "grey",size = 0.75,linetype = 2))+

scale_fill_manual(values=c("#760000","#00555a","chartreuse2","chocolate2","chocolate4","lightskyblue4", "lightskyblue3", '#F8766D','#00BFC4'),

name="Forest type",

breaks=c("Loamy", "Sandy","Be", "DouBe", "Dou", "SpBe", "Sp"),

labels=c("Loamy", "Sandy","Be", "DouBe", "Dou", "SpBe", "Sp"),

)+

scale_shape_manual(values=c(4, 19),

name = "Region",

labels = c("Loamy", "Sandy"))+

scale_color_manual(values=c("chartreuse2","chocolate2","chocolate4","lightskyblue4", "lightskyblue3", 'purple4','orange3'),

name="Forest type",

breaks=c("Be", "DouBe", "Dou", "SpBe", "Sp", "Loamy", "Sandy"),

labels=c("Be", "DouBe", "Dou", "SpBe", "Sp","Loamy", "Sandy"))+

theme(legend.position = "right", legend.title = element_text(size=14), legend.key.size = unit(1, 'cm')) + theme(legend.text = element_text(size=14), axis.text.x = element_text(size =13), axis.text.y = element_text(size =13),axis.title.x = element_text(size =13), axis.title.y = element_text(size =13))

nmds_plot_all

ggsave(filename = "Results/Manuskript/3NMDS_ALL_regions_Macrofauna_pdf.pdf", device = "pdf", height = 20, width = 20, units = "cm", dpi = 300)

####Primary Decomposers

sppr1 <- "Acrotrichis.sp"

sppr2 <- "Proteroiulus.fuscus"

Nopr1 <- which(colnames(primary)==sppr1)

Nopr2 <- which(colnames(primary)==sppr2)

Nopr1; Nopr2

## keep species that occur in => 2 plots (>= 5% of 40 plots)

species.freq.pr <- specnumber(primary[,Nopr1:Nopr2], MARGIN=2)

X.pr <- names(species.freq.pr)[which(species.freq.pr > 1)]

X.pr # resulted in 67 taxa

### we only keep the plots which have animals in them, as double zeros are not possible

plots.freq.pr <- specnumber(primary[,X.pr])

plots.freq.pr

Y.pr <- names(plots.freq.pr)[which(plots.freq.pr > 0)]

Y.pr

## remove rows which have zeros

primary_reduced <- primary [c(1:3,8,10, 12,13,15:17,19:20,22:28,33,34,36:39),]

write.csv(primary_reduced, "Results/Guilds/primary_NMDS_reduced.csv", row.names=FALSE)

sptranspr <- primary[c(1:3,8,10, 12,13,15:17,19:20,22:28,33,34,36:39),X.pr]

fauna.dist.pr <- vegdist(sptranspr,method="bray")

## PREMANOVA

perm.pr <- adonis2(fauna.dist.pr~forest_type*region,data=primary[c(1:3,8,10, 12,13,15:17,19:20,22:28,33,34,36:39),])

perm.pr

#pairwise adonis

pairwise.perm.manova(fauna.dist.pr,primary[c(1:3,8,10, 12,13,15:17,19:20,22:28,33,34,36:39),]$region,nperm=999)

pairwise.perm.manova(fauna.dist.pr,primary[c(1:3,8,10, 12,13,15:17,19:20,22:28,33,34,36:39),]$forest_type,nperm=999)

# NMDS primary

set.seed(121)

NMDS.pr <- metaMDS(primary[c(1:3,8,10, 12,13,15:17,19:20,22:28,33,34,36:39),X.pr],distance = "bray", k=3)

stressplot(NMDS.pr)

stress_NMDS.pr <- NMDS.pr$stress

###envfit with variables selected through stepwise forward selection

en.pr = envfit(NMDS.pr, env_selected[c(1:3,8,10,12,13,15:17,19:20,22:28,33,34,36:39),], permutations = 999, na.rm = TRUE)

plot(NMDS.pr)

plot(en.pr)

#plot(en)

en_coord_cont.pr = as.data.frame(scores(en.pr, "vectors")) * ordiArrowMul(en.pr)

## coordinates for plot

site.scores.pr <- as.data.frame(scores(NMDS.pr, "sites"))

dff.pr <- cbind(primary[ c(1:3,8,10, 12,13,15:17,19:20,22:28,33,34,36:39), c(3:4, 28,30)], primary[c(1:3,8,10, 12,13,15:17,19:20,22:28,33,34,36:39),X.pr])

dff.pr <- cbind(dff.pr, site.scores.pr)

#dff.pr <- scores(NMDS.pr) %>% cbind(primary[c(1:3,8,10, 12,13,15:17,19:20,22:28,33,34,36:39), c(3:4, 27, 28)],primary[c(1:3,8,10, 12,13,15:17,19:20,22:28,33,34,36:39),X.pr],.)

centroids.pr <- dff.pr %>% group_by(forest_type) %>% select(NMDS1, NMDS2) %>% summarise_all(list(mean)) %>% rename("mean_NMDS1"="NMDS1","mean_NMDS2"="NMDS2")

dff.pr <- dff.pr %>% left_join(.,centroids.pr,by=c("forest_type")) %>% mutate_at(vars(forest_type),as.factor)

species.scores.pr <- as.data.frame(scores(NMDS.pr, "species")) #Using the scores function from vegan to extract the species scores and convert to a data.frame

species.scores.pr$species <- rownames(species.scores.pr) # create a column of species, from the rownames of species.scores

head(species.scores.pr)

### Ellipses

dff.pr$forest_type <- factor(dff.pr$forest_type,levels=c('Dou','DouBe','Be','SpBe', 'Sp'))

nmds_plot_primary <- ggplot(dff.pr, aes(x=NMDS1,y=NMDS2)) +

labs(title ='a) Primary decomposers', subtitle = 'Stress = 0.069') +

stat_ellipse(data = dff.pr, geom = "polygon", aes(x=NMDS1, y=NMDS2, fill = region),alpha = 0.4, level = 0.3,linetype='dotted')+

geom_segment(aes(x = 0, y = 0, xend = NMDS1, yend = NMDS2),

data = en_coord_cont.pr, arrow = arrow(length = unit(0.25,"cm")), color ="darkblue") +

geom_text(data = en_coord_cont.pr, aes(x = NMDS1, y = NMDS2), colour = "darkblue", hjust="outward", vjust="outward",

fontface = "bold", size = 3, label = row.names(en_coord_cont.pr), nudge_x = 0.05, nudge_y = 0.05) +

geom_text_repel(data=species.scores.pr,aes(x=NMDS1,y=NMDS2,label=species),alpha=1, size = 3, fontface =3) +

geom_point(aes(x=NMDS1,y=NMDS2, color = forest_type),pch=21,size=2,stroke=.1) +

#geom_label_repel(aes(x=mean_NMDS1,y=mean_NMDS2,color=forest_type),label=dff.pr$forest_type,fontface=2,size=4,label.size=1,alpha=0.9, label.r=unit(0.5, "lines")) +

geom_label(aes(x=mean_NMDS1,y=mean_NMDS2,color=forest_type),label=dff.pr$forest_type,fontface=2,size=4,label.size=0,alpha=0.9, label.r=unit(0.5, "lines")) +

theme_classic(base_size = 10) +

theme(panel.grid.major = element_line(color = "grey",size = 0.75,linetype = 2))+

theme(plot.title = element_text(size=16))+

scale_fill_manual(values=c("#F05039","#1F449C"),

name ="Region",

breaks=c("Sandy", "Loamy"),

labels=c("Sandy", "Loamy"))+

scale_color_manual(values=c("chartreuse2","chocolate2","chocolate4","lightskyblue4", "lightskyblue3"),

name="Forest type",

breaks=c("Be", "DouBe", "Dou", "SpBe", "Sp"),

labels=c("Be", "DouBe", "Dou", "SpBe", "Sp"))+

theme(legend.position = "bottom", legend.title = element_text(size=14), legend.key.size = unit(1, 'cm'), legend.box="vertical", legend.margin=margin()) + theme(legend.text = element_text(size=15))

nmds_plot_primary

#ggsave(filename = "Results/Manuskript/NMDS_primary_guild_region.png", device = "png", height = 20, width = 20, units = "cm")

### Secondary Decomposers

spsc1 <- "Dasycerus.sulcatus"

spsc2 <- "Philoscia.affinis"

Nosc1 <- which(colnames(secondary)==spsc1)

Nosc2 <- which(colnames(secondary)==spsc2)

Nosc1; Nosc2

## keep species that occur in => 2 plots

species.freq.sc <- specnumber(secondary[,Nosc1:Nosc2], MARGIN=2)

X.sc <- names(species.freq.sc)[which(species.freq.sc > 1)]

X.sc # resulted in 26 taxa

## test if there are any rows without species

plots.freq.sc <- specnumber(secondary[,X.sc])

plots.freq.sc

sptranssc <- secondary[,X.sc]

fauna.dist.sc <- vegdist(sptranssc,method="bray")

## PREMANOVA

perm.sc <- adonis2(fauna.dist.sc~forest_type*region,data=secondary)

perm.sc

#pairwise adonis

pairwise.perm.manova(fauna.dist.sc,secondary$region,nperm=999)

pairwise.perm.manova(fauna.dist.sc,secondary$forest_type,nperm=999)

betadisper(fauna.dist.sc, secondary$forest_type, type = c("median","centroid"), bias.adjust = FALSE)

# NMDS primary

set.seed(121)

NMDS.sc <- metaMDS(secondary[,X.sc],distance = "bray", k=3)

stressplot(NMDS.sc)

stress_NMDS.sc <- NMDS.sc$stress

###envfit

en.sc = envfit(NMDS.sc, env_selected, permutations = 999, na.rm = TRUE)

plot(NMDS.sc)

plot(en.sc)

en_coord_cont.sc = as.data.frame(scores(en.sc, "vectors")) * ordiArrowMul(en.sc)

## coordinates for plot

site.scores.sc <- as.data.frame(scores(NMDS.sc, "sites"))

dff.sc <- cbind(secondary[ , c(3:4, 54, 56)], secondary[,X.sc])

dff.sc <- cbind(dff.sc, site.scores.sc)

#dff.sc <- scores(NMDS.sc) %>% cbind(secondary[, c(3:4, 53, 54)],secondary[,X.sc],.)

centroids.sc <- dff.sc %>% group_by(forest_type) %>% select(NMDS1, NMDS2) %>% summarise_all(list(mean)) %>% rename("mean_NMDS1"="NMDS1","mean_NMDS2"="NMDS2")

dff.sc <- dff.sc %>% left_join(.,centroids.sc,by=c("forest_type")) %>% mutate_at(vars(forest_type),as.factor)

species.scores.sc <- as.data.frame(scores(NMDS.sc, "species")) #Using the scores function from vegan to extract the species scores and convert to a data.frame

species.scores.sc$species <- rownames(species.scores.sc) # create a column of species, from the rownames of species.scores

head(species.scores.sc)

### Ellipses

nmds_plot_secondary <- ggplot(dff.sc, aes(x=NMDS1,y=NMDS2)) +

labs(title ='b) Secondary decomposers', subtitle = 'Stress = 0.1756') +

stat_ellipse(data = dff.sc, geom = "polygon", aes(x=NMDS1, y=NMDS2, fill = region),alpha = 0.4, level = 0.3,linetype='dotted')+

geom_segment(aes(x = 0, y = 0, xend = NMDS1, yend = NMDS2),

data = en_coord_cont.sc, arrow = arrow(length= unit(0.25, "cm")), colour = "darkblue") +

geom_text(data = en_coord_cont.sc, aes(x = NMDS1, y = NMDS2), colour = "darkblue", hjust="outward", vjust="outward",

fontface = "bold", size = 3,label = row.names(en_coord_cont.sc), nudge_x = -0.05, nudge_y = 0.05) +

geom_text_repel(data=species.scores.sc,aes(x=NMDS1,y=NMDS2,label=species),alpha=0.9, size = 3, fontface =3) +

geom_point(aes(x=NMDS1,y=NMDS2, color = forest_type),pch=21,size=2,stroke=.1) +

geom_label(aes(x=mean_NMDS1,y=mean_NMDS2,color=forest_type),label=dff.sc$forest_type,fontface=2,size=4,label.size=0,alpha=0.3, label.r=unit(0.5, "lines")) +

theme_classic(base_size = 10) +

theme(plot.title = element_text(size=16))+

theme(panel.grid.major = element_line(color = "grey",size = 0.75,linetype = 2))+

scale_fill_manual(values=c("#F05039","#1F449C"),

name ="Region",

breaks=c("Sandy", "Loamy"),

labels=c("Sandy", "Loamy"))+

scale_color_manual(values=c("chartreuse2","chocolate2","chocolate4","lightskyblue4", "lightskyblue3"),

name="Forest type",

breaks=c("Be", "DouBe", "Dou", "SpBe", "Sp"),

labels=c("Be", "DouBe", "Dou", "SpBe", "Sp"))+

theme(legend.position = "bottom", legend.title = element_text(size=14), legend.key.size = unit(1, 'cm'), legend.box="vertical", legend.margin=margin()) + theme(legend.text = element_text(size=15))

nmds_plot_secondary

#ggsave(filename = "Results/Manuskript/NMDS_secondary_guild_region.png", device = "png", height = 20, width = 20, units = "cm")

### Predator

sppp1 <- "Strigamia.acuminata"

sppp2 <- "Scarabaeidae"

Nopp1 <- which(colnames(predator)==sppp1)

Nopp2 <- which(colnames(predator)==sppp2)

Nopp1; Nopp2

## keep species that occur in => 2 plots #minus Geophilus alpinus as it is very different to the rest

species.freq.pp <- specnumber(predator[,Nopp1:Nopp2], MARGIN=2)

X.pp <- names(species.freq.pp)[which(species.freq.pp > 1)]

X.pp # resulted in 32 taxa

plots.freq.pp <- specnumber(predator[,X.pp])

plots.freq.pp

sptranspp <- predator[,X.pp]

fauna.dist.pp <- vegdist(sptranspp,method="bray")

## PREMANOVA

perm.pp <- adonis2(fauna.dist.pp~forest_type*region,data=predator)

perm.pp

#pairwise adonis

pairwise.perm.manova(fauna.dist.pp,predator$region,nperm=999)

pairwise.perm.manova(fauna.dist.pp,predator$forest_type,nperm=999)

# NMDS primary

set.seed(121)

NMDS.pp <- metaMDS(predator[,X.pp],distance = "bray", k=3)

stressplot(NMDS.pp)

###envfit

en.pp = envfit(NMDS.pp, env_selected, permutations = 999, na.rm = TRUE)

plot(NMDS.pp)

#plot(en)

en_coord_cont.pp = as.data.frame(scores(en.pp, "vectors")) * ordiArrowMul(en.pp)

## coordinates for plot

site.scores.pp <- as.data.frame(scores(NMDS.pp, "sites"))

dff.pp <- cbind(predator[ , c(3:4, 62, 64)], predator[,X.pp])

dff.pp <- cbind(dff.pp, site.scores.pp)

#dff.pp <- scores(NMDS.pp) %>% cbind(predator[, c(3:4, 62, 63)],predator[,X.pp],.)

centroids.pp <- dff.pp %>% group_by(forest_type) %>% select(NMDS1, NMDS2) %>% summarise_all(list(mean)) %>% rename("mean_NMDS1"="NMDS1","mean_NMDS2"="NMDS2")

dff.pp <- dff.pp %>% left_join(.,centroids.pp,by=c("forest_type")) %>% mutate_at(vars(forest_type),as.factor)

species.scores.pp <- as.data.frame(scores(NMDS.pp, "species")) #Using the scores function from vegan to extract the species scores and convert to a data.frame

species.scores.pp$species <- rownames(species.scores.pp) # create a column of species, from the rownames of species.scores

head(species.scores.pp)

### Ellipses

# Define a scaling factor for the arrows

arrow_scaling <- 0.4

nmds_plot_predator <- ggplot(dff.pp, aes(x=NMDS1,y=NMDS2)) +

labs(title ='c) Predators', subtitle = 'Stress = 0.1203') +

stat_ellipse(data = dff.pp, geom = "polygon", aes(x=NMDS1, y=NMDS2, fill = region),alpha = 0.4, level = 0.3,linetype='dotted')+

geom_segment(aes(x = 0, y = 0, xend = NMDS1, yend = NMDS2),

data = en_coord_cont.pp * arrow_scaling, arrow = arrow(length= unit(0.25, "cm")), colour = "darkblue") +

geom_text_repel(data = en_coord_cont.pp * arrow_scaling, aes(x = NMDS1, y = NMDS2), colour = "darkblue", hjust="outward", vjust="outward",

fontface = "bold", size = 3, label = row.names(en_coord_cont.pp), nudge_x = 0.05, nudge_y = 0.05) +

geom_text_repel(data=species.scores.pp,aes(x=NMDS1,y=NMDS2,label=species),alpha=0.9, size = 3, fontface =3) +

geom_point(aes(x=NMDS1,y=NMDS2, color = forest_type),pch=21,size=2,stroke=.1) +

geom_label(aes(x=mean_NMDS1,y=mean_NMDS2,color=forest_type),label=dff.pp$forest_type,fontface=2,size=4,label.size=0,alpha=0.3, label.r=unit(0.5, "lines")) +

theme_classic(base_size = 10) +

theme(plot.title = element_text(size=16))+

theme(panel.grid.major = element_line(color = "grey",size = 0.75,linetype = 2))+

scale_fill_manual(values=c("#F05039","#1F449C"),

name ="Region",

breaks=c("Sandy", "Loamy"),

labels=c("Sandy", "Loamy"))+

scale_color_manual(values=c("chartreuse2","chocolate2","chocolate4","lightskyblue4", "lightskyblue3"),

name="Forest type",

breaks=c("Be", "DouBe", "Dou", "SpBe", "Sp"),

labels=c("Be", "DouBe", "Dou", "SpBe", "Sp"))+

coord_cartesian(ylim = c(-0.5, 0.7),

xlim = c(-1.5, 1))+

theme(legend.position = "bottom", legend.title = element_text(size=14), legend.key.size = unit(1, 'cm'), legend.box="vertical", legend.margin=margin()) + theme(legend.text = element_text(size=15))

nmds_plot_predator

#ggsave(filename = "Results/Manuskript/NMDS_predator_enfit_selected.png", device = "png", height = 20, width = 20, units = "cm")

NMDS_guild_plot <- ggarrange(nmds_plot_primary, nmds_plot_secondary, nmds_plot_predator,

labels = c(),

ncol = 3, nrow = 1, common.legend = TRUE, legend = "bottom" )

NMDS_guild_plot

ggsave(filename = "Results/Manuskript/4NMDS_guilds_enfit_selected_pdf.pdf", device = "pdf", height = 15, width = 25, units = "cm", dpi = 300)

##########################################################################################

############################## END OF SCRIPT #########################################

##################################################################################

####################################################################################################################

# This script was kindly provided by Dr. Anton Potapov, potapov.msu@gmail.com

# I adapted it to use it at my research about "The influence of forest types including native and non-native tree

# species on soil macrofauna depends on site conditions"

#

#

# before starting run the following command to clear the work space every time you analyse a different target group.

#between regions and forest types, there need to be some adjustments throughout the script

rm(list=ls())

#

# The custom functions used here, are from the appendix of Coucherousset

#

# YOU NEED TO RUN FIRST THE WHOLE SCRIPT in the file si_div.R before going further – after this script

# sourcing the R functions from 'si_div' R script

source("Code/si_div.R") # NB: several R packages are autmoatically loaded

#

####################################################################################################################

# Install, load packages and data

list.of.packages <- c("dplyr",

"vegan",

"ggpubr",

"agricolae",

"reshape2",

"ggrepel",

"plyr",

"ggplot2",

"SIBER",

"tidyverse",

"effects",

"MASS",

"tidyr")

lapply(list.of.packages, require, character.only = TRUE)

library(nlme)

library(lme4)

library(plyr)

library(dplyr)

library(MASS)

library(reshape2)

library(reshape)

# load data, for the animals and for the litter

animals <- read.csv2("Data/animals2.csv")

litter <- read.csv2("Data/litter.csv")

###################################################################################################################

#

#Splitting all the information in the Sample column

animals <- animals %>% separate(Sample, sep = "_" ,c('sample_id', 'plot_id', 'layer', 'species1', 'species2'), remove = FALSE)

animals <- animals %>% separate(plot_id, sep = "-", c('id_quintet', 'id_forest'), remove = FALSE)

### Adding animal Group

animals$totalweight <- as.numeric(animals$totalweight)

animals$sample_id <- as.numeric(animals$sample_id)

animals <- animals %>% mutate(Group = case_when(sample_id <= 60 ~ "Coleoptera",

sample_id <= 88 ~ "Isopoda",

sample_id <= 132 ~ "Diplopoda",

sample_id <= 234 ~ "Chilopoda",

sample_id <= 384 ~ "Larvae",

sample_id <= 389 ~ "Lumbricidae"))

### removing the numbers from the layers

animals$layer<-gsub("1","2",as.character(animals$layer))

animals$layer<-gsub("2","",as.character(animals$layer))

animals$id_plot <- paste(animals$id_quintet, animals$id_forest, sep=".")

animals$id_soil <- paste(animals$id_plot, animals$layer, sep="_")

#### species names

animals$Species <- paste(animals$species1, animals$species2, sep= "_")

###### making nice names for forest types

animals <- animals %>% mutate(animals, forest_type = case_when (endsWith(id_forest, "1") ~ "Dou",

endsWith(id_forest, "2") ~ "DouBe",

endsWith(id_forest, "3") ~ "Be",

endsWith(id_forest, "4") ~ "SprBe",

endsWith(id_forest, "5") ~ "Spr"))

###### Regions

animals <- animals %>% mutate(region= if_else(.$id_quintet >= 5, 'Sandy', 'Loamy'))

#### Guilds

# The data contains guilds, which are selected after the calibrated stable isotope data provided

# it is already in the data sheet

##################################################################################################################

# Now we calibrate against the litter for the different plots, for that we need id_soil

animals$id_plot <- paste(animals$id_quintet, animals$id_forest, sep=".")

animals$id_soil <- paste(animals$id_plot, animals$layer, sep="_")

### Litter correction

colnames(litter) <- paste("litter", colnames(litter), sep = "_")

litter$litter_Delta_C_litter <- as.numeric(litter$litter_Delta_C_litter)

litter$litter_Delta_N_litter <- as.numeric(litter$litter_Delta_N_litter)

animals$delta.15N <- as.numeric(animals$delta.15N)

animals$delta.13C <- as.numeric(animals$delta.13C)

####### Getting litter data for delta13C and delta 15N in the animal data frame

animals$delta13Clitter <- litter$litter_Delta_C_litter[match(animals$id_soil, litter$litter_id_soil)]

animals$delta15Nlitter <- litter$litter_Delta_N_litter[match(animals$id_soil, litter$litter_id_soil)]

animals$d13C <- (animals$delta.13C - animals$delta13Clitter)

animals$d15N <- (animals$delta.15N - animals$delta15Nlitter)

###################################################################################################

#Mean values of Delta 13C and 15N for the species

univariant_species <- animals %>% group_by(Species) %>%

summarize(mean15N = mean(d15N), sd15N = sd(d15N), mean13C =mean(d13C), sd13C = sd(d13C))

write.csv(univariant_species,"Guilds\\Manuskript\\Animalunivariant.csv", row.names = FALSE)

#################################################################################################

# Splitting the data into the different groups

#Because then we have too little samples we are going to add another column: region_forest

#for Decomposer this is still too little data so we are going to work with either region or forest type

animals$region_forest <- paste(animals$region, animals$id_forest, sep= "_")

animals$forest_type <- factor(animals$forest_type,levels=c('Dou','DouBe','Be','SprBe', 'Spr'))

# all the groups

primary <- subset(animals, Guild %in% c("primary"))

secondary <- subset(animals, Guild %in% c("secondary"))

predator <- subset(animals, Guild %in% c("predator"))

###################################################################################################

### Selecting the important columns for our data

### for forest type as system

#mydata <- animals [,c(14,5,28,32,33)] # for full macrofauna we use plots

#mydata <- predator [,c(14,34,28,32,33)]

#mydata <- secondary [,c(14,34,28,32,33)]

#I remove the two rows of South_3 because the analysis needs 3 points to work

#mydata <- primary [,c(14,34,28,32,33)]

#mydata <- mydata [c(1:11,13:26,28:65),]

### for region as system

mydata <- animals [,c(14,5,29,32,33)]

#mydata <- predator [,c(14,34,29,32,33)]

#mydata <- secondary [,c(14,34,29,32,33)]

#I remove the two rows of South_3 because the analysis needs 3 points to work

#mydata <- primary [,c(14,34,29,32,33)]

#mydata <- mydata [c(1:11,13:26,28:65),]

table(mydata$region_forest)

table(mydata$plot_id)

names(mydata)[1]<- 'weight' # dry weight from isotope analysis - maybe find some other way

names(mydata)[2]<- 'group' # id_plot, with one pseudo replicate inside

names(mydata)[3]<- 'forest' # the system we want to have a look at

names(mydata)[4]<- 'd13C' # d13 calibrated by leaf litter

names(mydata)[5]<- 'd15N' # d15 calibrated by the leaf litter

mydata$nm_plot <- paste('plot',mydata$group) # this is for pictures names

str(mydata) # check if all values are numeric

mydata <- na.omit(mydata) # remove NAs

####################################################################################################################

# Preparation for graphs

find_hull <- function(df) df[chull(df$d13C, df$d15N), ] # Just for Convex hulls calculation

Sys.setlocale(category="LC_ALL", locale="en_US.UTF-8") # Just for isotopic axes to displayed correctly

# generic graphical functions used in 'IDiversity' and 'IOverlap'

# axis titles for the 4 types of istopes used

tit_d13C<-expression(bold(paste(delta^"13",C,sep=""))) ; scl_tit_d13C<-expression(bold(paste("Scaled ",delta^"13",C,sep="")))

tit_d15N<-expression(bold(paste(delta^"15",N,sep=""))) ; scl_tit_d15N<-expression(bold(paste("Scaled ",delta^"15",N,sep="")))

# graphic function to plot isotopic space

isotopic_space<-function(nmX="X",nmY="Y", limX=c(-0.05,1.05),limY=c(-0.05,1.05),

labX=c(0,0.25,0.5,0.75,1),labY=c(0,0.25,0.5,0.75,1) ) {

# setting graphical parameters

par(mar=c(4,4.5,4,3.5)) ; tick=-0.4 ; lasX=1 ;lasY=1 ; lineX=-0.2 ; lineY=-0.2 ; cexX=0.9 ; cexY=0.9 ;

lineXt=lineX+2.1 ; lineYt=lineY+2.2 ; cexXt=1 ; cexYt=1

# empty window

plot(limX,limY,type="n",axes=F,xaxt="n",yaxt="n",xlab="",ylab="",xlim=limX,ylim=limY)

rect(limX[1],limY[1],limX[2],limY[2]) # border

# X axis

axis(side=1, at=labX, labels=F, tcl=tick, pos=limY[1]) # X thicks

mtext(side=1, labX, at=labX, line=lineX, cex=cexX, las=lasX) # X labels

mtext(side=1,nmX,cex=cexXt,line=lineXt,font=2) # X title

# Y axis

axis(side=2, at=labY, labels=F, tcl=tick, pos=limX[1]) # Y thicks

mtext(side=2, labY, at=labY, line=lineY, cex=cexY, las=lasY) # Y labels

mtext(side=2,nmY,cex=cexYt,line=lineYt,font=2) # Y title

} # end of isotopic_space

# function to add vertical and horizontal error bars

meansexy<-function(meanxy,sexy,colb="black",lg=0.1) {

segments(meanxy[,1]-sexy[,1],meanxy[,2],meanxy[,1]+sexy[,1],meanxy[,2],col=colb) # x error bar

segments(meanxy[,1],meanxy[,2]-sexy[,2],meanxy[,1],meanxy[,2]+sexy[,2],col=colb) # y error bar

segments(meanxy[,1]-sexy[,1],meanxy[,2]-lg,meanxy[,1]-sexy[,1],meanxy[,2]+lg,col=colb)

segments(meanxy[,1]+sexy[,1],meanxy[,2]-lg,meanxy[,1]+sexy[,1],meanxy[,2]+lg,col=colb)

segments(meanxy[,1]-lg,meanxy[,2]-sexy[,2],meanxy[,1]+lg,meanxy[,2]-sexy[,2],col=colb)

segments(meanxy[,1]-lg,meanxy[,2]+sexy[,2],meanxy[,1]+lg,meanxy[,2]+sexy[,2],col=colb)

} # end of meansexy

#

# End of preparation for graphs

####################################################################################################################

# Scale the data between 0 and 1 in each community for d13C and d15N so you can calculate multidimensional indexes

# To avoid bias of high variability of isotopic background in different forest types leaf litter calibrated

mydata_scaled <- scaleSI_range01(mydata) # scaling the data

table(mydata_scaled$group)

#############################################################################################################

# FOURTH calculate multidimensional indexes, I run a loop to have it for each of the plots

out <- as.data.frame(matrix(ncol=15,nrow=0)) # This is the loop

for(i in 1:length(unique(mydata_scaled$group))) { # This is the loop

loop <- subset(mydata_scaled, group==unique(mydata_scaled$group)[i])

index <- IDiversity(loop,nm_plot=loop$nm_plot[1],weight=loop$weight,scaled=T)

names(out) <- c("forest","plot",names(index))

out[i,] <- c(as.character(loop$forest[1]),as.character(loop$group[1]),index) }

# OUTPUTS: - a vector with minimum, maximum, range and abundance-weighted mean values for each isotopic axis

# (e.g. 'min_d13C', 'max_d13C', 'range_d13C', 'IPos_d13C' for d13C)

# and 5 multidimensional indices: isotopic richness ('IRic'), isotopic evenness ('IEve')

# and isotopic divergence ('IDiv'), isotopic dispersion ('IDis') and isotopic uniqueness ('IUni')

# This is the final dataset containing all metrics (usefull for multidimensional)

result_metrics <- out

#write.csv(result_metrics,'Guilds/Manuskript/result_metrics_Full_forest.csv')

#write.csv(result_metrics,'Guilds/Manuskript/result_metrics_Primary_forest.csv')

#write.csv(result_metrics,'Guilds/Manuskript/result_metrics_Secondary_forest.csv')

#write.csv(result_metrics,'Guilds/Manuskript/result_metrics_Predator_forest.csv')

write.csv(result_metrics,'Guilds/Manuskript/Region/result_metrics_All_region.csv')

#write.csv(result_metrics,'Guilds/Manuskript/region/result_metrics_Primary_region.csv')

#write.csv(result_metrics,'Guilds/Manuskript/Region/result_metrics_Secondary_region.csv')

#write.csv(result_metrics,'Guilds/Manuskript/Region/result_metrics_Predator_region.csv')

######mean for the different forest types

result_metrics_num <- result_metrics[,c(3:15)]

chars <- sapply(result_metrics_num, is.character)

#convert all character columns to numeric

result_metrics_num[ , chars] <- as.data.frame(apply(result_metrics_num[ , chars], 2, as.numeric))

sapply(result_metrics_num, class)

result_metrics2 <- cbind(result_metrics[,c(1:2)], result_metrics_num)

sapply(result_metrics2, class)

result.metrics_all_mean <- result_metrics2 %>% group_by(forest) %>%

summarise_all(funs(median= median, mean = mean, sd = sd))

#write.csv(result.metrics_all_mean,'Guilds/Manuskript/result_metrics_plots_All_mean.csv')

#write.csv(result.metrics_all_mean,'Guilds/Manuskript/result_metrics_plots_Primary_mean.csv')

#write.csv(result.metrics_all_mean,'Guilds/Manuskript/result_metrics_plots_Secondary_mean.csv')

#write.csv(result.metrics_all_mean,'Guilds/Manuskript/result_metrics_plots_Predator_mean.csv')

write.csv(result.metrics_all_mean,'Guilds/Manuskript/Region/result_metrics_plots_All_region_mean.csv')

#write.csv(result.metrics_all_mean,'Guilds/Manuskript/region/result_metrics_plots_Primary_region_mean.csv')

#write.csv(result.metrics_all_mean,'Guilds/Manuskript/region/result_metrics_plots_Secondary_region_mean.csv')

#write.csv(result.metrics_all_mean,'Guilds/Manuskript/region/result_metrics_plots_Predator_region_mean.csv')

# ALSO see plots in the "files" section

##########################################################################################

# FOURTH calculate unidimensional indexes, I run a loop to have it for each of the plots

###with unscaled data

out3 <- as.data.frame(matrix(ncol=15,nrow=0)) # This is the loop

for(i in 1:length(unique(mydata$group))) { # This is the loop

loop3 <- subset(mydata, group==unique(mydata$group)[i])

index3 <- IDiversity(loop3,nm_plot=loop3$nm_plot[1],weight=loop3$weight)

names(out3) <- c("forest","plot",names(index3))

out3[i,] <- c(as.character(loop3$forest[1]),as.character(loop3$group[1]),index3) }

# OUTPUTS: - a vector with minimum, maximum, range and abundance-weighted mean values for each isotopic axis

# (e.g. 'min_d13C', 'max_d13C', 'range_d13C', 'IPos_d13C' for d13C)

# and 5 multidimensional indices: isotopic richness ('IRic'), isotopic evenness ('IEve')

# and isotopic divergence ('IDiv'), isotopic dispersion ('IDis') and isotopic uniqueness ('IUni')

# This is the final dataset containing all metrics (usefull for multidimensional)

result_metrics_unscaled <- out3

#write.csv(result_metrics_unscaled,'Guilds/Manuskript/result_metrics_All_forest.csv')

#write.csv(result_metrics_unscaled,'Guilds/Manuskript/result_metrics_Primary_forest.csv')

#write.csv(result_metrics_unscaled,'Guilds/Manuskript/result_metrics_Secondary_forest.csv')

#write.csv(result_metrics_unscaled,'Guilds/Manuskript/result_metrics_Predator_forest.csv')

write.csv(result_metrics_unscaled,'Guilds/Manuskript/Region/result_metrics_All_region.csv')

#write.csv(result_metrics_unscaled,'Guilds/Manuskript/Region/result_metrics_Primary_region.csv')

#write.csv(result_metrics_unscaled,'Guilds/Manuskript/Region/result_metrics_Secondary_region.csv')

#write.csv(result_metrics_unscaled,'Guilds/Manuskript/Region/result_metrics_Predator_region.csv')

######mean for the different forest types

result_metrics_num_unscaled <- result_metrics_unscaled[,c(3:15)]

chars1 <- sapply(result_metrics_num_unscaled, is.character)

#convert all character columns to numeric

result_metrics_num_unscaled[ , chars1] <- as.data.frame(apply(result_metrics_num_unscaled[ , chars1], 2, as.numeric))

sapply(result_metrics_num_unscaled, class)

result_metrics_unscaled2 <- cbind(result_metrics_unscaled[,c(1:2)], result_metrics_num_unscaled)

sapply(result_metrics_unscaled2, class)

result.metrics_all_mean_unscaled <- result_metrics_unscaled2 %>% group_by(forest) %>%

summarise_all(funs(median= median, mean = mean, sd = sd))

#write.csv(result.metrics_all_mean_unscaled,'Guilds/Manuskript/result_metrics_plots_All_mean_unscaled.csv')

#write.csv(result.metrics_all_mean_unscaled,'Guilds/Manuskript/result_metrics_plots_Primary_mean_unscaled.csv')

#write.csv(result.metrics_all_mean_unscaled,'Guilds/Manuskript/result_metrics_plots_Secondary_mean_unscaled.csv')

#write.csv(result.metrics_all_mean_unscaled,'Guilds/Manuskript/result_metrics_plots_Predator_mean_unscaled.csv')

write.csv(result.metrics_all_mean_unscaled,'Guilds/Manuskript/Region/result_metrics_plots_All_region_mean_unscaled.csv')

#write.csv(result.metrics_all_mean_unscaled,'Guilds/Manuskript/Region/result_metrics_plots_Primary_region_mean_unscaled.csv')

#write.csv(result.metrics_all_mean_unscaled,'Guilds/Manuskript/Region/result_metrics_plots_Secondary_region_mean_unscaled.csv')

#write.csv(result.metrics_all_mean_unscaled,'Guilds/Manuskript/Region/result_metrics_plots_Predator_region_mean_unscaled.csv')

# ALSO see plots in the "files" section

#############################################################################################################################

##### HERE YOU CAN RUN TESTS - all metrics depending on the system

result_metrics_melted <- melt(result_metrics,id.vars = c('forest','plot')) # long-table format

result_metrics_melted$value <- as.numeric(result_metrics_melted$value)

str(result_metrics_melted)

# value should be numeric!!! if not, use the next string below: USE ONLY IF THE VALUE IS FACTOR!!!!!

# result_metrics_melted$value <- as.numeric(levels(result_metrics_melted$value))[result_metrics_melted$value]

### check the data distribution:

ggplot(result_metrics_melted,aes(x=value))+

geom_histogram()+

facet_wrap(~variable,scales='free')

### in most of the cases, the data distributed close to normal so we can apply parametric HSD test to see differences

### Here I run loop to test differences between systems in each of the metrics using post-hoc HSD.test

# To add region effect, we create a column

#####------- Only for full community

result_metrics_melted <- result_metrics_melted %>% separate(plot, sep = "-", c('region', 'id_forest'), remove = FALSE)

result_metrics_melted <- result_metrics_melted %>% mutate(region= if_else(.$region >= 5, 'Sandy', 'Loamy'))

result_metrics_melted <- result_metrics_melted %>% mutate(id_forest = case_when (endsWith(id_forest, "1") ~ "Dou",

endsWith(id_forest, "2") ~ "DouBe",

endsWith(id_forest, "3") ~ "Be",

endsWith(id_forest, "4") ~ "SprBe",

endsWith(id_forest, "5") ~ "Spr"))

### for Guilds

#result_metrics_melted <- result_metrics_melted %>% separate(plot, sep = "_", c('region', 'id_forest'), remove = FALSE)

#result_metrics_melted$id_plot <- paste(animals$id_quintet, animals$id_forest, sep=".")

#result_metrics_melted$quintet <- substr(result_metrics_melted$plot,1,1)

#result_metrics_melted <- result_metrics_melted %>% mutate(region= if_else(.$quintet = "N", 'North', 'South'))

#######----------- For groups use this

#result_metrics_melted$region <- substr(result_metrics_melted$plot,1,5)

out <- as.data.frame(matrix(ncol=4,nrow=0))

out_anova <- as.data.frame(matrix(ncol=6,nrow=0)) #### for anova

for(i in 1:length(unique(result_metrics_melted$variable))){ #### THIS IS THE LOOP

loop <- subset(result_metrics_melted, variable==unique(result_metrics_melted$variable)[i])

model <- lm(value~forest+region,loop)

posthoc <- HSD.test(model, "forest", group=TRUE)

out1 <- cbind(loop$variable[1],rownames(posthoc$groups),posthoc$groups)

out <- rbind(out,out1)

anova <- anova(model)

out2 <- cbind(loop$variable[1],anova)

out_anova <- rbind(out_anova,out2)

}

names(out) <- c("variable",'forest','value','letter')

result_metrics_test <- out # This is the final data set containing all tests for metrics

names(out_anova) <- c("variable",'df','SS','MS','F','p')

out_anova$p <- round(out_anova$p,4)

# results of anova table with post-hoc tests

#write.csv(out_anova,'Guilds/Manuskript/anova_metrics_All.csv')

#write.csv(out_anova,'Guilds/Manuskript/anova_metrics_Primary.csv')

#write.csv(out_anova,'Guilds/Manuskript/anova_metrics_Secondary.csv')

#write.csv(out_anova,'Guilds/Manuskriptanova_metrics_Predator.csv')

write.csv(out_anova,'Guilds/Manuskript/Region/anova_metrics_All_region.csv')

#write.csv(out_anova,'Guilds/Manuskript/Region/anova_metrics_Primary_region.csv')

#write.csv(out_anova,'Guilds/Manuskript/Region/anova_metrics_Secondary_region.csv')

#write.csv(out_anova,'Guilds/Manuskript/Region/anova_metrics_Predator_region.csv')

####################################################################################################################

# NOW we can plot all the data

# create column to group the metrics

a <- colsplit(result_metrics_melted$variable,'_',c('metric','metricgroup'))

a$metricgroup[a$metricgroup=='IDis'] <- 'Multidimensional'

a$metricgroup[a$metricgroup=='IRic'] <- 'Multidimensional'

a$metricgroup[a$metricgroup=='IEve'] <- 'Multidimensional'

a$metricgroup[a$metricgroup=='IUni'] <- 'Multidimensional'

#levels(result_metrics_melted$metricgroup)[levels(result_metrics_melted$metricgroup)=='IEve'] <- 'Multidimensional'

#evels(result_metrics_melted$metricgroup)[levels(result_metrics_melted$metricgroup)=='IUni'] <- 'Multidimensional'

#levels(result_metrics_melted$metricgroup)[levels(result_metrics_melted$metricgroup)=='IDiv'] <- 'Multidimensional'

result_metrics_melted$metricgroup <- a$metricgroup

result_metrics_melted$metric <- a$metric

#result_metrics_melted$forest <- factor(result_metrics_melted$forest,levels=c('Dou','DouBe','Be','SprBe', 'Spr'))

#Region

result_metrics_melted$forest <- factor(result_metrics_melted$forest,levels=c('Loamy','Sandy'))

levels(result_metrics_melted$metricgroup)[levels(result_metrics_melted$metricgroup)=='IEve'] <- 'Multidimensional'

a <- colsplit(result_metrics_test$variable,'_',c('metric','metricgroup'))

a$metricgroup[a$metricgroup=='IDis'] <- 'Multidimensional'

a$metricgroup[a$metricgroup=='IRic'] <- 'Multidimensional'

a$metricgroup[a$metricgroup=='IEve'] <- 'Multidimensional'

a$metricgroup[a$metricgroup=='IUni'] <- 'Multidimensional'

a$metricgroup[a$metricgroup==''] <- 'Multidimensional'

result_metrics_test$metricgroup <- a$metricgroup

result_metrics_test$metric <- a$metric

#result_metrics_test$forest <- factor(result_metrics_test$forest,levels=c('Dou','DouBe','Be','SprBe', 'Spr'))

## Region

result_metrics_test$forest <- factor(result_metrics_test$forest,levels=c('Sandy','Loamy'))

# Renaming the metrics

table(result_metrics_melted$metric)

result_metrics_melted$metric[result_metrics_melted$metric=='max'] <- 'Maximum'

result_metrics_melted$metric[result_metrics_melted$metric=='min'] <- 'Minimum'

result_metrics_melted$metric[result_metrics_melted$metric=='range'] <- 'Range (max-min)'

result_metrics_melted$metric[result_metrics_melted$metric=='IPos'] <- 'Average position'

result_metrics_melted$metric[result_metrics_melted$metric=='IDiv'] <- 'Isotopic divergence'

result_metrics_melted$metric[result_metrics_melted$metric=='IDis'] <- 'Isotopic dispersion'

result_metrics_melted$metric[result_metrics_melted$metric=='IEve'] <- 'Isotopic eveness'

result_metrics_melted$metric[result_metrics_melted$metric=='IUni'] <- 'Isotopic uniquiness'

result_metrics_melted$metric[result_metrics_melted$metric=='IRic'] <- 'Isotopic richness'

result_metrics_test$metric[result_metrics_test$metric=='max'] <- 'Maximum'

result_metrics_test$metric[result_metrics_test$metric=='min'] <- 'Minimum'

result_metrics_test$metric[result_metrics_test$metric=='range'] <- 'Range (max-min)'

result_metrics_test$metric[result_metrics_test$metric=='IPos'] <- 'Average position'

result_metrics_test$metric[result_metrics_test$metric=='IDiv'] <- 'Isotopic divergence'

result_metrics_test$metric[result_metrics_test$metric=='IDis'] <- 'Isotopic dispersion'

result_metrics_test$metric[result_metrics_test$metric=='IEve'] <- 'Isotopic eveness'

result_metrics_test$metric[result_metrics_test$metric=='IUni'] <- 'Isotopic uniquiness'

result_metrics_test$metric[result_metrics_test$metric=='IRic'] <- 'Isotopic richness'

###### First plot for d13C metrics (use ggpubr package)

###### We need to re scale back and add litter baseline

result_metrics_melted_d13C <- subset(result_metrics_melted,metricgroup=='d13C')

result_metrics_melted_d13C$value[result_metrics_melted_d13C$metric!='Range (max-min)'] <-

(result_metrics_melted_d13C$value[result_metrics_melted_d13C$metric!='Range (max-min)']*(max(mydata$d13C)-

min(mydata$d13C)))+

min(mydata$d13C)

result_metrics_melted_d13C$value[result_metrics_melted_d13C$metric=='Range (max-min)'] <-

(result_metrics_melted_d13C$value[result_metrics_melted_d13C$metric=='Range (max-min)']*(max(mydata$d13C)-

min(mydata$d13C)))

result_metrics_test_d13C <- subset(result_metrics_test,metricgroup=='d13C')

result_metrics_test_d13C$value[result_metrics_test_d13C$metric!='Range (max-min)'] <-

(result_metrics_test_d13C$value[result_metrics_test_d13C$metric!='Range (max-min)']*(max(mydata$d13C)-

min(mydata$d13C)))+

min(mydata$d13C)

result_metrics_test_d13C$value[result_metrics_test_d13C$metric=='Range (max-min)'] <-

(result_metrics_test_d13C$value[result_metrics_test_d13C$metric=='Range (max-min)']*(max(mydata$d13C)-

min(mydata$d13C)))

ggerrorplot(result_metrics_melted_d13C,

x = "forest",

y = "value",

desc_stat = "mean_ci",

color = "forest",

size=1.1) +

geom_jitter(width=.2,alpha=.2)+

ylab(expression(paste(Delta^{13},"C, ","\u2030")))+ # Original syntax: paste("Leaves-calibrated ",delta^{13},"C, ","\u2030")))+

xlab("")+ # I deleted "Leaves-calibrated" and changed "delta" per "Delta"

geom_hline(yintercept = 0)+ # I did the same for N plot.

ggplot2::labs(color = "Forest type",title="C metrics All")+

geom_label(data=result_metrics_test_d13C,aes(x=forest, y=value,label=letter),fill="white",

label.r=unit(0.5, "lines"),color='black',alpha=.6,fontface='bold',size=5.5)+

facet_wrap(~metric,scales='free',nrow=1)+

theme_pubr(base_size = 18) +

theme(legend.position = "none")+

theme(axis.text.x = element_text(angle = 45, hjust = 1, size =18))+

## forest type

#scale_color_manual(values=c("chocolate4","chocolate2","chartreuse2","lightskyblue3", "lightskyblue4"))

## region

scale_color_manual(values=c("#F05039","#1F449C"), breaks=c("Loamy", "Sandy"),labels=c("Loamy", "Sandy")) #+ scale_colour_hue(l=20)

#ggsave("Guilds/Manuskript/Cmetrics_All.jpg",width = 14, height = 7)

#ggsave("Guilds/Manuskript/Cmetrics_Primary.jpg",width = 14, height = 7)

#ggsave("Guilds/Manuskript/Cmetrics_Secondary.jpg",width = 14, height = 7)

#ggsave("Guilds/Manuskript/Cmetrics_Predator.jpg",width = 14, height = 7)

######## for regions

ggsave("Guilds/Manuskript/Region/Cmetrics_All_region.jpg",width = 14, height = 7)

#ggsave("Guilds/Manuskript/Region/Cmetrics_Primary_region.jpg",width = 14, height = 7)

#ggsave("Guilds/Manuskript/Region/Cmetrics_Secondary_region.jpg",width = 14, height = 7)

#ggsave("Guilds/Manuskript/Region/Cmetrics_Predator_region.jpg",width = 14, height = 7)

###### Second plot for d15N metrics

###### We need to re scale back and add litter baseline

result_metrics_melted_d15N <- subset(result_metrics_melted,metricgroup=='d15N')

result_metrics_melted_d15N$value[result_metrics_melted_d15N$metric!='Range (max-min)'] <-

(result_metrics_melted_d15N$value[result_metrics_melted_d15N$metric!='Range (max-min)']*(max(mydata$d15N)-

min(mydata$d15N)))+

min(mydata$d15N)

result_metrics_melted_d15N$value[result_metrics_melted_d15N$metric=='Range (max-min)'] <-

(result_metrics_melted_d15N$value[result_metrics_melted_d15N$metric=='Range (max-min)']*(max(mydata$d15N)-

min(mydata$d15N)))

result_metrics_test_d15N <- subset(result_metrics_test,metricgroup=='d15N')

result_metrics_test_d15N$value[result_metrics_test_d15N$metric!='Range (max-min)'] <-

(result_metrics_test_d15N$value[result_metrics_test_d15N$metric!='Range (max-min)']*(max(mydata$d15N)-

min(mydata$d15N)))+

min(mydata$d15N)

result_metrics_test_d15N$value[result_metrics_test_d15N$metric=='Range (max-min)'] <-

(result_metrics_test_d15N$value[result_metrics_test_d15N$metric=='Range (max-min)']*(max(mydata$d15N)-

min(mydata$d15N)))

ggerrorplot(result_metrics_melted_d15N,

x = "forest",

y = "value",

desc_stat = "mean_ci",

color = "forest",

size=1.1) +

geom_jitter(width=.2,alpha=.2)+

ylab(expression(paste(Delta^{15},"N, ","\u2030")))+

xlab("")+

ggplot2::labs(color = "Forest type",title="N metrics All")+

geom_label(data=result_metrics_test_d15N,aes(x=forest, y=value,label=letter),fill="white",

label.r=unit(0.5, "lines"),color='black',alpha=.6,fontface='bold',size=5.5)+

facet_wrap(~metric,scales='free',nrow=1)+

theme_pubr(base_size = 18) +

theme(legend.position = "bottom")+

theme(axis.text.x = element_text(angle = 45, hjust = 1, size =18))+

### forest type

#scale_color_manual(values=c("chocolate4","chocolate2","chartreuse2","lightskyblue3", "lightskyblue4"))

#region

scale_color_manual(values=c("#F05039","#1F449C","#F8766d","#00bfc4","#F8766d"), breaks=c("Loamy", "Sandy"),labels=c("Loamy", "Sandy")) #+ scale_colour_hue(l=20)

#ggsave("Guilds/Manuskript/Nmetrics_All.jpg",width = 14, height = 7)

#ggsave("Guilds/Manuskript/Nmetrics_Primary.jpg",width = 14, height = 7)

#ggsave("Guilds/Manuskript/Nmetrics_Secondary.jpg",width = 14, height = 7)

#ggsave("Guilds/Manuskript/Nmetrics_Predator.jpg",width = 14, height = 7)

####for region

ggsave("Guilds/Manuskript/Region/Nmetrics_All_region.jpg",width = 14, height = 7)

#ggsave("Guilds/Manuskript/Region/Nmetrics_Primary_region.jpg",width = 14, height = 7)

#ggsave("Guilds/Manuskript/Region/Nmetrics_Secondary_region.jpg",width = 14, height = 7)

#ggsave("Guilds/Manuskript/Region/Nmetrics_Predator_region.jpg",width = 14, height = 7)

###### Third plot for Multidimensional metrics

ggerrorplot(subset(result_metrics_melted,metricgroup=='Multidimensional'),

x = "forest",

y = "value",

desc_stat = "mean_ci",

color = "forest",

size=1.1) +

geom_jitter(width=.2,alpha=.2)+

ylab("Metric value")+

xlab("")+

ggplot2::labs(color = "Forest type",title="Multidimensional metrics All")+

geom_label(data=subset(result_metrics_test,metricgroup=='Multidimensional'),aes(x=forest, y=value,label=letter),fill="white",

label.r=unit(0.5, "lines"),color='black',alpha=.6,fontface='bold',size=5.5)+

facet_wrap(~metric,scales='free',nrow=1)+

theme_pubr(base_size = 18) +

theme(legend.position = "bottom")+

theme(axis.text.x = element_text(angle = 45, hjust = 1, size =18))+

## forest type

#scale_color_manual(values=c("chocolate4","chocolate2","chartreuse2","lightskyblue3", "lightskyblue4"))

#region

scale_color_manual(values=c("#F05039","#1F449C","#f8766d","#00afc4","#F8766d"), breaks=c("Loamy", "Sandy"),labels=c("Loamy", "Sandy")) #+ scale_colour_hue(l=20)

#ggsave("Guilds/Manuskript/multidimensional_All.jpg",width = 14, height = 7)

#ggsave("Guilds/Manuskript/multidimensional_Primary.jpg",width = 14, height = 7)

#ggsave("Guilds/Manuskript/multidimensional_Secondary.jpg",width = 14, height = 7)

#ggsave("Guilds/Manuskript/multidimensional_Predator.jpg",width = 14, height = 7)

## regions

ggsave("Guilds/Manuskript/Region/multidimensional_All_region.jpg",width = 14, height = 7)

#ggsave("Guilds/Manuskript/Region/multidimensional_Primary_region.jpg",width = 14, height = 7)

#ggsave("Guilds/Manuskript/Region/multidimensional_Secondary_region.jpg",width = 14, height = 7)

#ggsave("Guilds/Manuskript/Region/multidimensional_Predator_region.jpg",width = 14, height = 7)

###### Fourth plot, Bi-plot of the whole community

result_means <- as.data.frame(meanSI_group(mydata),weight=mydata$weight)

result_means$plot <- rownames(result_means)

#result_means <- result_means %>% separate(plot, sep = "-", c('region', 'id_forest'), remove = FALSE)

result_means <- result_means %>% separate(plot, sep = "_", c('region', 'id_forest'), remove = FALSE)

result_means$forest_id <- substr(result_means$plot,3,3)

result_means <- result_means %>% mutate(result_means, forest = case_when (endsWith(id_forest, "1") ~ "Dou",

endsWith(id_forest, "2") ~ "DouBe",

endsWith(id_forest, "3") ~ "Be",

endsWith(id_forest, "4") ~ "SprBe",

endsWith(id_forest, "5") ~ "Spr"))

result_means$forest <- factor(result_means$forest,levels=c('Dou','DouBe','Be','SprBe', 'Spr'))

result_means$se_d13C <- result_means$sd_d13C/(result_means$n)^0.5

result_means$se_d15N <- result_means$sd_d15N/(result_means$n)^0.5

hulls <- ddply(result_means,"forest",find_hull) # Just for Convex hulls

biplot <- ggplot(result_means,aes(x=d13C,y=d15N,fill=forest))+

geom_errorbar(aes(ymin=d15N-se_d15N,ymax=d15N+se_d15N),color='grey')+

geom_errorbarh(aes(xmin=d13C-se_d13C,xmax=d13C+se_d13C),color='grey')+

geom_point(shape=21,size=4)+

geom_polygon(data = hulls, aes(fill=forest), alpha = 0.5)+

xlab(expression(paste(Delta^{13},"C, ","\u2030")))+

ylab(expression(paste(Delta^{15},"N, ","\u2030")))+

ggplot2::labs(color = "Forest type",fill = "Forest Type")+

theme_pubr(base_size = 20)+

scale_fill_manual(values=c("chocolate4","chocolate2","chartreuse3","lightskyblue3", "lightskyblue4"))+

scale_color_manual(values=c("chocolate4","chocolate2","chartreuse3","lightskyblue3", "lightskyblue4"))

biplot

#ggsave("Guilds/Manuskript/biplot_All.jpg",width = 7, height = 7)

#ggsave("Guilds/Manuskript/biplot_Primary.jpg",width = 7, height = 7)

#ggsave("Guilds/Manuskript/biplot_Secondary.jpg",width = 7, height = 7)

#ggsave("Guilds/Manuskript/biplot_Predator.jpg",width = 7, height = 7)

ggsave("Guilds/Manuskript/Region/biplot_All.jpg",width = 7, height = 7)

#ggsave("Guilds/Manuskript/Region/biplot_Primary.jpg",width = 7, height = 7)

#ggsave("Guilds/Manuskript/Region/biplot_Secondary.jpg",width = 7, height = 7)

#ggsave("Guilds/Manuskript/Region/biplot_Predator.jpg",width = 7, height = 7)

####################################################################################################################

##################################################################################################################################################################

##################################################################################################################################################################

# 'si_div': a script with 4 R functions: 'meanSI_group', 'scaleSI_range01', 'IDiversity', 'IOverlap'

# for computing isotopic diversity indices based on stable isotope values.

#

# The help of each function is provided above its script.

#

# Examples of how to use these functions are provided in "examples" R script file

#

# after the article by Julien Cucherousset & Sébastien Villéger

#

# Author: Sébastien Villéger: sebastien.villeger@univ-montp2.fr

#

# IMPORTANT: The R libraries 'geometry', 'ape' and 'rcdd' need to be installed

#

##################################################################################################################################################################

##################################################################################################################################################################

# loading libraries

require(geometry)

require(ape)

require(rcdd)

# codes for stable isotope ratios

nm_si<-c("d13C","d15N","dD","d34S")

##################################################################################################################################################################

##################################################################################################################################################################

#

# 'meanSI_group': function to compute mean Stable Isotope values for different elements at the group level using individual stable isotope values

#

# INPUT: -'dataset' a dataframe or matrix with at least 3 columns:

# $ 'group': a numeric or character variable with the identity (e.g. life stage, sex, population, species) of each individual

# $ 'd13C', 'd15N', 'dD' and/or 'd34S': at least two numeric variables with stable isotope value f each individual

# $ 'weight': an optional numeric variable with weight (e.g. body mass, dry mass) of each individual

#

# OUTPUT: a matrix with for each group of individuals (rows): number of individuals ('n'), mean stable isotope value (e.g. 'd13C', 'd15N'),

# standard deviation of stable isotope values (e.g. 'd13C_sd', 'd15N_sd'), and total weight of each group ('weight') [NA if no weight as input].

#

# NB: This matrix can be used directly as an INPUT for the subsequent functions: 'scaleSI_rge01', 'IDiversity' and 'IOverlap'

##################################################################################################################################################################

meanSI_group<-function(dataset) {

# names of groups and abbreviation (4 characters)

gr<-levels(as.factor(as.character(dataset[,"group"])))

# number of groups

nbgr<-length(gr)

# computing number of individuals per group

nbind_gr<-summary(as.factor(as.character(dataset[,"group"])))

# number and names of elements used

nmel<-colnames(dataset)[which(colnames(dataset) %in% nm_si)]

nbel<-length(nmel)

# isotopic signature of individuals

si<-as.matrix(dataset[,nmel])

# computing mean isotopic signature and total abundance of each group (if abundances provided)

data_gr<-matrix(NA,nbgr,2+nbel*2, dimnames=list(gr, c("n", nmel, paste("sd",nmel,sep="_"), "weight" ) ) )

for (k in gr)

{

rowk<-which(dataset[,"group"]==k)

data_gr[k,"n"]<-length(rowk)

if( "weight" %in% colnames(dataset)==T) { data_gr[k,"weight"]<-sum(dataset[rowk,"weight"]) } # end of weight

for (e in nmel)

{data_gr[k,e]<-mean(si[rowk,e])

data_gr[k,paste("sd",e,sep="_")]<-sd(si[rowk,e]) } # end of e

} # end of k

return(data_gr)

} # end of function meanSI_group

##################################################################################################################################################################

##################################################################################################################################################################

#

# scaleSI_range01: function to standardize Stable Isotope values of a dataset given a reference dataset of organisms (individuals or group of individuals)

#

# INPUTS:- 'raw_data': a dataframe or matrix with stable isotope values for at least 2 elements (columns) for several groups (rows).

# Columns' names should be a subset of ('d13C','d15N','dD','d34S').

# - 'all_data': a dataframe or matrix with stable isotope values for the same elements than 'raw_data' (columns) with an equal or larger set of samples (rows).

# By default, 'all_data' is identical to 'raw_data'.

#

# OUTPUT: a matrix similar to 'raw_data' with scaled stable isotope values (each ranging from 0 to 1).

# If standard deviation values are provided, they are scaled based on the range of mean values.

#

##################################################################################################################################################################

scaleSI_range01<-function(raw_data, all_data=raw_data) {

# names of elements used to describe data

nmel_raw_data<-colnames(raw_data)[which(colnames(raw_data) %in% nm_si)]

si_raw_data<-as.matrix(raw_data[,nmel_raw_data])

nmel<-nmel_raw_data ; nbel<-length(nmel_raw_data)

# checking that elements used to describe raw data are present in whole dataset

if ( sum(nmel_raw_data %in% colnames(raw_data)) !=nbel ) stop("error: the elements present in 'raw_data' must be present in 'all_data'")

# matrix to store results

data_scaled<-raw_data

# scaling data according to range of whole dataset

for (e in nmel)

data_scaled[,e]<-(raw_data[,e]-min(all_data[,e],na.rm=T))/(max(all_data[,e],na.rm=T)-min(all_data[,e],na.rm=T))

# scaling standard deviation (if any)

nmelsd<-paste("sd",nmel,sep="_")

if(sum(nmelsd %in% colnames(raw_data) )==nbel) {

for (e in nmel)

data_scaled[,paste("sd",e,sep="_")]<-raw_data[,paste("sd",e,sep="_")]/(max(all_data[,e],na.rm=T)-min(all_data[,e],na.rm=T))

} # end of if sd

return(data_scaled)

} # end of scale_range01

##################################################################################################################################################################

##################################################################################################################################################################

# generic graphical functions used in 'IDiversity' and 'IOverlap'

# axis titles for the 4 types of istopes used

tit_d13C<-expression(bold(paste(delta^"13",C,sep=""))) ; scl_tit_d13C<-expression(bold(paste("Scaled ",delta^"13",C,sep="")))

tit_d15N<-expression(bold(paste(delta^"15",N,sep=""))) ; scl_tit_d15N<-expression(bold(paste("Scaled ",delta^"15",N,sep="")))

tit_dD<-expression(bold(paste(delta^"",D,sep=""))) ; scl_tit_dD<-expression(bold(paste("Scaled ",delta^"",D,sep="")))

tit_d34S<-expression(bold(paste(delta^"34",S,sep=""))) ; scl_tit_d34S<-expression(bold(paste("Scaled ",delta^"34",S,sep="")))

# graphic function to plot isotopic space

isotopic_space<-function(nmX="X",nmY="Y", limX=c(-0.05,1.05),limY=c(-0.05,1.05), labX=c(0,0.25,0.5,0.75,1),labY=c(0,0.25,0.5,0.75,1) ) {

# setting graphical parameters

par(mar=c(4,4.5,4,3.5)) ; tick=-0.4 ; lasX=1 ;lasY=1 ; lineX=-0.2 ; lineY=-0.2 ; cexX=0.9 ; cexY=0.9 ; lineXt=lineX+2.1 ; lineYt=lineY+2.2 ; cexXt=1 ; cexYt=1

# empty window

plot(limX,limY,type="n",axes=F,xaxt="n",yaxt="n",xlab="",ylab="",xlim=limX,ylim=limY)

rect(limX[1],limY[1],limX[2],limY[2]) # border

# X axis

axis(side=1, at=labX, labels=F, tcl=tick, pos=limY[1]) # X thicks

mtext(side=1, labX, at=labX, line=lineX, cex=cexX, las=lasX) # X labels

mtext(side=1,nmX,cex=cexXt,line=lineXt,font=2) # X title

# Y axis

axis(side=2, at=labY, labels=F, tcl=tick, pos=limX[1]) # Y thicks

mtext(side=2, labY, at=labY, line=lineY, cex=cexY, las=lasY) # Y labels

mtext(side=2,nmY,cex=cexYt,line=lineYt,font=2) # Y title

} # end of isotopic_space

# function to add vertical and horizontal error bars

meansexy<-function(meanxy,sexy,colb="black",lg=0.1) {

segments(meanxy[,1]-sexy[,1],meanxy[,2],meanxy[,1]+sexy[,1],meanxy[,2],col=colb) # x error bar

segments(meanxy[,1],meanxy[,2]-sexy[,2],meanxy[,1],meanxy[,2]+sexy[,2],col=colb) # y error bar

segments(meanxy[,1]-sexy[,1],meanxy[,2]-lg,meanxy[,1]-sexy[,1],meanxy[,2]+lg,col=colb)

segments(meanxy[,1]+sexy[,1],meanxy[,2]-lg,meanxy[,1]+sexy[,1],meanxy[,2]+lg,col=colb)

segments(meanxy[,1]-lg,meanxy[,2]-sexy[,2],meanxy[,1]+lg,meanxy[,2]-sexy[,2],col=colb)

segments(meanxy[,1]-lg,meanxy[,2]+sexy[,2],meanxy[,1]+lg,meanxy[,2]+sexy[,2],col=colb)

} # end of meansexy

##################################################################################################################################################################

##################################################################################################################################################################

#

# 'IDiversity': function to compute complementary indices describing the isotopic diversity for a group of organisms (individuals in a population or species in a community)

#

# INPUTS:

# - 'cons': a dataframe or matrix with stable isotope values for at least 2 elements (columns) for several organisms (rows).

# Columns' names should be a subset of ('d13C','d15N','dD','d34S').

# It may include standard deviation of mean values (coded as 'sd_d13C', 'sd_d15N',...).

# These standard deviation values are used only for illustrative purposes in the graphical outputs.

#

# - 'weight': a numeric vector with weight of organisms (e.g. individual mass, or relative abundance of species in the community). By default all weight equals 1.

# - 'nm_plot': a single character string specifying the name of the .jpeg file where graphics illustrating isotopic diversity will be stored. Default is NA, i.e. no graphics.

# - 'col': a single color, coded as hexadecimal characters, for points and convex hull filling. Default is green.

# - 'transp': a single numeric value indicating the percentage of transparency for convex hulls filling. Default is 50%.

# - 'scaled': a logical value indicating wether isotopic values have been scaled to have a range between 0 and 1.

# If TRUE (default) axes of graphics fill at least the 0-1 range and axes titles specify the scaling procedure

#

# OUTPUTS: - a vector with minimum, maximum, range and abundance-weighted mean values for each isotopic axis (e.g. 'min_d13C', 'max_d13C', 'range_d13C', 'IPos_d13C' for d13C)

# and 5 multidimensional indices: isotopic richness ('IRic'), isotopic evenness ('IEve') and isotopic divergence ('IDiv'), isotopic dispersion ('IDis') and isotopic uniqueness ('IUni').

#

# - for each pair of elements a 6-panels .jpeg file (e.g. ‘nm_plot’_d13C_d15N.jpeg).

# All axes have the same range to illustrate potential bias if no standardization has been done prior indices computation.

# For each panel, points representing organisms position in the isotopic niche space (and associated standard deviation if values have been provided in “cons” dataframe).

# Weights of organisms are illustrated proportionally to point surface and a legend is displayed in the bottom right corner.

#

# * top left panel: Isotopic position, i.e. weighted-mean values of the organisms on each axis, is illustrated with a square and horizontal and vertical dashed lines.

# * top middle panel: Isotopic richness is shown as the colored area. Filled points are organisms being vertices in the multidimensional space.

# If more than two elements were used to build the isotopic space, the convex polygon is a projection of the multidimensional convex hull in 2D.

# Minimum and maximum values on each axis are illustrated by vertical bars.

# * top right panel: Isotopic divergence is illustrated through the center of gravity of the vertices (diamond) and al the distances to it (dashed lines).

# * bottom left panel: Isotopic dispersion is symbolized by the center of gravity of all points (white square) and all the distances to it (dotted lines).

# * bottom middle panel: Isotopic evenness is illustrated with the minimum spanning tree linking all points in the multidimensional space.

# * bottom right panel: Isotopic uniqueness is symbolized with all the distances to nearest organism (black arrows).

#

##################################################################################################################################################################

IDiversity<-function(cons, weight=rep(1,nrow(cons)), nm_plot=NA, col="#477D00", transp=50, scaled=TRUE) {

# names of elements used to describe consumers

nmel<-colnames(cons)[which(colnames(cons) %in% nm_si)]

nbel<-length(nmel)

# stable isotope signature

si<-as.matrix(cons[,nmel])

# checking weighting for all individuals

if(length(weight) != nrow(cons)) stop(paste(" error: weight does not have the same length than number of consumers"))

# relative weight

rel_weight<-weight/sum(weight)

# checking number of consumers is higher than number of elements

if (nrow(cons)<(nbel+1)) stop(paste(" error: computing indices using",nbel,"elements requires at least",nbel+1," consumers"))

# vector to store results

ID<-rep(NA,nbel*4+5) ; names(ID)<-c(paste("min",nmel,sep="_"), paste("max",nmel,sep="_"), paste("range",nmel,sep="_"), paste("IPos",nmel,sep="_"), c("IRic","IDiv","IDis","IEve","IUni") )

###########################################################

# computing indices values on each axis

# range of traits values

ID[paste("min",nmel,sep="_")]<-apply(si,2,min)

ID[paste("max",nmel,sep="_")]<-apply(si,2,max)

ID[paste("range",nmel,sep="_")]<-ID[paste("max",nmel,sep="_")]-ID[paste("min",nmel,sep="_")]

# abundance-weighted mean values

ID[paste("IPos",nmel,sep="_")]<-rel_weight%*%si

###############################################################################################################################

# generic functions for computing multidimensional diversity indices

I_RED<-function(coord,relab ) {

# number of species

S<-nrow(coord)

###########################################################

# Richness

IRic<-round(convhulln(coord,"FA")$vol,6)

# identity of vertices

vert0<-convhulln(coord,"Fx TO 'vert.txt'")

vert1<-scan("vert.txt",quiet=T)

vertices<-(vert1+1)[-1]

###########################################################

# Evenness

# inter-species Euclidean distance

distT<-dist(coord, method="euclidian")

# topology of Minimum Spanning Tree and conversion of the 'mst' matrix into 'dist' class

linkmst<-mst(distT)

mstvect<-as.dist(linkmst)

# pairwise cumulative relative abundances and conversion into 'dist' class

ab2<-matrix(0,nrow=S,ncol=S)

for (q in 1:S)

for (r in 1:S)

ab2[q,r]<-relab[q]+relab[r] # end of q,r

ab2vect<-as.dist(ab2)

# EW index for the (S-1) segments

EW<-rep(0,S-1)

flag<-1

for (m in 1:((S-1)*S/2))

{if (mstvect[m]!=0) {EW[flag]<-distT[m]/(ab2vect[m]) ; flag<-flag+1}} # end of m

# PEW index and comparison with 1/S-1

minPEW<-rep(0,S-1) ; OdSmO<-1/(S-1)

for (l in 1:(S-1))

minPEW[l]<-min( (EW[l]/sum(EW)) , OdSmO ) # end of l

# IEve

IEve<-round( ( (sum(minPEW))- OdSmO) / (1-OdSmO ) ,6)

###############################################################

# Divergence

# coordinates of vertices

coordvertices<-coord[vertices,]

# coordinates of the center of gravity of the vertices (B)

B<-apply(coordvertices,2,mean)

# Euclidean dstance to B (dB)

dB<-apply(coord, 1, function(x) { (sum((x-B)^2) )^0.5} )

# mean of dB values and deviations to mean

meandB<-mean(dB)

devdB<-dB-meandB

# abundance-weighted mean deviation

abdev<-relab*devdB

ababsdev<-relab*abs(devdB)

# IDiv

IDiv<-round( (sum(abdev)+meandB) / (sum(ababsdev)+meandB) ,6)

####################################################################

# results

indices<-c(IRic,IEve,IDiv) ; names(indices)<-c("IRic","IEve","IDiv")

detailsRED<-list(vertices=vertices, mst=linkmst, B=B, meandB=meandB)

I_RED<-list(indices=indices, details=detailsRED )

invisible(I_RED)

} # end of function I_RED

########################################################################################################################################

# multivariate indices from Villeger et al 2008

ired<-I_RED(si,rel_weight)

ID[c("IRic","IEve","IDiv")]<-ired$indices

# Isotopic dispersion: scaled abundance-weighted mean distance to abundance-weighted centroid

dist_centr<-apply(si, 1, function(x) { (sum((x-ID[paste("IPos",nmel,sep="_")])^2) )^0.5} ) # distance to abundance-weighted centroid

ID["IDis"]<-(rel_weight %*% dist_centr)/ max(dist_centr) # scaling between 0(=all biomass on the centroid) and 1(=all biomass on the most extreme point)

# Isotopic originality : scaled abundance weighted mean distance to nearest neighbour

# for each organism distance to, and identity of, nearest neighbour

dist_T<-as.matrix(dist(si,method="euclidean")) ; dist_T[which(dist_T==0)]<-NA

oriT<-apply(dist_T, 1, min, na.rm=T )

NN<-dist_T ; NN<-NN-apply(NN,1,min,na.rm=T) ; NN[which(NN!=0)]<-NA ; NN[which(NN==0)]<-1

ID["IUni"]<-(oriT %*% rel_weight) / max(oriT) # abundance weighting and scaling by maximal distance between 2 points

########################################################################################################################################

########################################################################################################################################

# graphical output

if( is.na(nm_plot)==FALSE) {

# setting axes limits given consumers signature for all elements

nmelsd<-paste("sd",nmel,sep="_")

min_axes<- apply(cons[,nmel], 2, min, na.rm=T) ; max_axes<- apply(cons[,nmel], 2, max, na.rm=T) # limits of each axis

# limits of each axis given sd

if(sum(nmelsd %in% colnames(cons) )==nbel)

{min_axes<-apply(cons[,nmel]-cons[,nmelsd], 2, min, na.rm=T)

max_axes<-apply(cons[,nmel]+cons[,nmelsd], 2, max, na.rm=T) }

# same range on each axis for graphics: min=observed minimal value - 5% maximal range ; max=observed minimal value + maximal range + 5% maximal range

rge_axes<-max_axes-min_axes # range on each axis

newlim_axes<-matrix(0,length(nmel),2, dimnames=list(nmel,c("min","max") ) )

newlim_axes[,"min"]<-min_axes-max(rge_axes)*0.05

newlim_axes[,"max"]<-min_axes+max(rge_axes)*1.05

rge_plot<-max(rge_axes)*1.1

# one jpeg file per pair of elements with 6 panels

for (e1 in 1:(nbel-1))

for (e2 in (e1+1):nbel)

{

# names of elements

nmel1<-nmel[e1] ; eval(parse(text=paste("tit1<-tit_",nmel1,sep="") ) )

nmel2<-nmel[e2] ; eval(parse(text=paste("tit2<-tit_",nmel2,sep="") ) )

nmel12<-c(nmel1,nmel2)

# creating jpeg file

nmjpeg<-paste(nm_plot,"_",nmel1,"_",nmel2,".jpeg",sep="")

jpeg(file=nmjpeg, res=150, width=1200, height=1800)

layout(matrix(c(1:6),3,2,T)) ; layout.show(6)

# limits of axes

lim_1<-newlim_axes[nmel1,]

lim_2<-newlim_axes[nmel2,]

# if axes are for scaled isotope values, "Scaled" in axis title and range is at least from 0 to 1

if (scaled==TRUE) {

tit1<-eval(parse(text=paste("tit1<-scl_tit_",nmel1,sep="") ) ); tit2<-eval(parse(text=paste("tit2<-scl_tit_",nmel2,sep="") ) )

lim_1<-c( min(-0.05, lim_1[1]) , max(1.05,lim_1[2]) )

lim_2<-c( min(-0.05, lim_2[1]) , max(1.05,lim_2[2]) )

} # end of if scaled axes

# labels on axes

lab_1<-pretty(lim_1,n=5,min.n=4) ; lab_1<-lab_1[which(lab_1>=lim_1[1] & lab_1<=lim_1[2])]

lab_2<-pretty(lim_2,n=5,min.n=4) ; lab_2<-lab_2[which(lab_2>=lim_2[1] & lab_2<=lim_2[2])]

###############################################################

# Isotopic Position

# Isotopic space given axes limits set using consumers signature

isotopic_space(nmX=tit1,nmY=tit2, limX=lim_1, limY=lim_2, labX=lab_1,labY=lab_2 )

# mean value

segments(ID[paste("IPos_",nmel1,sep="")],ID[paste("IPos_",nmel2,sep="")], ID[paste("IPos_",nmel1,sep="")], min(lim_2), lwd=1.5, col=col, lty=2)

segments(ID[paste("IPos_",nmel1,sep="")],ID[paste("IPos_",nmel2,sep="")], min(lim_1) ,ID[paste("IPos_",nmel2,sep="")], lwd=1.5, col=col, lty=2)

points( ID[paste("IPos_",nmel1,sep="")],ID[paste("IPos_",nmel2,sep="")], pch=22, bg="white", col=col,cex=2.5)

# abundances, scaling: point area proportional to relative abundance, if relab=100%, circle diameter=15% of axis range

sizeab<-sqrt(rel_weight)*0.075*rge_plot

symbols(si[,nmel1],si[,nmel2], circles=sizeab, inches=FALSE, bg=col, fg=col, add=TRUE)

# legend for abundance

rect(max(lim_1)-0.25*rge_plot, min(lim_2), max(lim_1), min(lim_2)+0.12*rge_plot)

symbols(max(lim_1)-0.19*rge_plot, min(lim_2)+0.06*rge_plot, circles=sqrt(0.1)*0.075*rge_plot, inches=FALSE, bg="black", fg="black", add=TRUE, lwd=1.5)

text(max(lim_1)-0.15*rge_plot, min(lim_2)+0.06*rge_plot,"10%", adj=c(0,0.5) )

# error bars if any

if(sum(nmelsd %in% colnames(cons) )==nbel) { meansexy(meanxy=si[,nmel12], sexy=cons[,paste("sd",nmel12,sep="_")],colb=col,lg=0.01*rge_plot ) }# sd

# index

mtext(side=3, tit1, at=min(lim_1)+rge_plot*0.1, line=-0.4, cex=0.7,adj=1)

mtext(side=3, paste(": IPos=",round(ID[paste("IPos_",nmel1,sep="")],3),sep=""), at=min(lim_1)+rge_plot*0.1, line=-0.4, cex=0.7,adj=0)

mtext(side=3, tit2, at=mean(lim_1)+rge_plot*0.1, line=-0.4, cex=0.7,adj=1)

mtext(side=3, paste(": IPos=",round(ID[paste("IPos_",nmel2,sep="")],3),sep=""), at=mean(lim_1)+rge_plot*0.1, line=-0.4, cex=0.7,adj=0)

mtext(side=3, "Isotopic Position", at=mean(lim_1), line=1.1, cex=0.8,adj=0.5, font=2)

###############################################################

# Isotopic Richness

# Isotopic space given axes limits set using consumers signature

isotopic_space(nmX=tit1,nmY=tit2, limX=lim_1, limY=lim_2, labX=lab_1,labY=lab_2 )

# range on each axis

dec1<-rge_plot*0.02

segments( ID[paste("min_",nmel1,sep="")], min(lim_2)-dec1, ID[paste("min_",nmel1,sep="")], min(lim_2)+dec1, col=col , lwd=3) # min x

segments( ID[paste("max_",nmel1,sep="")], min(lim_2)-dec1, ID[paste("max_",nmel1,sep="")], min(lim_2)+dec1, col=col , lwd=3) # max x

segments( min(lim_1)-dec1, ID[paste("min_",nmel2,sep="")], min(lim_1)+dec1, ID[paste("min_",nmel2,sep="")], col=col , lwd=3) # min y

segments( min(lim_1)-dec1, ID[paste("max_",nmel2,sep="")], min(lim_1)+dec1, ID[paste("max_",nmel2,sep="")], col=col , lwd=3) # max y

# projected convex hull in 2D

vert0<-convhulln(si[,nmel12],"Fx TO 'vert.txt'")

vert1<-scan("vert.txt",quiet=T) ; vertices2D<-(vert1+1)[-1]

polygon(si[vertices2D,nmel12],border=NA,col=paste(col,transp,sep=""))

# all points (empty) then filling points being vertices in nD

points(si[,nmel12], pch=21,bg=NA, col=col[1],cex=2)

points(si[ired$details$vertices,nmel12], pch=21,bg=col, col=col,cex=2)

# error bars if any

if(sum(nmelsd %in% colnames(cons) )==nbel) { meansexy(meanxy=si[,nmel12], sexy=cons[,paste("sd",nmel12,sep="_")],colb=col,lg=0.01*rge_plot ) }# sd

# index

mtext(side=3, tit1, at=min(lim_1)+rge_plot*0.1, line=-0.4, cex=0.7,adj=1)

mtext(side=3, paste(": ",round(ID[paste("range_",nmel1,sep="")],1), " [",round(ID[paste("min_",nmel1,sep="")],1),";",round(ID[paste("max_",nmel1,sep="")],1),"]",sep=""), at=min(lim_1)+rge_plot*0.1, line=-0.4, cex=0.7,adj=0)

mtext(side=3, tit2, at=mean(lim_1)+rge_plot*0.1, line=-0.4, cex=0.7,adj=1)

mtext(side=3, paste(": ",round(ID[paste("range_",nmel2,sep="")],1), " [",round(ID[paste("min_",nmel2,sep="")],1),";",round(ID[paste("max_",nmel2,sep="")],1),"]",sep=""), at=mean(lim_1)+rge_plot*0.1, line=-0.4, cex=0.7,adj=0)

mtext(side=3, paste("Isotopic Richness=",round(ID['IRic'],3),sep=""), at=mean(lim_1), line=1.1, cex=0.8,adj=0.5, font=2)

###############################################################

# Isotopic Divergence

# Isotopic space given axes limits set using consumers signature

isotopic_space(nmX=tit1,nmY=tit2, limX=lim_1, limY=lim_2, labX=lab_1,labY=lab_2 )

# projected convex hull in 2D

vert0<-convhulln(si[,nmel12],"Fx TO 'vert.txt'")

vert1<-scan("vert.txt",quiet=T) ; vertices2D<-(vert1+1)[-1]

polygon(si[vertices2D,nmel12],border=col,col=NA, lwd=1 )

segments(ired$details$B[nmel1], ired$details$B[nmel2], si[,nmel1],si[,nmel2],col=col, lty=2, lwd=2)

points(ired$details$B[nmel1], ired$details$B[nmel2], pch=23,col=col,bg="white",cex=2.5)

# abundances, scaling: point area proportional to relative abundance, if relab=100%, circle diameter=15% of axis range

sizeab<-sqrt(rel_weight)*0.075*rge_plot

symbols(si[,nmel1],si[,nmel2], circles=sizeab, inches=FALSE, bg=col, fg=col, add=TRUE)

# legend for abundance

rect(max(lim_1)-0.25*rge_plot, min(lim_2), max(lim_1), min(lim_2)+0.12*rge_plot)

symbols(max(lim_1)-0.19*rge_plot, min(lim_2)+0.06*rge_plot, circles=sqrt(0.1)*0.075*rge_plot, inches=FALSE, bg="black", fg="black", add=TRUE, lwd=1.5)

text(max(lim_1)-0.15*rge_plot, min(lim_2)+0.06*rge_plot,"10%", adj=c(0,0.5) )

# error bars if any

if(sum(nmelsd %in% colnames(cons) )==nbel) { meansexy(meanxy=si[,nmel12], sexy=cons[,paste("sd",nmel12,sep="_")],colb=col,lg=0.01*rge_plot ) }# sd

# index

mtext(side=3, paste("Isotopic Divergence=",round(ID['IDiv'],3),sep=""), at=mean(lim_1), line=0.5, cex=0.8,adj=0.5, font=2)

###############################################################

# Isotopic Dispersion

# Isotopic space given axes limits set using consumers signature

isotopic_space(nmX=tit1,nmY=tit2, limX=lim_1, limY=lim_2, labX=lab_1,labY=lab_2 )

# distance to abundance weighted centroid

segments(ID[paste("IPos_",nmel1,sep="")],ID[paste("IPos_",nmel2,sep="")], si[,nmel1],si[,nmel2],col=col, lty=3, lwd=2)

points( ID[paste("IPos_",nmel1,sep="")],ID[paste("IPos_",nmel2,sep="")], pch=22, bg="white", col=col,cex=2.5)

# abundances, scaling: point area proportional to relative abundance, if relab=100%, circle diameter=15% of axis range

sizeab<-sqrt(rel_weight)*0.075*rge_plot

symbols(si[,nmel1],si[,nmel2], circles=sizeab, inches=FALSE, bg=col, fg=col, add=TRUE)

# legend for abundance

rect(max(lim_1)-0.25*rge_plot, min(lim_2), max(lim_1), min(lim_2)+0.12*rge_plot)

symbols(max(lim_1)-0.19*rge_plot, min(lim_2)+0.06*rge_plot, circles=sqrt(0.1)*0.075*rge_plot, inches=FALSE, bg="black", fg="black", add=TRUE, lwd=1.5)

text(max(lim_1)-0.15*rge_plot, min(lim_2)+0.06*rge_plot,"10%", adj=c(0,0.5) )

# error bars if any

if(sum(nmelsd %in% colnames(cons) )==nbel) { meansexy(meanxy=si[,nmel12], sexy=cons[,paste("sd",nmel12,sep="_")],colb=col,lg=0.01*rge_plot ) }# sd

# index

mtext(side=3, paste("Isotopic Dispersion=",round(ID['IDis'],3),sep=""), at=mean(lim_1), line=0.5, cex=0.8,adj=0.5, font=2)

###############################################################

# Isotopic Evenness

# Isotopic space given axes limits set using consumers signature

isotopic_space(nmX=tit1,nmY=tit2, limX=lim_1, limY=lim_2, labX=lab_1,labY=lab_2 )

# MST

for (j in 1:nrow(ired$details$mst))

for (i in 1:nrow(ired$details$mst))

if (ired$details$mst[j,i]==1 & j>i) segments(si[,nmel1][j], si[,nmel2][j], si[,nmel1][i], si[,nmel2][i], col=col, lwd=1.5)

# abundances, scaling: point area proportional to relative abundance, if relab=100%, circle diameter=15% of axis range

sizeab<-sqrt(rel_weight)*0.075*rge_plot

symbols(si[,nmel1],si[,nmel2], circles=sizeab, inches=FALSE, bg=col, fg=col, add=TRUE)

# legend for abundance

rect(max(lim_1)-0.25*rge_plot, min(lim_2), max(lim_1), min(lim_2)+0.12*rge_plot)

symbols(max(lim_1)-0.19*rge_plot, min(lim_2)+0.06*rge_plot, circles=sqrt(0.1)*0.075*rge_plot, inches=FALSE, bg="black", fg="black", add=TRUE, lwd=1.5)

text(max(lim_1)-0.15*rge_plot, min(lim_2)+0.06*rge_plot,"10%", adj=c(0,0.5) )

# error bars if any

if(sum(nmelsd %in% colnames(cons) )==nbel) { meansexy(meanxy=si[,nmel12], sexy=cons[,paste("sd",nmel12,sep="_")],colb=col,lg=0.01*rge_plot ) }# sd

# index

mtext(side=3, paste("Isotopic Evenness=",round(ID['IEve'],3),sep=""), at=mean(lim_1), line=0.5, cex=0.8,adj=0.5, font=2)

###############################################################

# Isotopic Uniqueness

# isotopic space given axes limits set using consumers signature

isotopic_space(nmX=tit1,nmY=tit2, limX=lim_1, limY=lim_2, labX=lab_1,labY=lab_2 )

# abundances, scaling: point area proportional to relative abundance, if relab=100%, circle diameter=15% of axis range

sizeab<-sqrt(rel_weight)*0.075*rge_plot

symbols(si[,nmel1],si[,nmel2], circles=sizeab, inches=FALSE, bg=col, fg=col, add=TRUE)

# distance to nearest neighbour

for (k in 1:nrow(NN))

{

arrows( si[k,nmel1],si[k,nmel2], si[which(NN[k,]==1)[1],nmel1], si[which(NN[k,]==1)[1],nmel2], col="black", lwd=1.8, length=0.1, angle=20)

} # end of k

# legend for abundance

rect(max(lim_1)-0.25*rge_plot, min(lim_2), max(lim_1), min(lim_2)+0.12*rge_plot)

symbols(max(lim_1)-0.19*rge_plot, min(lim_2)+0.06*rge_plot, circles=sqrt(0.1)*0.075*rge_plot, inches=FALSE, bg="black", fg="black", add=TRUE, lwd=1.5)

text(max(lim_1)-0.15*rge_plot, min(lim_2)+0.06*rge_plot,"10%", adj=c(0,0.5) )

# error bars if any

if(sum(nmelsd %in% colnames(cons) )==nbel) { meansexy(meanxy=si[,nmel12], sexy=cons[,paste("sd",nmel12,sep="_")],colb=col,lg=0.01*rge_plot ) }# sd

# index

mtext(side=3, paste("Isotopic Uniqueness=",round(ID['IUni'],3),sep=""), at=mean(lim_1), line=0.5, cex=0.8,adj=0.5, font=2)

###############################################

graphics.off()

} # end of e1, e2

} # end of plot

##############################################################################################

# returning results

return(ID)

} # end of function IDiversity

##################################################################################################################################################################

##################################################################################################################################################################

# 'IOverlap': function to compute isotopic overlap indices between two sets of organisms (e.g. life stages within a population, species within a community)

#

# INPUTS: - 'cons1' and 'cons2': two dataframes or matrices with stable isotope values for two sets of organisms (same format as for 'IDiversity').

# Elements names should be identical between the two sets and be a subset of ('d13C','d15N','dD','d34S').

# - 'nm_plot': a single character string specifying the name of the jpeg file where graphics illustrating isotopic diversity will be stored. Default is NA, i.e. no graphics.

# - 'col': a vector with 2 colors, coded as hexadecimal characters, for points and convex hull filling of the two sets of organisms. Default is 'cons1' in red and 'cons2' in blue.

# - 'transp': a single numeric value indicating the percentage of transparency for convex hulls filling. Default is 50%.

# - 'scaled': a logical value indicating wether isotopic values have been scaled to have a range between 0 and 1.

# If TRUE (default) axes of graphics fill at least the 0-1 range and axes titles specify the scaling procedure

#

# OUTPUTS: - a matrix with 5 indices (rows) for each element and for their combination (columns).

# Indices are Isotopic Richness (TRic1, IRic2) of the two sets respectively, the volume of their intersection (IRic1n2),

# Isotopic similarity (ISim), i.e. percentage of Isotopic Overlap expressed relatively to total Isotopic Richness [IRic1n2/(IRic1+IRic2-IRic1n2)],

# and Isotopic nestedness (INes), i.e. percentage of Isotopic Overlap expressed relatively to to minimum Isotopic Richness [IRic1n2/(min(IRic1,IRic2))]

#

# - a jpeg file (‘nm_plot’.jpeg) with one panel for each pair of elements .

# All axes have the same range to illustrate potential bias if no standardization are done.

# Points represent organism's position in the isotopic niche space (and associated standard deviation if values were provided in the corresponding 'cons' dataframe).

# Isotopic Overlap on each axis is illustrated by colored segment and as the intersection of colored polygons in the multidimensional space.

# If more than two elements were used to build the isotopic space, the convex polygons are projection of the multidimensional convex hulls in 2D.

#

##################################################################################################################################################################

IOverlap<-function(cons1,cons2,nm_plot=NA, col=c("#504AE8","#FA1900"), transp=50, scaled=TRUE) {

# names of elements used to describe consumers of set1

nmel_cons1<-colnames(cons1)[which(colnames(cons1) %in% nm_si)]

si_cons1<-as.matrix(cons1[,nmel_cons1])

# names of elements used to describe consumers of set2

nmel_cons2<-colnames(cons1)[which(colnames(cons2) %in% nm_si)]

si_cons2<-as.matrix(cons2[,nmel_cons2])

# checking that elements used to describe the 2 sets of consumers are identical

if ( sum(nmel_cons1 !=nmel_cons2) !=0 ) stop(" error: the 2 set of consumers should be described using the same elements")

nmel<-nmel_cons1 ; nbel<-length(nmel_cons1)

# checking number of consumers is higher than number of elements

if (nrow(cons1)<(nbel+1)) stop(paste(" error: computing indices using",nbel,"elements requires at least",nbel+1," consumers"))

##########################################################################################

# function to compute overlap between 2 sets of points in a multidimensional space

intersect<-function(set1,set2) {

# tranforming points coordinates in the Euclidean space in true rational number written as character string

# reduce set of points to vertices using redundant function

# changing polytope representation: vertices to inequality constraints

set1rep <- d2q(cbind(0, cbind(1, set1)))

polytope1 <- redundant(set1rep, representation = "V")$output

H_chset1 <- scdd(polytope1, representation = "V")$output

set2rep <- d2q(cbind(0, cbind(1, set2)))

polytope2 <- redundant(set2rep, representation = "V")$output

H_chset2 <- scdd(polytope2, representation = "V")$output

# intersection between the two polytopes

H_inter <- rbind(H_chset1, H_chset2)

V_inter <- scdd(H_inter, representation = "H")$output

# extracting coordinates of vertices

vert_1n2 <- q2d(V_inter[ , - c(1, 2)])

# computing convex hull volume of the intersection (if it exists)

vol_inter<-0

if (is.matrix(vert_1n2)) # vector if one vertex in common

if( nrow(vert_1n2)>ncol(vert_1n2) ) vol_inter<-convhulln(vert_1n2,"FA")$vol

return(vol_inter)

} # end of function intersect

##########################################################################################

# vector to store results

nD<-paste(nbel,"D",sep="")

IO<-matrix(NA,5,nbel+1, dimnames=list( c("IRic1","IRic2","IRic1n2","ISim","INes"), c(nmel,nD) ) )

# computing overlap on each axis

overlap1d<-function(x,y) { max(0, ( min(max(x),max(y)) - max(min(x),min(y)) ) ) }

for (e in nmel)

{

IO["IRic1",e]<-max(si_cons1[,e])-min(si_cons1[,e])

IO["IRic2",e]<-max(si_cons2[,e])-min(si_cons2[,e])

IO["IRic1n2",e]<-overlap1d( si_cons1[,e] , si_cons2[,e] )

IO["ISim",e]<-IO["IRic1n2",e]/(IO["IRic1",e]+IO["IRic2",e]-IO["IRic1n2",e])

IO["INes",e]<-( IO["IRic1n2",e] ) /min(c(IO["IRic1",e],IO["IRic2",e]))

} # end of e

# multidimensional IRic and vertices (if plot needed) for consumers of set1

IO["IRic1",nD]<-round(convhulln(si_cons1,"FA")$vol,6)

if( is.na(nm_plot)==FALSE) {

vert0<-convhulln(si_cons1,"Fx TO 'vert.txt'")

vert1<-scan("vert.txt",quiet=T)

vertices_cons1<-(vert1+1)[-1] } # end of vertices for plot

# multidimensional IRic and vertices (if plot needed) for consumers of set2

IO["IRic2",nD]<-round(convhulln(si_cons2,"FA")$vol,6)

if( is.na(nm_plot)==FALSE) {

vert0<-convhulln(si_cons2,"Fx TO 'vert.txt'")

vert1<-scan("vert.txt",quiet=T)

vertices_cons2<-(vert1+1)[-1] } # end of vertices for plot

# multidimensional intersection

IO["IRic1n2",nD]<-intersect(si_cons1, si_cons2 )

# multidimensional similarity= intersection/total volume

IO["ISim",nD]<-IO["IRic1n2",nD]/(IO["IRic1",nD]+IO["IRic2",nD]-IO["IRic1n2",nD])

# multidimensional nestedness= intersection/min(volume)

IO["INes",nD]<-IO["IRic1n2",nD]/min(c(IO["IRic1",nD],IO["IRic2",nD]))

######################################################################################

# graphical output

if( is.na(nm_plot)==FALSE) {

#####################################

# creating jpeg file

nmjpeg<-paste(nm_plot,".jpeg",sep="")

# one panel per pairs of elements

if( nbel==2) { jpeg(file=nmjpeg, res=150, width=1800, height=600) ; layout(matrix(c(1,0,0),1,3,F)) ; layout.show(1) }

if( nbel==3) { jpeg(file=nmjpeg, res=150, width=1800, height=600) ; layout(matrix(c(1:3),1,3,F)) ; layout.show(3) }

if( nbel==4) { jpeg(file=nmjpeg, res=150, width=1800, height=1200) ; layout(matrix(c(1:6),2,3,F)) ; layout.show(6) }

# setting axes limits given consumers signature for all elements

nmelsd<-paste("sd",nmel,sep="_")

cons<-rbind(cons1,cons2)

min_axes<- apply(cons[,nmel], 2, min, na.rm=T) ; max_axes<- apply(cons[,nmel], 2, max, na.rm=T) # limits of each axis

# limits of each axis given sd

if(sum(nmelsd %in% colnames(cons) )==nbel)

{min_axes<-apply(cons[,nmel]-cons[,nmelsd], 2, min, na.rm=T)

max_axes<-apply(cons[,nmel]+cons[,nmelsd], 2, max, na.rm=T) }

# same range on each axis for graphics: min=observed minimal value - 5% maximal range ; max=observed minimal value + maximal range + 5% maximal range

rge_axes<-max_axes-min_axes # range on each axis

newlim_axes<-matrix(0,length(nmel),2, dimnames=list(nmel,c("min","max") ) )

newlim_axes[,"min"]<-min_axes-max(rge_axes)*0.1

newlim_axes[,"max"]<-min_axes+max(rge_axes)*1.1

rge_plot<-max(rge_axes)*1.2

###############################################################

# plot for each pair of elements

for (e1 in 1:(nbel-1))

for (e2 in (e1+1):nbel)

{

# names of elements

nmel1<-nmel[e1] ; eval(parse(text=paste("tit1<-tit_",nmel1,sep="") ) )

nmel2<-nmel[e2] ; eval(parse(text=paste("tit2<-tit_",nmel2,sep="") ) )

nmel12<-c(nmel1,nmel2)

# limits of axes

lim_1<-newlim_axes[nmel1,]

lim_2<-newlim_axes[nmel2,]

# if axes are for scaled isotope values, "Scaled" in axis title and range is at least from 0 to 1

if (scaled==TRUE) {

tit1<-eval(parse(text=paste("tit1<-scl_tit_",nmel1,sep="") ) ); tit2<-eval(parse(text=paste("tit2<-scl_tit_",nmel2,sep="") ) )

lim_1<-c( min(-0.05, lim_1[1]) , max(1.05,lim_1[2]) )

lim_2<-c( min(-0.05, lim_2[1]) , max(1.05,lim_2[2]) )

} # end of if scaled axes

# labels on axes

lab_1<-pretty(lim_1,n=6,min.n=6) ; lab_1<-lab_1[which(lab_1>=lim_1[1] & lab_1<=lim_1[2])]

lab_2<-pretty(lim_2,n=6,min.n=6) ; lab_2<-lab_2[which(lab_2>=lim_2[1] & lab_2<=lim_2[2])]

# isotopic space given axes limits set using consumers signature

isotopic_space(nmX=tit1,nmY=tit2, limX=lim_1, limY=lim_2, labX=lab_1,labY=lab_2 )

# consumers of set1

# range on each axis

dec1<-rge_plot*0.015

segments( min(si_cons1[,nmel1]), min(lim_2)+dec1, max(si_cons1[,nmel1]), min(lim_2)+dec1, col=col[1] , lwd=3) # x

segments( min(lim_1)+dec1, min(si_cons1[,nmel2]), min(lim_1)+dec1, max(si_cons1[,nmel2]), col=col[1] , lwd=3) # y

# projected convex hull in 2D

vert0<-convhulln(si_cons1[,nmel12],"Fx TO 'vert.txt'")

vert1<-scan("vert.txt",quiet=T) ; vertices2D<-(vert1+1)[-1]

polygon(si_cons1[vertices2D,nmel12],border=NA,col=paste(col[1],transp,sep="") )

# all points (empty) then filling points being vertices in nD

points(si_cons1[,nmel12], pch=21,bg=NA, col=col[1],cex=2)

points(si_cons1[vertices_cons1,nmel12], pch=21,bg=col[1], col=col[1],cex=2)

# error bars if any

if(sum(nmelsd %in% colnames(cons1) )==nbel) meansexy(si_cons1[,nmel12], cons1[,paste("sd",nmel12,sep="_")],colb=col[1],lg=0.01*rge_plot )

# consumers of set2

# range on each axis

dec2<-rge_plot*0.03

segments( min(si_cons2[,nmel1]), min(lim_2)+dec2, max(si_cons2[,nmel1]), min(lim_2)+dec2, col=col[2] , lwd=3) # x

segments( min(lim_1)+dec2, min(si_cons2[,nmel2]), min(lim_1)+dec2, max(si_cons2[,nmel2]), col=col[2] , lwd=3) # y

# projected convex hull in 2D

vert0<-convhulln(si_cons2[,nmel12],"Fx TO 'vert.txt'")

vert1<-scan("vert.txt",quiet=T) ; vertices2D<-(vert1+1)[-1]

polygon(si_cons2[vertices2D,nmel12],border=NA,col=paste(col[2],transp,sep=""))

# all points (empty) then filling points being vertices in nD

points(si_cons2[,nmel12], pch=21,bg=NA, col=col[2],cex=2)

points(si_cons2[vertices_cons2,nmel12], pch=21,bg=col[2], col=col[2],cex=2)

# error bars if any

if(sum(nmelsd %in% colnames(cons2) )==nbel) meansexy(si_cons2[,nmel12], cons2[,paste("sd",nmel12,sep="_")],colb=col[2],lg=0.01*rge_plot )

# indices values

mtext(side=3, paste("Isotopic Similarity=",round(IO['ISim',nD],3),sep=""), at=mean(lim_1), line=0.9, cex=0.8,adj=0.5, font=2)

mtext(side=3, paste("Isotopic Nestedness=",round(IO['INes',nD],3),sep=""), at=mean(lim_1), line=-0.3, cex=0.8,adj=0.5,font=2)

} # end of e1, e2

graphics.off()

} # end of plot

###############################################

# returning results

return(IO)

} # end of IOverlap

##############################################################################################################################################

################################ END OF SRIPT ############################################

##############################################################################################################################################
